# Cryopreservation moderates the thrombogenicity of arterial allografts during storage

**DOI:** 10.1371/journal.pone.0255114

**Published:** 2021-07-22

**Authors:** László Hidi, Erzsébet Komorowicz, Gergely Imre Kovács, Zoltán Szeberin, Dávid Garbaisz, Natalia Nikolova, Kiril Tenekedjiev, László Szabó, Krasimir Kolev, Péter Sótonyi

**Affiliations:** 1 Department of Vascular and Endovascular Surgery, Heart and Vascular Center, Semmelweis University, Budapest, Hungary; 2 Department of Biochemistry, Semmelweis University, Budapest, Hungary; 3 Department of Information Technology, Nikola Vaptsarov Naval Academy, Varna, Bulgaria; 4 Australian Maritime College, University of Tasmania, Launceston, Australia; 5 Department of Functional and Structural Materials, Institute of Materials and Environmental Chemistry, Research Centre for Natural Sciences, Hungarian Academy of Sciences, Budapest, Hungary; University of Toledo, UNITED STATES

## Abstract

**Introduction:**

Management of vascular infections represents a major challenge in vascular surgery. The use of cryopreserved vascular allografts could be a feasible therapeutic option, but the optimal conditions for their production and use are not precisely defined.

**Aims:**

To evaluate the effects of cryopreservation and the duration of storage on the thrombogenicity of femoral artery allografts.

**Methods:**

In our prospective study, eleven multi-organ-donation-harvested human femoral arteries were examined at five time points during storage at -80°C: before cryopreservation as a fresh native sample and immediately, one, twelve and twenty-four weeks after the cryopreservation. Cross-sections of allografts were perfused with heparin-anticoagulated blood at shear-rates relevant to medium-sized arteries. The deposited platelets and fibrin were immunostained. The thrombogenicity of the intima, media and adventitia layers of the artery grafts was assessed quantitatively from the relative area covered by fibrin- and platelet-related fluorescent signal in the confocal micrographs.

**Results:**

Regression analysis of the fibrin and platelet coverage in the course of the 24-week storage excluded the possibility for increase in the graft thrombogenicity in the course of time and supported the hypothesis for a descending trend in fibrin generation and platelet deposition on the arterial wall. The fibrin deposition in the cryopreserved samples did not exceed the level detected in any of the three layers of the native graft. However, an early (up to week 12) shift above the native sample level was observed in the platelet adhesion to the media.

**Conclusions:**

The hemostatic potential of cryopreserved arterial allografts was retained, whereas their thrombogenic potential declined during the 6-month storage. The only transient prothrombotic change was observed in the media layer, where the platelet deposition exceeded that of the fresh native grafts in the initial twelve weeks after cryopreservation, suggesting a potential clinical benefit from antiplatelet therapy in this time-window.

## Introduction

Despite modern sterilisation processes, antibiotic therapies and wound treatments, the management of vascular infections remained a major challenge for vascular surgery. The use of cryopreserved vascular allografts could be a feasible therapeutic option in these cases. However, despite the clinical use, no strong evidence-based guidelines of the production and use of cryopreserved vascular tissues have been issued so far. There are many different cryopreservational strategies, all assuming that the storage time is virtually infinite [1]. Limited data are available concerning the relationship between the quality of allografts and their cryopreservation storage time. Wang and al., who investigated living donor liver transplantation, found no differences in the patency of vascular grafts for outflow reconstruction or the regeneration of liver graft between iliac allografts with less or more than one-year cryopreservation storage time [2]. However, our earlier report on the use of allografts highlighted the superiority in patency and limb salvage of the allografts stored for less than 6 months after cryopreservation in arterial reconstruction of lower extremities [3]. To the best of our knowledge no data on the relationship between allografts and their cryopreservation storage time is available in the literature other than the above two articles.

In addition, the occurrence of early occlusion (within 30 days) of the allografts varies in a broad range between 0–17% [3–11], which could be traced back to effects of multiple factors, such as anatomical location or quality of the allograft. The patency of the implanted grafts strongly depends on the preservation of the inherent thromboresistance of the native arterial wall, i.e. their capacity to prevent intravascular blood clot formation, the key companions of which are platelets and fibrin [12]. However, platelet deposition and fibrin generation by the plasma coagulation system are essential factors of the hemostatic function of the vascular wall to prevent bleeding at the site of the implanted graft. Thus, the implementation of optimal manufacturing and quality control of allografts in vascular surgery requires refined understanding of the balance of thrombogenic and hemostatic potential of the applied allografts.

In line with this timely demand, the aim of this study was to evaluate the effects of cryopreservation and the duration of storage on the deposition of platelets and fibrin in the vessel wall as indicators of the thrombogenicity of femoral artery allografts.

## Materials and methods

In our prospective study eleven human femoral arteries, which were harvested from eleven different donors in multi-organ donations, were examined at five time points: before the cryopreservation as a native sample (BC) and after the cryopreservation immediately (C0), on the first (C1), twelfth (C12) and twenty-fourth (C24) week of storage after cryopreservation.

### Donor characteristics

The median age of the donors was 45.0(33.0;50.5) years. Three donors were female (27.27%). The median body mass index (BMI) was 26.3(23.65;27.8) kg/m^2^. The leading cause of death was cerebral haemorrhage (6 donors; 54.54%). All essential characteristics of the donors are presented in Table 1.

**Table 1:** Donor characteristics.

Characteristic		n = 11
**Cause of death**	**trauma**	3(27.27)
	**cerebral ischaemia**	2(18.18)
	**cerebral haemorrhage**	6(54.55)
**Age (year)**		45.00(33.00–50.50)
**Female sex**		3(27.27)
**BMI (kg/m2)**		26.30(23.65–27.80)
**Past medical history**	**Hypertension**	3(27.27)
	**Diabetes**	1(9.09)
	**Pulmonary disease (COPD)**	1(9.09)
	**Smoking**	4(36.36)
**Blood type**	**A**	5(45.46)
	**B**	3(27.27)
	**AB**	0(0)
	**0**	3(27.27)
	**Rh+**	10(90.91)
	**Rh-**	1(9.09)

Data are presented as number of donors(%) or median(lower quartile–upper quartile). n: number of donors; BMI: body mass index; COPD: chronic obstructive pulmonary disease.

### Inclusion and exclusion criteria of allografts

Donor inclusion criteria of the study were based on the national multi-organ donation criteria and rules [13]. The allografts from donors above 65 years, with malignancy or with positive bacteria-fungal culture or virus serology test and the allografts with negative evaluation of the vascular surgeon, who performed the explantation or cryopreservation, (e.g. significantly injured or calcified allograft) were excluded.

### Ethical considerations, data collection

The study was in full compliance with the principles of the national multi-organ donation and the applicable international and national laws. The anonymous data of the donors were collected prospectively from the electronic health information system of the donation according to the General Data Protection Regulation of the European Union. The study was approved by the institutional review board, by Semmelweis University Regional and Institutional Committee of Science and Research Ethics (approval number: 257/2018.). The consents were not obtained, because the vascular tissues were harvested from brain-dead donors in multi-organ donations and the data were analyzed anonymously.

### Harvesting, cryopreservation, storage and thawing of allografts

Femoral arteries were harvested in Budapest from brain-dead donors in multi-organ donations organized by the Organ Coordination Office of the Hungarian National Blood Transfusion Service. All donors had negative serology test of human immunodeficiency virus, hepatitis B virus, hepatitis C virus, syphilis, active Epstein-Barr virus and active cytomegalovirus. The explantation of the femoral arteries (common and superficial femoral artery) was performed under surgical asepsis and principles of sterile technique. The suitability of the grafts was evaluated by the vascular surgeon, who performed the harvesting. After the explantation the allografts were placed immediately into a triple sterile plastic bag (Set of Transplantation Bags–sterile 80 00 61H, Raguse GmbH, Ascheberg, Germany) in 500 ml transport solution (Sodium Chloride 0.9% “Baxter” Intravenous Infusion in Viaflo, Baxter Hungary, Budapest, Hungary) containing 4 mg/ml cefazolin (Sandoz GmbH, Kundl, Austria) and 0.4 mg/ml fluconazole (Fresenius Kabi Hungary, Budapest, Hungary) at 4°C. They were transferred in an organ transport box (IGLBox Organ Transporter, Institut Georges Lopez, Lissieu, France) at 4°C to the site of storage and were stored for 12 h at 4°C.

**Fig 1:**
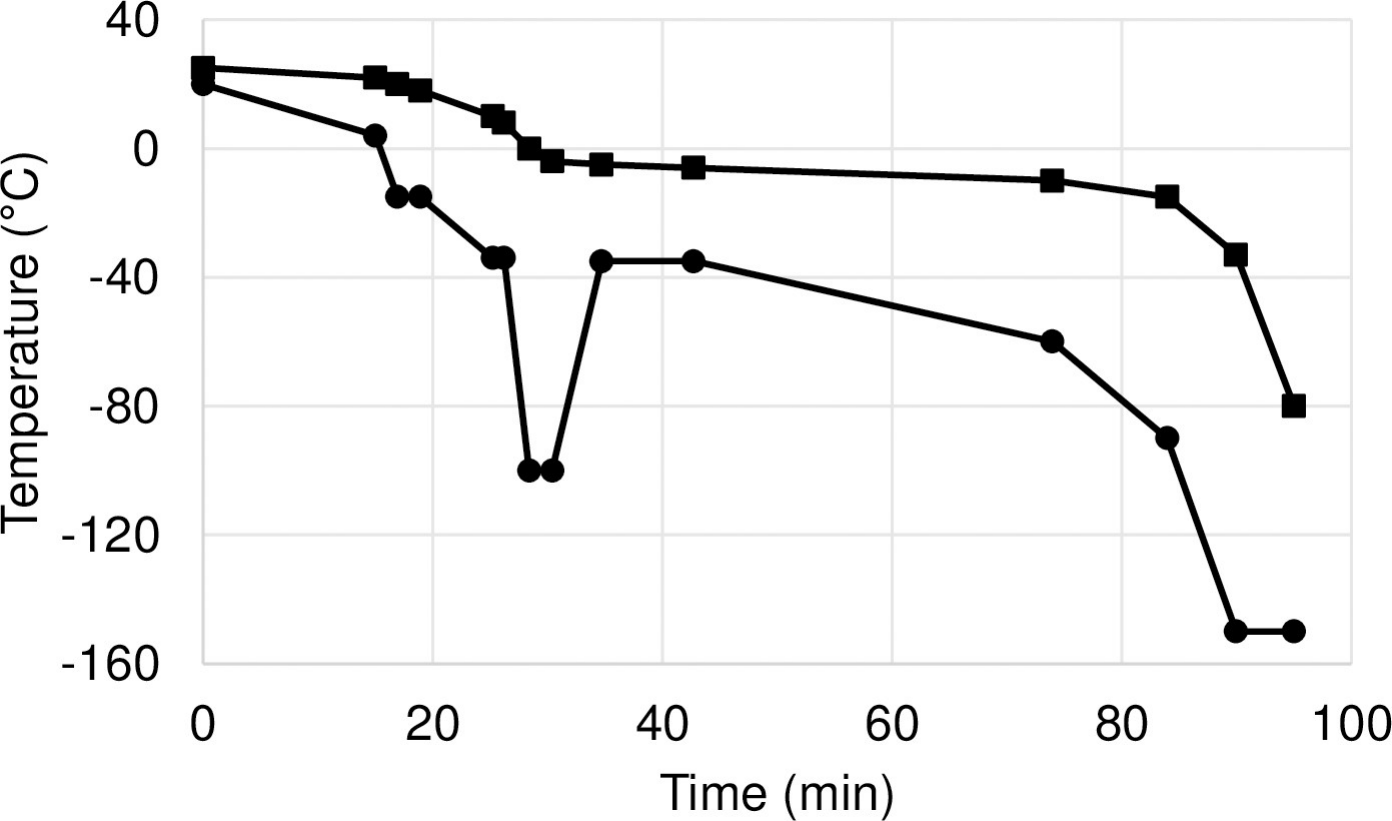
Controlled freezing protocol of the cryopreservation. Temperature ramps of controlled-rate freezer’s chamber: continuous line with “●”; temperature ramps of sample: continuous line with “■”.

The cryopreservation was performed within 12 h after the explantation in a clean room classified “A” with a background classified “B” used laminar air flow system [14]. After performing bacteria-fungal culture test and reevaluation of the grafts, five 0.5 cm wide ring samples were cut from each femoral artery. One of the samples (BC) was placed into a plastic cryotube in isopentane (Merck Kft, Budapest, Hungary) and stored at -20°C. The rest of the samples (C0, C1, C12, C24) underwent the same cryopreservation procedure used for the allografts. They were placed in cryobags (TissueVault Cryogenic Freezing Bag TV1430, Origen Biomedical, Austin, Texas, USA) in a 500 ml cryopreservation solution (Ringer Fresenius, Fresenius Kabi Deutschland GmbH, Bad Homburg, Germany) containing 20 v/v% dimethyl sulfoxide (Molar Chemicals Kft., Halásztelek, Hungary), 4 mg/ml cefazolin (Sandoz GmbH, Kundl, Austria) and 0.4 mg/ml fluconazole (Fresenius Kabi Hungary, Budapest, Hungary) for 10 minutes. Thereafter, these samples were cryopreserved to -80-90°C according to a controlled freezing method (PC Interface for Thermo Scientific^TM^ Cryomed^TM^ Freezers, Version 3.0 and Thermo Scientific CryoMed Controlled-Rate Freezer 7451, Thermo Fisher Scientific Inc., Waltham, Massachusetts, USA) (Fig 1) using vaporised nitrogen (Messer Hungarogáz Kft., Budapest, Hungary), and they were stored at -80°C in a deep freezer (Taylor-Wharton K Series Cryogenic Storage System 10K, Taylor-Wharton, Baytown, Texas, USA).

For the thrombogenicity measurements after various storage periods, the cryopreserved graft samples were thawed at 37°C until the disappearance of the ice components and washed three times with physiological salt solution (Sodium Chloride 0.9% “Baxter” Intravenous Infusion in Viaflo, Baxter Hungary, Budapest, Hungary) at 20°C to remove the cryopreservation medium.

### Measurement of thrombogenicity with immunofluorescence imaging

The flow-chamber model described previously was used for testing the allograft samples as thrombogenic surfaces [15, 16]. Frozen cross-sections (10 μm) of allograft samples placed on poly-L-Lys-coated slides were perfused at 0.5 ml/min flow rate in a 0.4-cm-wide and 0.12-mm-high parallel-plate chamber with heparin-anticoagulated blood collected from healthy volunteers. Assuming laminar flow conditions, the shear rate at the surface of the section was 900 s^−1^, according to the formula 1.03*6 Q/(w*h^2^), where Q is the flow rate in ml s^−1^, w and h are the width and the height of the flow path in cm, respectively. This intermediate shear rate was chosen as an adequate model of the rheological situation in medium-sized arteries, where deposition of both platelets and fibrin is enabled [17]. Before the perfusion the sections were blocked with 2 w/v% bovine serum albumin (BSA) in 0.05 M Tris buffer pH 7.4 containing 0.1 M NaCl and 0.02 w/v% NaN_3_ (BSA-TBS) for 45 minutes and the 90-s perfusion was followed by a 30-s wash with 1.5 mM KH_2_PO_4_, 8.1 mM Na_2_HPO_4_ buffer pH 7.4 containing 137 mM NaCl and 2.7 mM KCl (PBS). Thereafter the sections were fixed in acetone at 4°C for 10 minutes and the deposited platelets and fibrin were double-stained for indirect immunofluorescence microscopy. Sections were blocked with BSA-TBS for 30 min followed by platelet staining using the mouse monoclonal antibody against human GpIIb/IIIa (4 μg/ml in BSA-TBS for 30 min, sc-53417, Santa Cruz Biotech), followed by 3 times 5 min washes in TBS, and 30 min incubation with the Goat-anti-Mouse IgG-Alexa Fluor 633 secondary antibody (2 μg/ml in BSA-TBS, Invitrogen, Budapest, Hungary). Double-staining was continued with the fibrin staining after 3 times 5 min washes in BSA-TBS: 30 min incubation with the primary rabbit polyclonal antibody developed against the N-terminal part of the gamma chain fibrin(ogen) (20 μg/ml in BSA-TBS, PA5-29734, Invitrogen), followed by 3 times 5 min washes in TBS, and 30 min incubation with the Goat-anti-Rabbit IgG-Alexa Fluor 546 secondary antibody (2 μg/ml in BSA-TBS, Invitrogen). Following final washes in TBS, the stained sections were covered in 50% glycerol in TBS. Confocal images were taken from the slides using a Zeiss LSM710 confocal laser scanning microscope equipped with a 10 × 0.3 lens (Carl Zeiss, Jena, Germany) using 488 nm, 543 nm and 633 nm excitation laser lines, respectively, and emissions were detected in the ranges of 500–530 nm, 565–585 nm and 650–690 nm, respectively. Each allograft vessel at each sampling time point was perfused in duplicates or triplicates and depending on the section size, 5–10 different images were taken of each perfused cryosection in order to survey the whole cross-sectional area of the vessel. Quantification of platelet and fibrin(ogen) coverage of the vessel wall was performed with the Image J software (NIH, Bethesda, MD, USA) selecting the region of interest, calculating its surface area in pixels and setting a threshold intensity value for automatic identification of platelets or fibrin(ogen) covered areas in percentage.

### Scanning Electron Microscope (SEM) imaging

Two consecutive cryosections (10 μm) of each allograft sample were placed on Thermanox coverslips and perfused with heparinized whole blood as described for the immunofluorescent imaging above. The perfused sections were cut out of the plastic coverslips with scissors, fixed and processed for scanning electron microscopy. Following repeated washes with 100 mmol/L Na-cacodylate pH 7.2 buffer, samples were fixed in 1 v/v% glutaraldehyde for 16 h. The fixed samples were dehydrated in a series of ethanol dilutions (20–96 v/v%), 1:1 mixture of 96 v/v% ethanol/acetone and pure acetone followed by critical point drying with CO2 in E3000 Critical Point Drying Apparatus (Quorum Technologies, Newhaven, UK). The specimens were mounted on adhesive carbon discs, sputter coated with gold in SC7620 Sputter Coater (Quorum Technologies, Newhaven, UK) and images were taken with scanning electron microscope EVO40 (Carl Zeiss GmbH, Oberkochen, Germany) at 5000x magnification from the intima, media and adventitia layer of each cross-section.

### Statistical analysis

Because our immunofluorescence measurements were performed on arterial samples from 11 donors, in each of which we evaluated the platelet and fibrin content from different number of collected data (in the range 111–225), we used a fuzzy sample approach [18] to evaluate the data. This approach allows for the achievement of parity of the arterial samples from different donors, as applied previously [19–21]. Here, we implemented a novel explicit fuzzy estimation method and a novel fuzzy version of the invertible cumulative distribution function estimator with maximum count of nodes [22] for the implicit estimation of the median, lower quartile and upper quartiles of a random variable. A qualitative quartile-wise estimation of the effect of cryopreservation on the thrombogenicity was based on comparisons of coverage values at each time points and the native graft. In this qualitative analysis we tested the null hypothesis *H*_*0*_ (that the compared quartiles are equal) against the alternative hypothesis *H*_*1*_ (that one of the quartiles is greater than the other one) using four different Bootstrap one-tailed tests [23] with modifications detailed in S1 File. A cluster approach to hypothesis testing was adopted to increase the statistical power of the tests [24]: each of the four p-values were compared to the predetermined significance level *α* = 0.05 and the null hypothesis was rejected, if at least two out of the four p-values were less than *α*. A quantitative analysis of the effect of storage time on the thrombogenicity was based on the identification of the trends of changes in the median, lower quartile and upper quartile of the thrombogenic factor abundance in the course of time. For each of the three quartiles we constructed a linear regression with the time as independent variable trained on a fuzzy sample containing four triplets in the form (time, quartile, degree of membership) using a previously described analytical algorithm [25]. A cluster of four fuzzy Bootstrap procedures described in S1 File were used to identify the distribution of the predicted regression slope and the probabilities for the slope to be negative and non-negative (or positive and non-positive). We considered the negativity/positivity of the estimated regression slope to be significant, if at least two of the four fuzzy Bootstrap procedures indicated probability for non-negativity/non-positivity less than the preselected significance level *α* = 0.05. Such estimates based on probabilities are in line, but superior to the p-values of any statistical test in their power to determine the significance of the identified slope sign, because they calculate the probability of being right when accepting the alternative hypothesis, whereas the p-values provide only the probability of being wrong when rejecting the null hypothesis.

## Results

**Fig 2:**
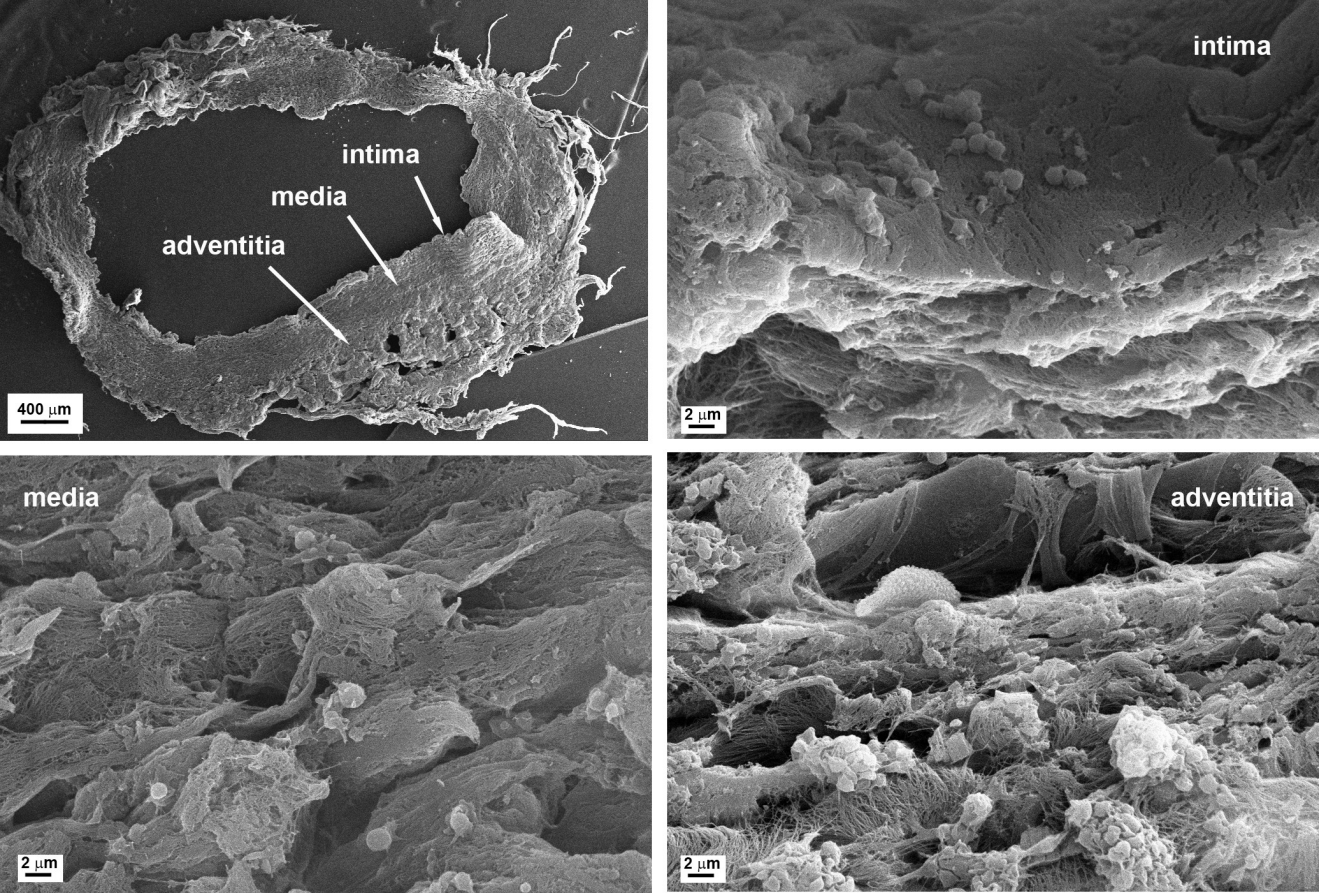
Platelet adhesion to arterial cross-sections. Cross-sections of the arterial grafts were prepared as described in the Materials and Methods (top left panel) and perfused with heparinized blood. Following the perfusion, the cross-sections were fixated and processed for scanning electron microscopic imaging. Isolated adhered platelets are seen in the intima and media layers, whereas larger aggregates are formed in the adventitia.

**Fig 3:**
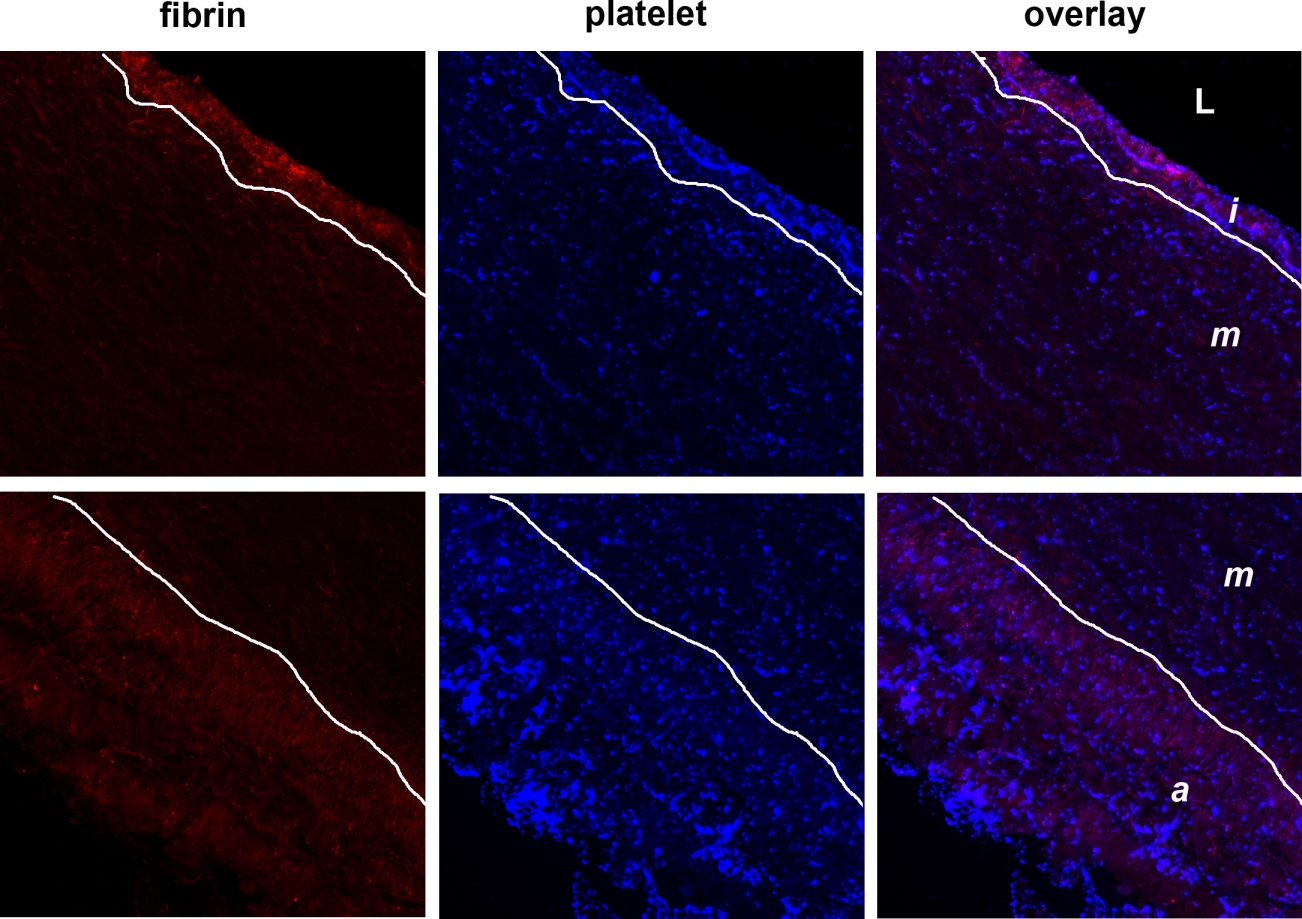
Platelet and fibrin deposition to arterial cross-sections detected by indirect immunofluorescence. Cryosections of allograft samples were perfused with heparinized whole blood and deposited platelets and fibrin were visualized with double immunofluorescence as detailed in Materials and Methods. Representative images illustrate the uneven spatial distribution of GpIIb/IIIa (blue) and fibrin (red) staining in the three layers of the perfused arterial cross-sections. For quantitative evaluation platelet and fibrin surface coverage data were collected from 5–10 such images (original size of the presented images 850 μm × 850 μm) to survey the whole size of the perfused cross-section, and the intrinsic green autofluorescence of elastin (Fig 4, here indicated by the white lines) was used as a guide to encircle the regions of interest and evaluate the three vessel wall layers separately. l: lumen; i: intima; m: media; a: adventitia.

We assessed the effects of our allograft-preservation method on the thrombogenicity of the arterial allografts based on the adhesion of platelets and generation of fibrin in the three layers of the grafts (adventitia, media, intima). When cross-sections of the grafts were perfused with heparinized blood at shear rate equivalent to the wall shear rate in medium-size arteries, platelets adhered to the arterial wall in a highly heterogeneous pattern, distinct in the three layers (Fig 2). In order to account for this heterogeneity in a quantitative manner, we applied a confocal microscopic technique to detect the immunofluorescence signal of a platelet-related antigen (GpIIb/IIIa receptor) on larger areas (lower magnification than SEM) and multiple (5–10) regions of interest in each layer separately (Fig 3). Because the thrombogenic potential of the arterial wall depends not only on the recruitment of platelets, but also on the activation of blood coagulation, we evaluated the deposition of fibrin as an end-product of the blood clotting cascade on the graft cross-sections with anti-fibrin antibody in the same immunofluorescence microscopic assay (Fig 3).

### Fibrin deposition to the artery wall

**Fig 4:**
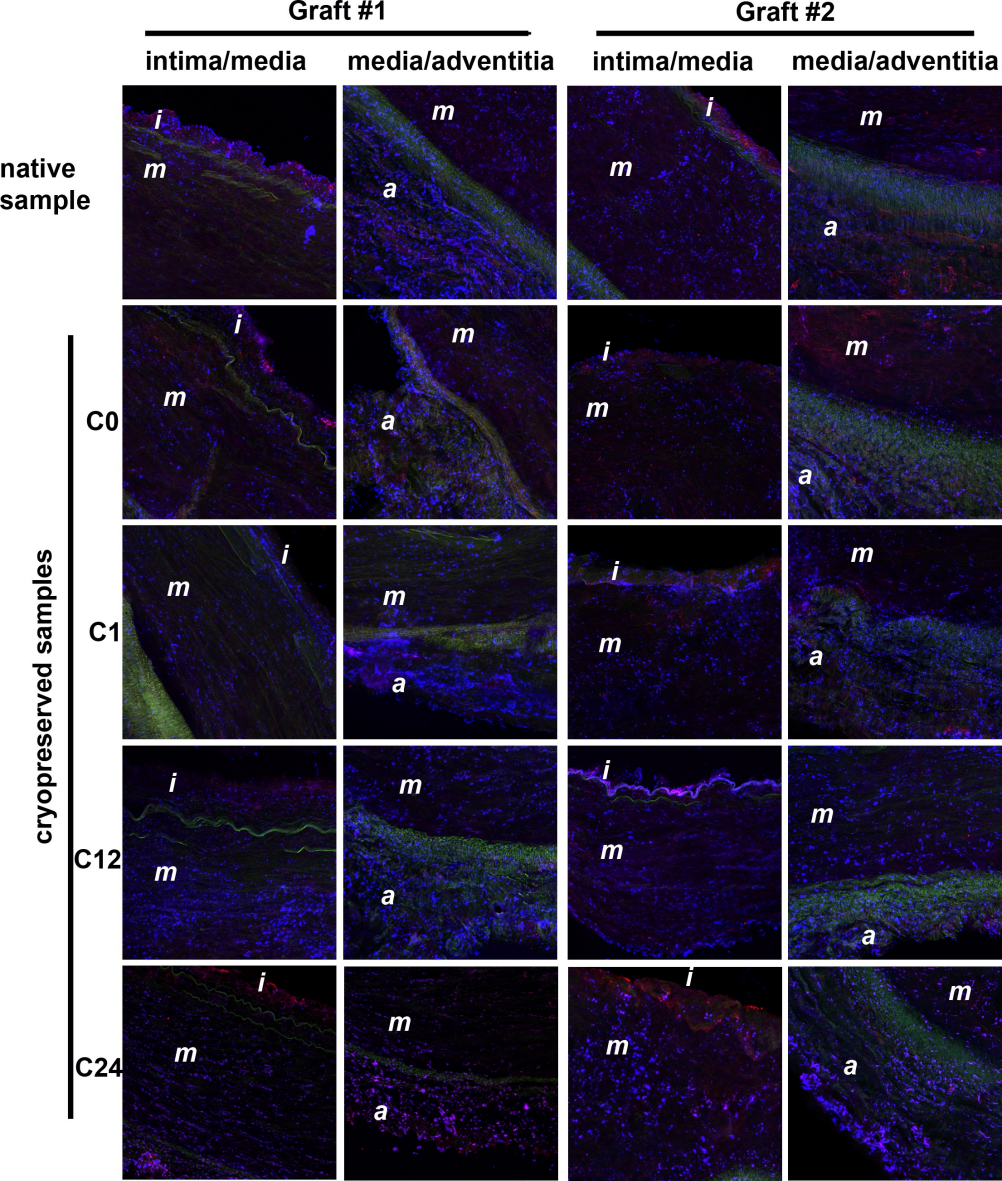
A time lapse sequence of immunofluorescence images monitoring the fibrin and platelet deposition in different layers of the arterial wall. Cryosections of allografts were immunostained for platelet (blue) and fibrin (red) antigen after whole blood perfusion as in Fig 3. The green autofluorescence of the internal and external elastic lamina indicates the boundaries between the wall layers (i: intima; m: media; a: adventitia). Images from two different regions of interest in two different allografts are shown for each time point.

**Fig 5:**
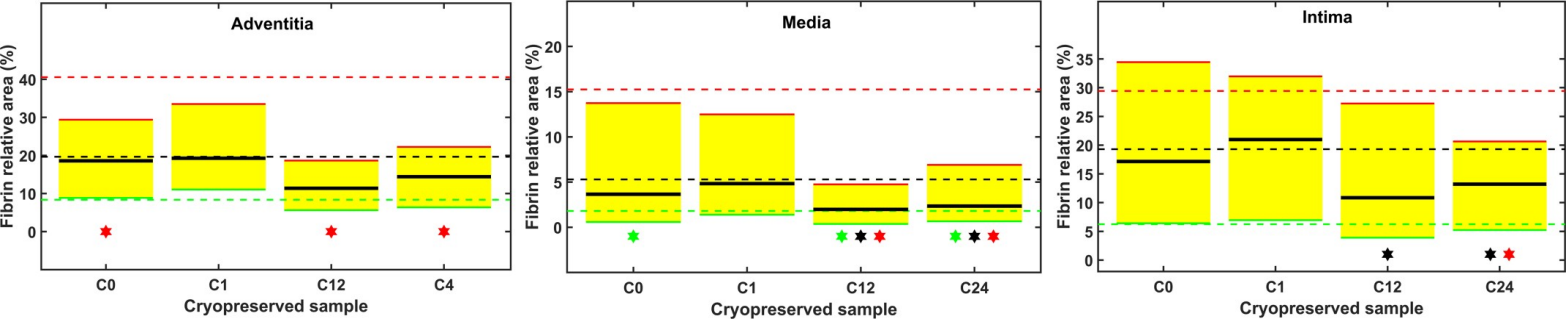
Qualitative quartile-wise comparison of the fibrin deposition on cryopreserved and native arterial allografts. Fibrin coverage of the separate arterial wall layers was measured as illustrated in Fig 3. Lower (green), median (black) and upper (red) quartiles of the measured coverage data are shown with dashed lines for the BC and continuous lines for the cryopreserved samples. The interquartile range of the cryopreserved samples is shadowed in yellow. Symbols indicate significant differences between the respective quartiles (green, lower quartile; black, median; red, upper quartile) of the BC and cryopreserved samples according to the cluster statistical approach described in Materials and Methods.

In quantitative terms the generation of fibrin was assessed as percentage of area of the intima, media and adventitia layers covered by fibrin in the immunofluorescent images of the graft cross-sections (Fig 4). The statistical analysis did not support any significant increase of fibrin deposition in the cryopreserved samples over the fibrin level detected in any of the three layers of the native (BC) samples (Fig 5). The subset of fibrin coverage data in the interquartile range of the cryopreserved grafts (yellow shadowed area, Fig 5) largely overlapped with the respective band of the BC samples (the area between the red and green dashed lines, Fig 5) without any single significant deviation of a quartile boundary of a cryopreserved sample over a BC sample. It is noteworthy that all significant differences between cryopreserved and native graft samples indicated reduced fibrin generation (out of the 12 sets of cryopreservation data 3 median, 3 bottom quartile and 6 top quartile values were lower than their BC counterparts, Fig 5). Cryopreservation was associated also with a trend to homogenize the fibrin generation potential of the artery wall layers (narrower interquartile range in 8 out of the 12 cryopreservation data sets).

**Fig 6:**
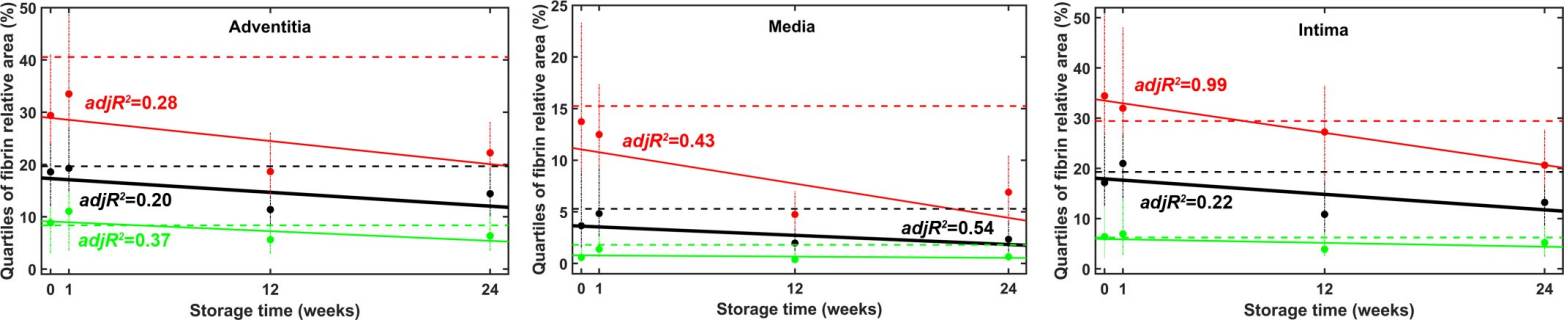
Time course of fibrin deposition in cryopreserved and native arterial allografts. Fibrin coverage of the separate arterial wall layers was measured as illustrated in Fig 3. Lower (green), median (black) and upper (red) quartiles of the measured coverage data are shown with dashed lines for the BC, symbols for the cryopreserved samples and continuous lines for the linear regression performed as described in Materials and Methods. Error bars (vertical dotted lines) indicate the data spanning the range of 12.5% rank below and above the respective quartile value. The explanatory strength of the correlation can be assessed on the basis of the adjusted R^2^ (adjR^2^) values that are shown only for the regression trends proven to be significant at 0.05 level by the statistical approach described in Materials and Methods.

In order to test the hypothesis that the storage time affects the fibrin deposition to the cryopreserved grafts, we performed regression analysis of the changes in fibrin coverage on the three arterial wall layers as a function of time (Fig 6). In seven out of the nine hypothesis tests performed to check the temporal trend of the fibrin coverage in the three quartiles of the three wall layers a non-negative trend was excluded at 0.05 significance level, whereas in the rest two cases–the lower coverage quartile of the media and intima layers–the probability of non-negative trend was 20–22% and 14–28%, respectively. Thus, the robust statistical analysis of the fibrin coverage data in the course of twenty-four-week storage of the arterial grafts definitely excludes the possibility for increase in the graft thrombogenicity in the course of time and strongly supports the decline in the fibrin generation.

### Platelet adhesion to the artery wall

**Fig 7:**
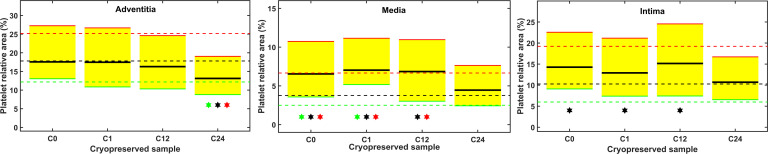
Qualitative quartile-wise comparison of the platelet adhesion on cryopreserved and native arterial allografts. Platelet coverage of the separate arterial wall layers was measured as illustrated in Fig 3. Lower (green), median (black) and upper (red) quartiles of the measured coverage data are shown with dashed lines for the BC and continuous lines for the cryopreserved samples. The interquartile range of the cryopreserved samples is shadowed in yellow. Symbols indicate significant differences between the respective BC and cryopreservation quartiles according to the cluster statistical approach described in Materials and Methods.

Platelet adhesion was assessed as percentage of area of the intima, media and adventitia layers covered by the GpIIb/IIIa-related immunofluorescence signal. Similar to fibrin deposition, the platelet coverage data in the interquartile range of the cryopreserved grafts (yellow shadowed area in Fig 7) largely overlapped with the respective band of the BC samples. In contrast to fibrin, however, in 3 out of the 12 datasets of cryopreserved samples all three (C0 and C1 of the media) or two (C12 of the media) of the quartile values exceeded the respective platelet coverage level of the native samples (BC) (Fig 7). In the early cryopreserved samples of the intima (C0, C1, C12), the median of the platelet coverage moved upward, but this difference was not accompanied by a shift in the upper quartile. The interquartile range of these samples largely overlapped with the BC interquartile range. Thus, the early changes in the platelet adhesiveness of the intima can be interpreted as resulting in moderate rearrangements in the platelet coverage within the variability of the non-cryopreserved grafts.

**Fig 8:**
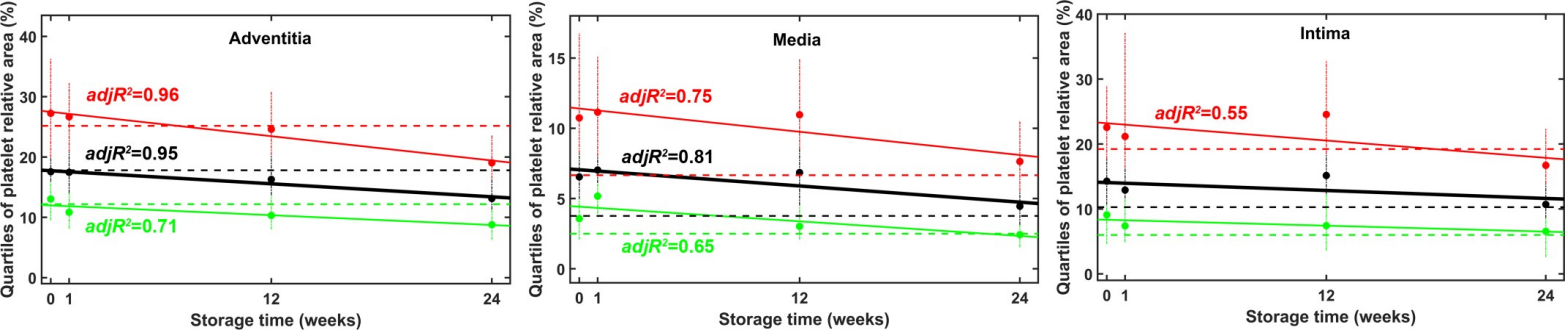
Time course of platelet adhesion to cryopreserved and native arterial allografts. Platelet coverage of the separate arterial wall layers was measured as illustrated in Fig 3. Lower (green), median (black) and upper (red) quartiles of the measured coverage data are shown with dashed lines for the BC, symbols for the cryopreserved samples and continuous lines for the linear regression performed as described in Materials and Methods. Error bars (vertical dotted lines) indicate the data spanning the range of 12.5% rank below and above the respective quartile value. The explanatory strength of the correlation can be assessed on the basis of the adjusted R^2^ (adjR^2^) values that are shown only for the regression trends proven to be significant at 0.05 level by the statistical approach described in Materials and Methods.

The regression analysis of the changes in platelet coverage of the three arterial wall layers as a function of time excluded a non-negative trend at 0.05 significance level in seven out of the nine hypothesis tests performed on the platelet coverage of the three quartiles of the three wall layers (Fig 8). The two exceptions were the median and the lower coverage quartile of the intima, where the probability of non-negative trend was 5–11% and 7–18%, respectively. Thus, the regression analysis of the platelet coverage data in the course of twenty-four-week storage of arterial grafts definitely excludes the possibility for time-dependent increase in the graft thrombogenicity and strongly supports the decline in the platelet adhesiveness.

## Discussion

The incidence of vascular surgical wound infection has a wide range, between 3.5% and 32%, and the risk of a prosthetic graft infection is 0.25–6% after the implantation. Moreover, these numbers can be higher in patients with infection-predisposing wound complications and in certain locations of the surgery [26–30]. In these cases, an optimal therapeutic modality would be a biocompatible, infection-resistant, easily accessible vascular graft. Autologous superficial veins are ideal solution for such implantations and they can give the best results for the reconstruction of the lower extremities, but 20–45% of the patients do not have any appropriate veins [7, 28, 31, 32]. In the absence of autologous veins, the cryopreserved allografts can be an alternative solution for the management of the vascular infection. However, because of the lack of evidence-based guidelines for the production and use of cryopreserved vascular tissues, characterization of the biomechanical, biochemical and immunological properties of the allografts are essential for the implementation of optimal manufacturing and quality control processes. The cryopreservation strategy is a crucial point of the investigation of allografts. Harvesting, cryopreservation, thawing are potentially harmful procedures for the grafts. There are many different methods by allograft banks, but their advantages and disadvantages are not fully elucidated [1, 4–6, 8, 10, 11, 33, 34].

The cryopreservation protocol applied in the current study maintained the procoagulant properties of the stored grafts at the level of their fresh native counterparts. In addition, a moderate, but consistent trend for time-related decline in fibrin deposition was observed. Although we did not investigate the mechanism of this anticoagulant trend, a hypothetic cause could be reduction of blood clotting triggers in the wall of stored grafts. Tissue factor, the major trigger of blood coagulation is known to lose a significant fraction of its activity during a six-month storage period in frozen state [35]. A practical implication of this result is that one cannot expect to improve the graft patency with anticoagulant prophylaxis, because the cryopreserved grafts do not trigger more massive fibrin formation than the native artery wall. This finding is in line with the lack of significant effect of warfarin on the primary graft patency of cryopreserved saphenous vein allografts reported by others [11].

Concerning the role of anti-platelet medication in graft patency, controversial results have been reported in previous studies. A definite benefit to patency from the anti-platelet therapy was seen in an animal model with saphenous vein allografts [36], but not in patients undergoing infrainguinal revascularization procedure [11]. Our current findings help the interpretation of this controversy. We propose that an anti-platelet strategy could be of advantage only within a certain time-window determined by the temporal pattern of changes in the platelet-adhesiveness of the stored cryopreserved grafts. In the early storage period in our study (up to 12 weeks after cryopreservation) platelet deposition in the media layer exceeded that of the media in fresh, non-cryopreserved grafts. This is probably the time frame of storage, when anti-platelet medication could be of benefit for the patency of the implanted graft. Out of this time window and in the intima and adventitia layers at all time-points the platelet deposition remained at the level observed prior cryopreservation, which implies a normal hemostatic function of the graft and does not justify any anti-platelet medication.

An important aspect of the current study is that we evaluated separately the thrombogenicity of the three layers of the artery wall, because these are known to exert differential effects on blood clotting and platelet function. According to our data the adventitia and intima layers showed a higher thrombogenic potential, while the media layer’s relative platelet and fibrin coverage lagged behind the other two layers. On the one hand, the decreased procoagulant potential of the media layer can be explained by the lower levels of tissue factor protein and mRNA in this layer, as reported earlier [37]. On the other hand, concerning the relatively lower platelet deposition in the media, our current findings are in agreement with earlier studies applying the same experimental setup that showed a limited von Willebrand factor-dependent binding of platelets to native media of arteries and the strongest adhesiveness of the adventitia [15, 16]. In a different experimental setup, the adventitia of normal, non-atherosclerotic vessel walls was observed similarly with the highest platelet deposition, but the subendothelium had the lowest one and the tunica media had an intermediate adhesiveness [38]. However, other studies established that the subendothelium can also cause a rapid and massive platelet activation more pronounced than the media [39, 40], as seen in our current and earlier work. Independently of the differences in the relative thrombogenicity of the three layers, following cryopreservation its time-related changes were invariably favourable in all of them. Layer-dependent response to cryopreservation was observed only in the media layer and these were restricted to the three earliest times of storage.

The limitations of this study could be the in vitro design, however it is the optimal first part of further investigations at a such multifactorial and undiscovered field for identification of the main issues. The most frequently used allograft material in vascular surgery is the great saphenous vein, especially by revascularization on lower extremities, nevertheless, we have chosen the arteries based on our previous study [3], in which arterial allografts had better patency results, than veins.

## Conclusions

Our results revealed that if arterial allografts were cryopreserved and stored for a period of up to 6 months, their hemostatic potential remained within the variability range of the fresh native arterial wall. In addition, both the fibrin generation and the platelet adhesiveness of the cryopreserved artery allografts declined in the course of time, minimizing the risk for thrombotic occlusion of the implanted grafts. Thus, the applied cryopreservation method demonstrated a twofold clinical benefit: retained hemostatic and reduced thrombogenic potential of the grafts. The only transient prothrombotic change was observed in the media of the cryopreserved artery allografts, where the platelet deposition exceeded that of the fresh native grafts in the initial twelve weeks after cryopreservation. If the grafts are to be used in this early storage period, antiplatelet therapy may be justified to prevent thrombotic occlusion.

The definite favorable trend of changes in the thrombogenicity of the grafts during the six-month monitoring period of the current study justifies a longer-term investigation for optimization of the storage period of cryopreserved arterial allografts in terms of thrombogenicity.

## Supporting information

S1 File Detailed statistics. (PDF)

S1 Data Original datasets. (ZIP)

## References

[1] Müller-Schweinitzer E. Cryopreservation of vascular tissues. Organogenesis. 2009; 5: 97–104. doi: 10.4161/org.5.3.9495 20046671 PMC2781088

[2] Wang CC, Lopez-Valdes S, Lin TL, Yap A, Yong CC, Li WF, et al. Outcomes of long storage times for cryopreserved vascular grafts in outflow reconstruction in living donor liver transplantation. Liver Transplant. 2014; 20: 173–181. doi: 10.1002/lt.23785 24382821

[3] Nagy Z, Oláh Z, Kókai J, Molnár AB, Laczkó Á, Szabó GV, et al. Homograftok szerepe az alsó végtagi érrekonstrukciókban. Magy Seb. 2017; 70: 5–12. doi: 10.1556/1046.70.2017.1.1 28294663

[4] Minga Lowampa E, Holemans C, Stiennon L, Van Damme H, Defraigne JO. Late fate of cryopreserved arterial allografts. Eur J Vasc Endovasc Surg. 2016; 52: 696–702. doi: 10.1016/j.ejvs.2016.08.005 27614553

[5] Tatar AR, Derycke L, Cochennec F, Jaziri A, Desgranges P, Touma J. Unmet needs in cryopreserved arterial allograft implantation for peripheral vascular graft infections. Eur J Vasc Endovasc Surg. 2020; 60: 788–789. doi: 10.1016/j.ejvs.2020.08.001 32912761

[6] Lejay A, Delay C, Girsowicz E, Chenesseau B, Bonnin E, Ghariani MZ, et al. Cryopreserved cadaveric arterial allograft for arterial reconstruction in patients with prosthetic infection. Eur J Vasc Endovasc Surg. 2017; 54: 636–644. doi: 10.1016/j.ejvs.2017.07.016 28890027

[7] Hartranft CA, Noland S, Kulwicki A, Holden CR, Hartranft T. Cryopreserved saphenous vein graft in infrainguinal bypass. J Vasc Surg. 2014; 60: 1291–1296. doi: 10.1016/j.jvs.2014.05.092 24997807

[8] Guevara-Noriega KA, Lucar-Lopez GA, Pomar JL. Cryopreserved allografts for treatment of chronic limb-threatening ischemia in patients without autologous saphenous veins. Ann Vasc Surg. 2019; 60: 379–387. doi: 10.1016/j.avsg.2019.03.018 31200034

[9] Singh K, Juneja A, Bajaj T, Voto C, Schor J, Zia S, et al. Single tertiary care center outcomes after lower extremity cadaveric vein bypass for limb salvage. Vasc Endovascular Surg. 2020; 54: 430–435. doi: 10.1177/1538574420925586 32489155

[10] Noel AA, Gloviczki P, Cherry KJ, Safi H, Goldstone J, Morasch MD, et al. Abdominal aortic reconstruction in infected fields: early results of the United States cryopreserved aortic allograft registry. J Vasc Surg. 2002; 35: 847–852. doi: 10.1067/mva.2002.123755 12021697

[11] Farber A, Major K, Wagner WH, Cohen JL, Cossman D V., Lauterbach SR, et al. Cryopreserved saphenous vein allografts in infrainguinal revascularization: analysis of 240 grafts. J Vasc Surg. 2003; 38: 15–21. doi: 10.1016/s0741-5214(03)00330-6 12844083

[12] Wagner DD, Burger PC. Platelets in Inflammation and Thrombosis. Arterioscler Thromb Vasc Biol. 2003; 23: 2131–2137. doi: 10.1161/01.ATV.0000095974.95122.EC 14500287

[13] Hungarian National Blood Transfusion Service. Legislation of organ and tissue transplantation. 2020. Available from: https://www.ovsz.hu/en/organ-coordination-office/legislation; https://www.ovsz.hu/hu/oco/jogszabalyok.

[14] European Comission. EudraLex—The rules governing medicinal products in the european union: Volume 4—EU guidelines to good manufacturing practice medicinal products for human and veterinary use; Annex 1—Manufacture of sterile medicinal products (corrected version). 2008. Available from: https://www.gmp-compliance.org/files/guidemgr/annex%2001[2008].pdf.

[15] Komorowicz E, McBane RD, Fass DN. Physical and enzymatic perturbation of the architecture of the tunica media destroys its inherent thromboresistance. Thromb Haemost. 2002; 88: 827–833. doi: 10.1055/s-0037-1613310 12428102

[16] Wohner N, Keresztes Z, Sótonyi P, Szabó L, Komorowicz E, Machovich R, et al. Neutrophil granulocyte-dependent proteolysis enhances platelet adhesion to the arterial wall under high-shear flow. J Thromb Haemost. 2010; 8: 1624–1631. doi: 10.1111/j.1538-7836.2010.03890.x 20412433 PMC2905611

[17] Weiss HJ, Turitto VT, Baumgartnet HR. Role of shear rate and platelets in promoting fibrin formation on rabbit subendothelium. Studies utilizing patients with quantitative and qualitative platelet defects. J Clin Invest. 1986; 78: 1072–1082. doi: 10.1172/JCI112663 3760183 PMC423764

[18] Nikolova N, Chai S, Ivanova SD, Kolev K, Tenekedjiev K. Bootstrap Kuiper testing of the identity of 1D continuous distributions using fuzzy samples. Int J Comput Intell Syst. 2015; 8: 63–75. doi: 10.1080/18756891.2015.1129592

[19] Nikolova N, Panayotov P, Panayotova D, Ivanova S, Tenekedjiev K. Using fuzzy sets in surgical treatment selection and homogenizing stratification of patients with significant chronic ischemic mitral regurgitation. Int J Comput Intell Syst. 2019; 12: 1075–1090. doi: 10.2991/ijcis.d.190923.002

[20] Farkas ÁZ, Farkas VJ, Gubucz I, Szabó L, Bálint K, Tenekedjiev K, et al. Neutrophil extracellular traps in thrombi retrieved during interventional treatment of ischemic arterial diseases. Thromb Res. 2019; 175: 46–52. doi: 10.1016/j.thromres.2019.01.006 30703701

[21] Hisada Y, Grover SP, Maqsood A, Houston R, Ay C, Noubouossie DF, et al. Neutrophils and neutrophil extracellular traps enhance venous thrombosis in mice bearing human pancreatic tumors. Haematologica. 2020; 105: 218–225. doi: 10.3324/haematol.2019.217083 31048354 PMC6939515

[22] Nikolova N, Toneva D, Tsonev Y, Burgess B, Tenekedjiev K. Novel methods to construct empirical CDF for continuous random variables using censor data. 2020 IEEE 10th Int Conf Intell Syst IS 2020—Proc. 2020: 61–68. doi: 10.1109/IS48319.2020.9199954

[23] Nikolova N, Mihaylova N, Tenekedjiev K. Bootstrap tests for mean value differences over fuzzy samples. IFAC-PapersOnLine. 2015; 48: 7–14. doi: 10.1016/j.ifacol.2015.12.048

[24] Tenekedjiev K, Nikolova N, Rodriguez RM, Hirota K. Bootstrap testing of central tendency nullity over paired fuzzy samples. Int J Fuzzy Syst. 2021; Forthcoming.

[25] Nikolova N, Rodriguez RM, Symes M, Toneva D, Kolev K, Tenekedjiev K. Outlier detection algorithms over fuzzy data with weighted least squares. Int J Fuzzy Syst. 2021; doi: 10.1007/s40815-020-01049-8

[26] Turtiainen J, Saimanen E, Partio T, Kärkkäinen J, Kiviniemi V, Mäkinen K, et al. Surgical wound infections after vascular surgery: prospective multicenter observational study. Scand J Surg. 2010; 99: 167–172. doi: 10.1177/145749691009900312 21044935

[27] Turtiainen J, Hakala T. Surgical wound infections after peripheral vascular surgery. Scand J Surg. 2014; 103: 226–231. doi: 10.1177/1457496913514384 24737857

[28] Chakfé N, Diener H, Lejay A, Assadian O, Berard X, Caillon J, et al. Editor’s Choice–European Society for Vascular Surgery (ESVS) 2020 clinical practice guidelines on the management of vascular graft and endograft infections. Eur J Vasc Endovasc Surg. 2020; 59: 339–384. doi: 10.1016/j.ejvs.2019.10.016 32035742

[29] Wilson WR, Bower TC, Creager MA, Amin-Hanjani S, O’Gara PT, Lockhart PB, et al. Vascular graft infections, mycotic aneurysms, and endovascular infections: a scientific statement from the American Heart Association. Circulation. 2016; 134: e412–460. doi: 10.1161/CIR.0000000000000457 27737955

[30] Bíró G, Szeberin Z, Nemes A, Acsády G. Cryopreserved homograft and autologous deep vein replacement for infrarenal aorto and iliaco-femoral graft infection: Early and late results. J Cardiovasc Surg (Torino). 2011; 52: 169–176. 21464818

[31] McPhee JT, Barshes NR, Ozaki CK, Nguyen LL, Belkin M. Optimal conduit choice in the absence of single-segment great saphenous vein for below-knee popliteal bypass. J Vasc Surg. 2012; 55: 1008–1014. doi: 10.1016/j.jvs.2011.11.042 22365176

[32] Conte MS, Bradbury AW, Kolh P, White J V., Dick F, Fitridge R, et al. Global vascular guidelines on the management of chronic limb-threatening ischemia. Eur J Vasc Endovasc Surg. 2019; 58: S1–S109.e33. doi: 10.1016/j.ejvs.2019.05.006 31182334 PMC8369495

[33] Hruby J, Spunda R, Mericka P, Mlcek M, Pecha O, Splith K, et al. Influence of the new standardized clinical cryopreservation/slow thawing protocol on immunogenicity of arterial allografts in rats. PLoS One. 2020; 15: 1–12. doi: 10.1371/journal.pone.0230234 32155226 PMC7064217

[34] Špaček M, Měřička P, Janoušek L, Štádler P, Adamec M, Vlachovský R, et al. Current vascular allograft procurement, cryopreservation and transplantation techniques in the Czech Republic. Adv Clin Exp Med. 2019; 28: 529–534. doi: 10.17219/acem/90037 30684317

[35] Gökmen BG, Özcan O, Taslak H, Ipar N, Tunali-Akbay T. Effect of freezing time on tissue factor activity and macronutrients of human milk. Protein J. 2020; 39: 591–597. doi: 10.1007/s10930-020-09916-x 32989648

[36] Miller VM, Bergman RT, Gloviczki P, Brockbank KGM. Cryopreserved venous allografts: Effects of immunosuppression and antiplatelet therapy on patency and function. J Vasc Surg. 1993; 18: 216–226. doi: 10.1016/0741-5214(93)90601-H 8350430

[37] Wilcox JN, Smith KM, Schwartz SM, Gordon D. Localization of tissue factor in the normal vessel wall and in the atherosclerotic plaque. Proc Natl Acad Sci U S A. 1989; 86: 2839–2843. doi: 10.1073/pnas.86.8.2839 2704749 PMC287014

[38] Toschi V, Gallo R, Lettino M, Fallon JT, David Gertz S, Fernández-Ortiz A, et al. Tissue factor modulates the thrombogenicity of human atherosclerotic plaques. Circulation. 1997; 95: 594–599. doi: 10.1161/01.cir.95.3.594 9024145

[39] Vlodavsky I, Eldor A, HyAm E, Atzom R, Fuks Z. Platelet interaction with the extracellular matrix produced by cultured endothelial cells: a model to study the thrombogenicity of isolated subendothelial basal lamina. Thromb Res. 1982; 28: 179–191. doi: 10.1016/0049-3848(82)90260-2 7179215

[40] Fauvel-Lafève F. Microfibrils from the arterial subendothelium. Int Rev Cytol. 1999; 188: 1–40. doi: 10.1016/s0074-7696(08)61564-8 10208009

