# Supplementary material for: Cryopreservation moderates the thrombogenicity of arterial allografts during storage
Source: PLoS One. 2021 Jul 22;16(7):e0255114. doi: 10.1371/journal.pone.0255114 (PMC8297765; doi:10.1371/journal.pone.0255114)
Supplement: S1 File — (PDF) [file pone.0255114.s001.pdf]

Supporting information

Cryopreservation moderates the thrombogenicity of arterial allografts during storage

László Hidi, Erzsébet Komorowicz, Gergely Kovács, Zoltán Szeberin, Dávid Garbaisz, Natalia Nikolova, Kiril Tenekedjiev, László Szabó, Krasimir Kolev, Péter Sótonyi

Detailed statistical approach

I. Datasets

We conducted experiments over arteries from 11 donors (r=1, 2,…,11). Five arterial samples (j=1, 2,…,5) were taken from each artery, at different time points during the storage after the addition of the storage medium. The first arterial sample (j=1) was not treated with storage medium, whereas the arterial samples from two to five (j=2,3,4,5) were kept in the storage medium less than 1 day for j=2, one week for j=3, 12 weeks for j=4, and 24 weeks for j=5. Each of the 55 arterial samples, $S_{r,j}$, were perfused with blood before being studied with the immunofluorescent method described in the "Methods" section and data from different regions of interest in the micrographs were analyzed. The regions were divided into six logical groups corresponding to the three layers of the artery (Adventitia for L=1, Media for L=2, and Intima for L=3) and to the measured thrombogenic factor (the percentage of area covered by fibrin for T=1 and the percentage of area covered by platelets for T=2). We use the designation $m_{r,j}^{L,T}$ for the count of regions in the Lth layer from the rth donor at the jth treating time where the Tth thrombogenic factor was measured. If the kth measurement of this type is $y_{k,r,j}^{L,T}$ (k=1,2,…,$m_{r,j}^{L,T}$) we can form five data samples for each combination of L and T at any time point. To achieve parity of the arterial samples from different donors we associated the observation $y_{k,r,j}^{L,T}$ with a degree of membership $\left(\mu_{k,r,j}^{L,T} = 1/m_{r,j}^{L,T}\right)$ to the set $\left(S_j^{L,T}\right)$ of all measurements for the Tth thrombogenic factor in the Lth layer at the jth treating time (such approach was successfully applied in [**Error! Reference source not found.**,**Error! Reference source not found.**,**Error! Reference source not found.**]). The fuzzy sample $\chi_j^{L,T}$ is from the jth treating time and contains only the available information for $S_j^{L,T}$ (see [**Error! Reference source not found.**] for

interpretations of the term fuzzy sample). The $\chi_j^{L,T}$ contains couples of "measured values and degree of membership" with count $n_j^{L,T} = \sum_{t=1}^{11} m_{r,j}^{L,T}$:

$$\chi_j^{L,T} = \left\{ \left(x_{1,j}^{L,T}; \mu_{1,j}^{L,T} \right), \left(x_{2,j}^{L,T}; \mu_{2,j}^{L,T} \right), \ldots, \left(x_{n_j^{L,T},j}^{L,T}; \mu_{n_j^{L,T},j}^{L,T} \right) \right\} \quad \text{, for } j = 1,2,\ldots,5 \tag{1}$$

The first $m_{1,j}^{L,T}$ couples in the fuzzy sample $\chi_j^{L,T}$ are the measurements for the first donor $\left(y_{k,1,j}^{L,T}; \mu_{k,1,j}^{L,T} \right)$, for $k = 1,2,\ldots,m_{1,j}^{L,T}$. The second $m_{2,j}^{L,T}$ couples in the fuzzy sample $\chi_j^{L,T}$ are the measurements for the second donor $\left(y_{k,2,j}^{L,T}; \mu_{k,2,j}^{L,T} \right)$, for $k = 1,2,\ldots,m_{2,j}^{L,T}$, and so on till the last $m_{11,j}^{L,T}$ couples which are the measurements for the eleventh donor $\left(y_{k,11,j}^{L,T}; \mu_{k,11,j}^{L,T} \right)$, for $k = 1,2,\ldots,m_{11,j}^{L,T}$ .The raw data $y_{k,r,j}^{L,T}$ (k=1,2,…, $m_{r,j}^{L,T}$, r=1,2,…,11, j=1, 2,…,5, L=1,2,3, and T=1,2) are presented with the same designations as Supplementary data.

In this study we focused on identifying the influence of the storage time on the thrombogenic factor abundance in different layers. The statistical analysis was performed six times, separately for each combination of L and T. Because of the identity of the approach for all L-T combinations, in this section we can simplify the notation by omitting the L and T indices. So, each of the five fuzzy samples, χ_j , contains n_j fuzzy variates of the random variable X_j "percentage of area covered by the thrombogenic factor at the jth storage time":

$$\chi_j = \left\{ \left(x_{1,j}; \mu_{1,j} \right), \left(x_{2,j}; \mu_{2,j} \right), \ldots, \left(x_{n_j,j}; \mu_{n_j,j} \right) \right\} \quad \text{, for } j = 1,2,\ldots,5 \tag{2}$$

The five fuzzy sample (2) were sorted by renumbering the couples in χ_j so that X_j variates are in ascending order:

$$\chi_j^{sort}=\left\{\left(x_{1,j}^{sort};\mu_{1,j}^{sort}\right),\left(x_{2,j}^{sort};\mu_{2,j}^{sort}\right),\ldots,\left(x_{n_j,j}^{sort};\mu_{n_j,j}^{sort}\right)\right\} \quad \text{, for } j=1,2,\ldots,5$$
$$\text{where } x_{1,j}^{sort}\le x_{2,j}^{sort}\le\cdots\le x_{n_j,j}^{sort} \tag{3}$$

The count of the observations, n_j, in the fuzzy samples (2) depends on the layer (L), on the thrombogenic factor (T) and on treating time (j), which is summarised in Table 1.

Table 1: The size of the fuzzy sample n_j, for the separate time points (j), thrombogenic factor (T) and layer (L)

L	**1**	**1**	**2**	**2**	**3**	**3**
T	**1**	**2**	**1**	**2**	**1**	**2**
***j*=1**	149	158	153	160	113	114
***j*=2**	160	162	160	162	101	101
***j*=3**	150	155	165	168	105	106
***j*=4**	212	211	111	200	124	125
***j*=5**	176	176	219	225	139	139

II. Distribution Functions and α-quantiles

Any approximation of the cumulative distribution function (CDF) of a random variable based on a random sample of variates of the *random variable* can be denoted as sample CDF (SCDF). The data in the fuzzy sample χ_j was used to construct three different forms of the SCDF.

The best-known form of SCDF is the empirical CDF (ECDF). The latter disregards the membership degrees in the fuzzy sample (so it can be constructed if a crisp sample is given). ECDF is a step function which jumps with $1/n_j$ at any variate value in the sample:

$$F_j\left(x\right)=\frac{\sum\limits_{\substack{i=1\\x_{i,j}<x}}^{n_j}1}{n_j} \quad \text{, for } j=1,2,\ldots,5 \tag{4}$$

The second form of SCDF is the fuzzy ECDF (FECDF), which is a generalization of (4). FECDF is a step function, which jumps with $\mu_{i,j}$ at any variate value $x_{i,j}$ in the fuzzy sample:

$$F_j\left(x\right)=\frac{\sum\limits_{\substack{i=1\\x_{i,j}<x}}^{n_j}\mu_{i,j}}{\sum\limits_{i=1}^{n_j}\mu_{i,j}}\quad\text{, for } j=1,2,\ldots,5 \tag{5}$$

Both ECDF and FECDF do not have inverse and are not suitable to identify α-quantiles, because all conventional procedures usually identify those quantiles as one of the variates in the sample. Those SCDFs are useful in the Bootstrap procedures described below.

The preferred form of SCDF is a fuzzy version of the invertible CDF estimator with maximum count of nodes (FICDFmax) which is strictly increasing in the domain [$x_{beg,j}$, $x_{end,j}$]. That method constructs $F_j(x)$, as a linear interpolation on a set of R_j nodes:

$$NDS_j=\left\{\left(z_{k,j},F_{k,j}\right)\mid k=1,2,\ldots,R_j\right\}\text{ , for } j=1,2,\ldots,5 \tag{6}$$

The count of the nodes, R_j, and the values of their strictly increasing abscissas ($z_{k,j}$) are determined according to [**Error! Reference source not found.**]. The lower and upper bound of the domain, where $F_j(x)$ is strictly increasing, have been naturally selected as:

$$0\%=x_{beg,j}=z_{1,j}=<z_{2,j}<z_{2,j}<\ldots<z_{R_j-1,j}<z_{R_j,j}=x_{end,j}=100\%\text{ , for } j=1,2,\ldots,5 \tag{7}$$

The values of the nodes' strictly increasing ordinates ($F_{k,j}$ for $k=1,2,\ldots,R_j$) were calculated by the Universal SCDF Estimator (introduced in [**Error! Reference source not found.**]) in a modified form to account for the fuzzy character of the sample χ_j and for the absence of right-censored data:

$$F_{k,j}=\frac{\sum\limits_{\substack{i=1\\x_{i,j}<z_{k,j}}}^{n_j}\mu_{i,j}+\sum\limits_{\substack{i=1\\x_{i,j}\le z_{k,j}}}^{n_j}\mu_{i,j}}{2\sum\limits_{i=1}^{n_j}\mu_{i,j}}\quad\text{, for } k=1,2,\ldots,R_j \text{ and } j=1,2,\ldots,5 \tag{8}$$

Because the nodes in NDS_j have strictly increasing abscissas, the FICDFmax function, $F_j(x)$, can be constructed as:

$$\text{Domain: } x \in (-\infty, \infty)$$

$$F_j(x) = \begin{cases} 0 & \text{if } x \in (-\infty, z_{1,j}) \\ F_{k,j} & \text{if } x = z_{k,j} \quad \text{,for } k = 1,2,\ldots,R_j \\ F_{k,j} + \dfrac{F_{k+1,j} - F_{k,j}}{z_{k+1,j} - z_{k,j}}(x - z_{k,j}) & \text{if } x \in (z_{k,j}, z_{k+1,j}) \quad \text{,for } k = 1,2,\ldots,R_j - 1 \\ 1 & \text{if } x \in (z_{R_j,j}, \infty) \end{cases} \tag{9}$$

Because the nodes in NDS_j have strictly increasing ordinates, the inverse FICDFmax function, $F_j^{-1}(\alpha)$, can also be constructed in the domain [0,1]:

$$\text{Domain: } \alpha \in [0,1]$$

$$F_j^{-1}(\alpha) = \begin{cases} z_{k,j} & \text{if } \alpha = F_{k,j} \quad \text{,for } k = 1,2,\ldots,R_j \\ z_{k,j} + \dfrac{z_{k+1,j} - z_{k,j}}{F_{k+1,j} - F_{k,j}}(\alpha - F_{k,j}) & \text{if } \alpha \in (F_{k,j}, F_{k+1,j}) \quad \text{,for } k = 1,2,\ldots,R_j - 1 \end{cases} \tag{10}$$

Using the inverse FICDFmax function (12), any α-quantile describing the random variable X_j can be implicitly estimated:

$$q_{\alpha,j}^{imp} = F_j^{-1}(\alpha) \quad \text{, for } \alpha \in [0,1] \text{ and } j = 1,2,\ldots,5 \tag{11}$$

The implicit estimates of the median, the lower quartile, and the upper quartile of the random variable X_j are $q_{0.5,j}^{imp}$, $q_{0.25,j}^{imp}$, and $q_{0.75,j}^{imp}$, respectively.

Alternatively, the α-quantiles describing the random variable. X_j can be explicitly estimated without constructing an inverse SCDF function. Instead, the explicit method calculates $q_{\alpha,j}^{exp}$ as a linear interpolation on the set of $(2n_j - 1)$ nodes:

$$qNDS_j = \left\{ (\alpha_{k,j}, q_{k,j}) \mid k = 1,2,\ldots,2n_j - 1 \right\} \text{ , for } j = 1,2,\ldots,5 \tag{12}$$

The values of the increasing nodes' ordinates ($q_{k,j}$) are:

$$q_{k,j}=\begin{cases} x^{sort}_{(1+k)/2,j} & \text{when } k=1,3,\ldots,2n_j-1 \\ \left(x^{sort}_{k/2,j}+x^{sort}_{k/2+1,j}\right)/2 & \text{when } k=2,4,\ldots,2n_j-2 \end{cases}\quad \text{, for } j=1,2,\ldots,5 \tag{13}$$

The values of the strictly increasing nodes' abscissas ($\alpha_{k,j}$) in $qNDS_j$ are:

$$\alpha_{k,j}=\begin{cases} \left[\sum_{i=1}^{(k+1)/2}\left(\mu^{sort}_{i,j}\right)-0.5\mu^{sort}_{(k+1)/2,j}\right]/\sum_{i=1}^{n_j}\left(\mu^{sort}_{i,j}\right) & \text{when } k=1,3,\ldots,2n_j-1 \\ \sum_{i=1}^{k/2}\left(\mu^{sort}_{i,j}\right)/\sum_{i=1}^{n_j}\left(\mu^{sort}_{i,j}\right) & \text{when } k=2,4,\ldots,2n_j-2 \end{cases}\quad \text{, for } j=1,2,\ldots,5 \tag{14}$$

Because the nodes in $qNDS_j$ have strictly increasing abscissas, the explicit α-quantile describing the random variable X_j, $q^{exp}_{\alpha,j}$, can be calculated for any α in the domain $\left[\alpha_{1,j},\alpha_{n_j,j}\right]$:

$$\text{Domain: } \alpha\in\left[\mu^{sort}_{1,j}\Big/2\sum_{i=1}^{n_j}\mu^{sort}_{j,j},1-\mu^{sort}_{n_j,j}\Big/2\sum_{i=1}^{n_j}\mu^{sort}_{j,j}\right]$$

$$q^{exp}_{\alpha,j}=\begin{cases} x^{sort}_{k,j} & \text{if } \alpha=\alpha_{k,j} & \text{,for } k=1,2,\ldots,n_j \\ x^{sort}_{k,j}+\dfrac{x^{sort}_{k+1,j}-x^{sort}_{k,j}}{\alpha_{k+1,j}-\alpha_{k,j}}\left(\alpha-\alpha_{k,j}\right) & \text{if } \alpha\in\left(\alpha_{k,j},\alpha_{k+1,j}\right) & \text{,for } k=1,2,\ldots,n_j-1 \end{cases} \tag{15}$$

The explicit estimates of the median, the lower quartile, and the upper quartile of the random variable X_j are $q^{exp}_{0.5,j}$, $q^{exp}_{0.25,j}$, and $q^{exp}_{0.75,j}$, respectively.

If there are no ties in the variates of χ^{sort}_j, then $q^{imp}_{\alpha,j}=q^{exp}_{\alpha,j}$ for $\alpha\in\left[\alpha_{1,j},\alpha_{n_j,j}\right]$.

III. Significance of Qualitative Differences at Measured Times

We investigated qualitatively the influence of the storage medium treating time over the kth quartile of the thrombogenic factor abundance (i.e., for the lower quartile k=1, for the median k= 2, and for

the upper quartile k=3). We compared the kth quantiles of the five random variables $X_1, X_2,\ldots,X_5$ on the three quartiles (for k=1,2,3) for each of the six combinations of layer and thrombogenic factor (L=1,2,3 and T=1,2).

The $P_{k,j}^{true}$ (for j=1,2,…,5) designation was used for the kth quartile of the random variable. X_j "percentage of area covered with the thrombogenic factor at the jth treating time". As $P_{k,j}^{true}$ is a descriptor of the X_j distribution in the general population, it is an unknown non-random value. Using the fuzzy samples $\chi_1, \chi_2, \chi_3, \chi_4$, and χ_5 we can derive an estimate, $E_{k,j} = q_{0.25k,j}^{exp}$ for $P_{k,j}^{true}$. The estimate $E_{k,j}$ is a random variate because it depends on the random data in χ_j. We can order the five estimates $E_{k,j}$ (for j=1,2,…,5) in descending order that contains 10 different comparisons $E_{k,jb} > E_{k,js}$ where $jb \neq js$. We tested the null hypothesis $H_{0,k}^{jb,js}$ (that the kth quartiles of X_{jb} and X_{js} are the equal) against the alternative hypothesis H_1 (that the kth quartile of X_{jb} is greater than the kth quartile X_{js}):

$$H_{0,k}^{jb,js}: P_{k,jb}^{true} = P_{k,js}^{true} \quad \text{against} \quad H_1: P_{k,jb}^{true} > P_{k,js}^{true} \tag{16}$$

Four different Bootstrap one-tailed tests were used to analyze the kth quartile differences over the fuzzy samples χ_{jb} and χ_{js}, all of which solve the formulated problem by using the test statistics $\Delta_{jb-js,r}^{k-quartile}$ (the difference of the kth quartile estimates from the two fuzzy samples):

$$\Delta_{jb-js,r}^{k-quartile} = E_{k,jb} - E_{k,jb} = q_{0.25k,jb}^{exp} - q_{0.25k,js}^{exp} \tag{17}$$

The algorithm to estimate the p-values of the four Bootstrap one-tailed tests of (17) is described in [6]. The only modification was the substitution of the fuzzy mean value formula with the procedure (11)-(14) for explicit (0.25k)-quantile estimation from a fuzzy sample. The four tests differ: a) in the type of the generated synthetic fuzzy sample – the generated synthetic fuzzy samples can be either "quasi-equal information samples" (the sum of membership degree of any synthetic sample is very similar to the sum in the original sample), or "equal-size samples" (the count of observations of any synthetic

sample equals the count in the original sample); b) in the sample distribution used in the synthetic fuzzy sample generation – the sample distribution for the synthetic fuzzy sample generation can either be ECDF (the observations in the original fuzzy sample have equal chance to be drawn with replacement into the synthetic fuzzy sample), or FECDF (the observations in the original fuzzy sample have a chance proportional to their degree of membership to be drawn with replacement into the synthetic fuzzy sample). Each of the four p-values is compared to the predetermined significance level α (we use α =0.05) and the hypothesis $H_{0,k}^{jb,js}$ is rejected if at least two of those p-values are less than α. We use the designation $Cl_{hyp,k}$ for the cluster of the four fuzzy k^{th}-quartile one-sided Bootstrap tests. Although each of those tests can operate on its own, it is more informative to use their results as a cluster providing complementary information for the solution of problem (16). In that way, we can avoid making random significance claims due to an odd low p-value in a single hypothesis test. Instead, the significance claims are based on evidence that at least half of the tests in $Cl_{hyp,k}$ have identified significant difference in the population k^{th}-quartile values of the random variable X_{jb} and X_{js}. The adopted cluster approach to hypothesis testing is proposed and demonstrated in [7]. The performance of the $Cl_{hyp,k}$ cluster of four fuzzy bootstrap tests is compared with the results of a bootstrap test performed using the above described algorithm on modified crisp samples $\chi_{j,crisp}^{L,T}=\left\{\left(x_{1,j}^{L,T};1\right),\left(x_{2,j}^{L,T};1\right),\ldots,\left(x_{n_j^{L,T},j}^{L,T};1\right)\right\}$, for $j=1,2,\ldots,5$. The latter samples are derived as special cases of (1) where all degrees of membership are artificially set to unity $\left(\mu_{k,r,j,crisp}^{L,T}=1\right)$. When operating on the crisp samples $\chi_{j,crisp}^{L,T}$, all the fuzzy bootstrap tests in $Cl_{hyp,k}$ degenerate to a single crisp bootstrap test as shown in [6].

The problem (16) was solved for each of the ten couples (X_{jb}, X_{js}) which satisfy the conditions: 1) $jb \neq js$, 2) $E_{k,jb} > E_{k,js}$, 3) $jb \in \{1,2,3,4,5\}$, and 4) $js \in \{1,2,3,4,5\}$ (in the rare case when condition 2 cannot be met because $E_{k,jb}=E_{k,js}$ we considered additional condition $jb<js$). Generally, there was no

statistically significant order of the kth quartiles of the random variable X_j (for j=1,2,…,5). Instead, the ten statistical comparisons between $P_{k,1}^{true}$, $P_{k,2}^{true}$, $P_{k,3}^{true}$, $P_{k,4}^{true}$, and $P_{k,5}^{true}$ often formed a non-transitive relation.

IV. Significance of Quantitative Time Trends

We also investigated quantitively the influence of the storage medium treating time over the kth quartile of the thrombogenic factor concentration (i.e., for the lower quartile k=1, for the median k= 2, and for the upper quartile k=3). The information in χ_2, χ_3, χ_4, and χ_5 was utilised to identify the trends for the median (Q_2), the lower quartile (Q_1), and the upper quartile (Q_3) of the thrombogenic factor abundance. For each of the three quartiles we constructed a linear regression with the time as independent variable:

$$Q_k = b_{k,0} + b_{k,1}t + \varepsilon_k \quad \text{, for } k = 1,2,3 \tag{18}$$

The kth regression (18) was trained on a fuzzy sample C_k containing four triplets in the form (time, quartile, degree of membership):

$$C_k = \left\{ \left(t_i, Q_{k,i}, \mu_{k,i} \right) | \, i = 1,2,3,4 \right\} \quad \text{, for } k = 1,2,3 \tag{19}$$

In (19), the times in weeks are t_1=0, t_2=1, t_3=12, and t_4=24, whereas the quartiles in % are

$$Q_{k,i} = q_{0.25k,i+1}^{exp} \quad \text{,for } i = 1,2,3,4 \text{ and } k = 1,2,3 \tag{20}$$

The degrees of membership reflect the precision of the quartile values at each of the four time points:

$$\mu_{k,i} = 1 / \left(q_{0.25k+0.125,i+1}^{exp} - q_{0.25k-0.125,i+1}^{exp} \right)^2 \quad \text{,for } i = 1,2,3,4 \text{ and } k = 1,2,3 \tag{21}$$

The values of the regression coefficients $b_{k,0}$ and $b_{k,1}$ were estimated to minimise the weighted least square, WLS_k, of the residuals, $e_{k,i}$ (the difference between the measured and the predicted k^{th} quartile value at time t_i from the training set):

$$WLS_k\left(b_{k,1},b_{k,2}\right)=\sum_{i=1}^{4}\mu_{k,i}\left(e_{k,i}\right)^2=\sum_{i=1}^{4}\mu_{k,i}\left(Q_{k,i}-b_{k,1}-b_{k,2}t_i\right)^2 \tag{22}$$

The solution of the optimization problem (22), the goodness-of-fit measures of (18), and the p-value of the analytical t-test for the significance of the single regression slope $b_{k,1}$ were calculated using the analytical Algorithm 1 from [8]. However, the classical regression assumptions for this analytical solution hardly hold and the results of the *t*-test about the significance of the estimated slope are unreliable. On the other hand, the structure of the problem (4 samples from which we derive the 4 regressand values and their precision) is suitable for fuzzy Bootstrap procedure to identify the distribution of the slope. We can use that distribution to find (100-100α)%-confidence interval for the slope (usually 95%-confidence interval). Even more important is that the identified distribution can provide the probabilities (P^- and P^{0+}) for the slope to be negative and non-negative (if the estimate $b_{k,1}$<0) or the probabilities (P^+ and P^{0-}) for the slope to be positive and non-positive (if the estimate $b_{k,1}$>0). Such probabilities are a much better tool to determine the significance of the identified slope sign, than the *p*-value of any statistical test, because at the latter we can calculate only the probability for being wrong if we reject the null hypothesis, but never the probability of being right when accepting the alternative one. We adopted the conservative policy to assume negativity/positivity of the slope only when the probability for the non-negativity/non-positivity) is less than the preselected significance level, α (usually α = 0.05). Four different fuzzy Bootstrap procedures were applied for fuzzy sample generation which differ: a) in the type of the generated synthetic fuzzy sample, and b) in the sample distribution used in the synthetic fuzzy sample generation (as explained in III). We utilized the following Bootstrap procedure to determine the significance of the slope:

1) Select quartile (k=1,2,3), layer (L=1,2,3), and thrombogenic factor (T=1,2)

2) From L and T form χ_2, χ_3, χ_4, χ_5 according to (1) and (2)

3) Select the count of the pseudo-realities, N (usually N=10000)

4) Select the significance level, α= 0.05

5) Repeat the following for each type of fuzzy Bootstrap procedure (FL=1,2,3,4)

A) Repeat for each pseudo-reality (r=1,2,…,N)

a) Generate four synthetic fuzzy samples, $\chi_2^{sym,r}$, $\chi_3^{sym,r}$, $\chi_4^{sym,r}$, $\chi_5^{sym,r}$, by drawing with replacement (according to FL) from the fuzzy samples χ_2, χ_3, χ_4, χ_5

b) Estimate the synthetic explicit quantile values in the Right-Hand-Sides of formulae (20) and (21) using the synthetic fuzzy samples and the procedure (12)-(15)

c) Form the synthetic training data $C_k^{sym,r} = \left\{\left(t_i, Q_k^{sym,r}, \mu_k^{sym,r}\right) \mid i = 1,2,3,4\right\}$

d) Solve the regression (18) using synthetic training data $C_k^{sym,r}$ using the analytical Algorithm 1 from [6] and identify $b_{1,k}^{sym,r}$

B) Form the synthetic crisp sample $B_k^{sym} = \left\{b_{1,k}^{sym,r} \mid r = 1,2,\ldots,N\right\}$

C) Find the (α/2)-quantile and the (1-α/2)-quantile of the $b_{1,k}$ distribution ($b_{1,k,\alpha/2}$ and $b_{1,k,1\text{-}\alpha/2}$) using the sample B_k^{sym} by applying procedure (12)-(15) , setting $\mu_{1,k}^{sym,r} = 1$, for $r = 1,2,\ldots,N$

D) Declare the (100-100α)%-confidence interval for the slope to be: $b_{1,k,\alpha/2} < b_{1,k} < b_{1,k,1\text{-}\alpha/2}$

E) If $b_{1,k}$<0, then:

a) set the direct probability $P^{+0} = \#\left(b_{1,k}^{sym,r} \geq 0\right) / N$

b) if $P^{+0} \leq \alpha$, declare the slope $b_{1,k}$ as significantly negative according to procedure FL

c) if $P^{+0} > \alpha$, declare the slope $b_{1,k}$ as insignificant according to procedure FL

F) If $b_{1,k}$>0, then:

a) set the direct probability $P^{-0} = \#\left(b_{1,k}^{sym,r} \leq 0\right) / N$

b) if $P^{-0} \leq \alpha$, declare the slope $b_{1,k}$ as significantly positive according to procedure *FL*

c) if $P^{-0} > \alpha$, declare the slope $b_{1,k}$ as insignificant according to procedure *FL*

6) If at least three of the four fuzzy Bootstrap procedures has declared the slope insignificant then declare the slope insignificant
7) If at least two of the four fuzzy Bootstrap procedures has declared the slope significant then:

A) If $b_{1,k}$<0, then declare the slope $b_{1,k}$ as significantly negative

B) If $b_{1,k}$>0, then declare the slope $b_{1,k}$ as significantly positive

We use the designation $Cl_{sign,k}$ for the cluster of the four fuzzy k^{th}-quartile Bootstrap procedures. Although each of those procedures can operate on its own, it is more informative to use their results as a cluster providing complementary information for significance of the slope sign. That is another example of successful application of the cluster approach.

References

1. Nikolova N, Panayotov P, Panayotova D, Ivanova S, Tenekedjiev K. Using fuzzy sets in surgical treatment selection and homogenizing stratification of patients with significant chronic ischemic mitral regurgitation. Int J Comput Intell Syst. 2019; 12: 1075–1090. doi: 10.2991/ijcis.d.190923.002.
2. Farkas ÁZ, Farkas VJ, Gubucz I, Szabó L, Bálint K, Tenekedjiev K, et al. Neutrophil extracellular traps in thrombi retrieved during interventional treatment of ischemic arterial diseases. Thromb Res. 2019; 175: 46–52. doi: 10.1016/j.thromres.2019.01.006.
3. Hisada Y, Grover SP, Maqsood A, Houston R, Ay C, Noubouossie DF, et al. Neutrophils and neutrophil extracellular traps enhance venous thrombosis in mice bearing human pancreatic tumors. Haematologica. 2020; 105: 218–225. doi: 10.3324/haematol.2019.217083.
4. Nikolova N, Chai S, Ivanova SD, Kolev K, Tenekedjiev K. Bootstrap Kuiper testing of the identity of 1D continuous distributions using fuzzy samples. Int J Comput Intell Syst. 2015; 8: 63–75. doi: 10.1080/18756891.2015.1129592.
5. Nikolova N, Toneva D, Tsonev Y, Burgess B, Tenekedjiev K. Novel methods to construct empirical CDF for continuous random variables using censor data. 2020 IEEE 10th Int Conf Intell Syst IS 2020 - Proc. 2020: 61–68. doi: 10.1109/IS48319.2020.9199954.
6. Nikolova N, Mihaylova N, Tenekedjiev K. Bootstrap tests for mean value differences over fuzzy samples. IFAC-PapersOnLine. 2015; 48: 7–14. doi: 10.1016/j.ifacol.2015.12.048.
7. Tenekedjiev K, Nikolova N, Rodriguez RM, Hirota K. Bootstrap testing of central tendency nullity over paired fuzzy samples. Int J Fuzzy Syst. 2021; Forthcoming.
8. Nikolova N, Rodriguez RM, Symes M, Toneva D, Kolev K, Tenekedjiev K, Outlier detection algorithms over fuzzy data with weighted least squares, International Journal of Fuzzy Systems, 2021; doi: 10.1007/s40815-020-01049-8
