# Supplementary material for: Cryopreservation moderates the thrombogenicity of arterial allografts during storage
Source: PLoS One. 2021 Jul 22;16(7):e0255114. doi: 10.1371/journal.pone.0255114 (PMC8297765; doi:10.1371/journal.pone.0255114)
Supplement: S1 Data — (ZIP) [file pone.0255114.s002.zip › Open_data_R1/Data_L1_T1.pdf]

Artery wall Layer: Adventitia (L=1)

Thrombogenic Factor: Fibrin (T=1)

Storage Medium Treatment: No treatment (j=1)

Count of Total Measurements: 149 (n_1=149)

measured values of $y^{1,1}_{k,r,1}$ which is

the percentage of area covered by Fibrin

in the region k of Adventitia layer from donor r

with No treatment by storage medium

	r=1	r=2	r=3	r=4	r=5	r=6	r=7	r=8	r=9	r=10	r=11
k=1	69.5	24.2	34.7	8.4	22.8	3.2	11.9	0.4	14.6	52.9	14.4
k=2	45.8	29.7	55.9	28.9	32.9	1.4	29.4	8.8	4.1	44.8	23.4
k=3	42.6	25.8	38.9	36.5	47.5	1.6	16.9	8.0	13.0	60.8	32.4
k=4	55.8	42.7	46.6	15.6	18.2	9.2	48.6	10.6	8.8	65.9	10.2
k=5	61.8	27.8	48.1	14.7	15.1	8.9	17.4	27.8	7.7	52.9	6.1
k=6	63.8	18.8	50.5	18.6	13.7	1.4	30.2	5.1	0.4	5.5	9.6
k=7	N/A	25.4	N/A	19.7	54.7	1.6	25.5	10.9	0.4	4.1	4.4
k=8	N/A	35.7	N/A	18.0	31.1	9.2	3.6	5.0	2.4	46.6	34.7
k=9	N/A	37.3	N/A	37.3	15.2	8.9	18.2	6.4	8.1	39.7	31.3

k=10	N/A	42.7	N/A	17.6	4.3	2.3	15.4	16.1	1.9	52.4	2.1
k=11	N/A	37.8	N/A	36.4	N/A	1.4	5.3	11.4	1.1	50.1	4.2
k=12	N/A	N/A	N/A	N/A	N/A	6.3	19.0	35.4	1.2	57.7	1.8
k=13	N/A	N/A	N/A	N/A	N/A	4.1	8.7	8.2	1.1	33.4	27.9
k=14	N/A	N/A	N/A	N/A	N/A	2.5	5.1	8.7	2.2	14.0	26.7
k=15	N/A	N/A	N/A	N/A	N/A	2.1	9.0	36.6	N/A	11.4	8.2
k=16	N/A	N/A	N/A	N/A	N/A	4.2	4.2	N/A	N/A	3.2	4.4
k=17	N/A	N/A	N/A	N/A	N/A	5.5	6.2	N/A	N/A	47.8	N/A
k=18	N/A	N/A	N/A	N/A	N/A	33.1	N/A	N/A	N/A	45.4	N/A
k=19	N/A	N/A	N/A	N/A	N/A	33.1	N/A	N/A	N/A	62.3	N/A
k=20	N/A	N/A	N/A	N/A	N/A	11.7	N/A	N/A	N/A	N/A	N/A
k=21	N/A	N/A	N/A	N/A	N/A	11.2	N/A	N/A	N/A	N/A	N/A
k=22	N/A	N/A	N/A	N/A	N/A	13.5	N/A	N/A	N/A	N/A	N/A
k=23	N/A	N/A	N/A	N/A	N/A	36.1	N/A	N/A	N/A	N/A	N/A
k=24	N/A	N/A	N/A	N/A	N/A	10.2	N/A	N/A	N/A	N/A	N/A

values of $m_{r,1}^{1,1}$ which is

the count of regions covered by Fibrin

in Adventitia layer from donor *r*

with No treatment by storage medium

r	1	2	3	4	5	6	7	8	9	10	11
count	6	11	6	11	10	24	17	15	14	19	16

Artery wall Layer: Adventitia (*L*=1)

Thrombogenic Factor: Fibrin (*T*=1)

Storage Medium Treatment: 0 weeks (*j*=2)

Count of Total Measurements: 160 (n_2=160)

measured values of $y_{k,r,2}^{1,1}$ which is

the percentage of area covered by Fibrin

in the region *k* of Adventitia layer from donor *r*

after 0 weeks treatment with storage medium

	r=1	*r*=2	*r*=3	*r*=4	*r*=5	*r*=6	*r*=7	*r*=8	*r*=9	*r*=10	*r*=11
k=1	50.3	36.2	25.6	18.6	25.2	9.5	23.2	5.4	13.7	24.7	7.3
k=2	74.8	25.9	19.4	42.4	23.9	1.9	3.8	21.4	9.7	9.7	16.0
k=3	48.4	27.9	37.4	19.6	24.0	0.6	10.7	11.0	1.8	7.7	0.2
k=4	47.8	58.1	26.2	40.9	54.9	1.5	10.2	13.2	2.3	8.7	5.7
k=5	29.6	27.0	12.3	14.3	2.4	1.2	5.1	8.8	3.2	52.4	0.7
k=6	28.8	N/A	36.9	24.2	37.3	2.6	19.0	20.2	10.0	10.7	0.2
k=7	72.8	N/A	40.7	11.7	40.0	47.3	11.4	20.0	13.8	10.4	0.6
k=8	63.9	N/A	30.5	30.4	36.0	54.2	12.0	22.6	7.2	9.0	20.9
k=9	42.6	N/A	23.2	31.3	N/A	13.7	3.6	18.0	5.2	8.9	3.7

k=10	62.0	N/A	18.6	22.3	N/A	15.9	28.5	0.0	7.5	6.9	20.0
k=11	N/A	N/A	18.5	11.6	N/A	8.3	17.2	0.1	4.2	1.3	5.6
k=12	N/A	N/A	N/A	21.5	N/A	20.2	22.8	9.9	8.0	0.6	29.6
k=13	N/A	N/A	N/A	11.1	N/A	13.8	1.1	9.8	8.3	10.9	4.7
k=14	N/A	N/A	N/A	8.3	N/A	23.6	1.1	17.7	25.5	12.8	1.8
k=15	N/A	N/A	N/A	10.0	N/A	11.0	15.9	N/A	18.0	23.0	9.5
k=16	N/A	N/A	N/A	16.7	N/A	16.4	19.9	N/A	1.8	17.6	14.1
k=17	N/A	N/A	N/A	15.4	N/A	3.2	36.3	N/A	2.4	6.5	11.5
k=18	N/A	N/A	N/A	34.0	N/A	14.8	N/A	N/A	N/A	2.7	N/A
k=19	N/A	N/A	N/A	N/A	N/A	11.0	N/A	N/A	N/A	10.5	N/A
k=20	N/A	N/A	N/A	N/A	N/A	0.8	N/A	N/A	N/A	N/A	N/A
k=21	N/A	N/A	N/A	N/A	N/A	18.7	N/A	N/A	N/A	N/A	N/A
k=22	N/A	N/A	N/A	N/A	N/A	55.6	N/A	N/A	N/A	N/A	N/A
k=23	N/A	N/A	N/A	N/A	N/A	0.7	N/A	N/A	N/A	N/A	N/A
k=24	N/A	N/A	N/A	N/A	N/A	34.3	N/A	N/A	N/A	N/A	N/A

values of $m_{r,2}^{1,1}$ which is

the count of regions covered by Fibrin

in Adventitia layer from donor *r*

after 0 weeks treatment with storage medium

r	1	2	3	4	5	6	7	8	9	10	11
count	10	5	11	18	8	24	17	14	17	19	17

Artery wall Layer: Adventitia (L=1)

Thrombogenic Factor: Fibrin (T=1)

Storage Medium Treatment: 1 week (j=3)

Count of Total Measurements: 150 (n_3=150)

measured values of $y_{k,r,3}^{1,1}$ which is

the percentage of area covered by Fibrin

in the region k of Adventitia layer from donor r

after 1 week treatment with storage medium

	r=1	r=2	r=3	r=4	r=5	r=6	r=7	r=8	r=9	r=10	r=11
k=1	33.7	49.3	14.1	11.8	63.9	25.8	30.1	5.0	2.9	44.5	14.1
k=2	19.8	32.3	15.1	30.7	51.3	56.3	18.9	26.9	0.8	39.4	13.8
k=3	34.3	26.6	14.1	57.8	51.3	75.1	9.8	17.2	1.5	48.7	13.3
k=4	26.5	27.0	16.7	64.5	45.0	29.7	11.0	14.5	1.5	33.4	12.3
k=5	21.9	34.6	15.8	55.7	59.5	42.2	16.5	17.7	1.0	49.5	9.3
k=6	9.0	35.4	27.1	20.4	63.7	18.9	4.3	5.8	2.7	14.5	1.7
k=7	44.1	9.6	11.6	11.1	23.0	9.8	20.3	17.9	0.2	20.1	4.3
k=8	31.9	26.9	18.6	32.6	64.7	24.4	12.4	7.0	0.3	50.8	8.1
k=9	48.5	53.4	17.3	21.3	84.3	16.2	12.5	12.1	0.8	11.0	9.8

k=10	54.4	53.4	32.6	4.7	25.4	17.9	21.1	4.8	2.8	15.1	5.7
k=11	17.9	30.3	25.3	14.8	46.7	11.8	29.3	27.8	N/A	0.5	11.7
k=12	33.3	33.8	22.6	7.2	64.1	20.3	N/A	9.8	N/A	0.4	12.9
k=13	34.9	27.5	N/A	17.8	40.2	19.4	N/A	46.7	N/A	N/A	2.5
k=14	19.2	45.0	N/A	15.5	33.9	32.5	N/A	11.7	N/A	N/A	0.9
k=15	N/A	N/A	N/A	15.8	N/A	34.6	N/A	11.1	N/A	N/A	N/A
k=16	N/A	N/A	N/A	N/A	N/A	30.7	N/A	33.5	N/A	N/A	N/A
k=17	N/A	N/A	N/A	N/A	N/A	N/A	N/A	20.1	N/A	N/A	N/A
k=18	N/A	N/A	N/A	N/A	N/A	N/A	N/A	24.2	N/A	N/A	N/A

values of $m_{r,3}^{1,1}$ which is

the count of regions covered by Fibrin

in Adventitia layer from donor r

after 1 week treatment with storage medium

r	1	2	3	4	5	6	7	8	9	10	11
count	14	14	12	15	14	16	11	18	10	12	14

Artery wall Layer: Adventitia (L=1)

Thrombogenic Factor: Fibrin (T=1)

Storage Medium Treatment: 12 weeks (j=4)

Count of Total Measurements: 212 (n_4=212)

measured values of $y_{k,r,4}^{1,1}$ which is

the percentage of area covered by Fibrin

in the region k of Adventitia layer from donor r

after 12 weeks treatment with storage medium

	r=1	r=2	r=3	r=4	r=5	r=6	r=7	r=8	r=9	r=10	r=11
k=1	11.6	10.2	9.7	12.4	14.2	14.2	3.3	31.3	4.4	10.6	10.0
k=2	3.8	6.2	8.9	12.6	21.1	11.9	4.2	33.3	6.0	13.7	46.5
k=3	1.0	8.9	9.6	34.9	1.9	17.7	40.3	39.1	1.8	11.6	22.4
k=4	0.8	11.5	11.4	24.1	14.8	7.0	48.0	31.0	0.1	8.0	5.5
k=5	8.6	18.6	7.6	42.0	12.6	17.8	42.7	8.7	1.7	15.3	8.5
k=6	7.6	10.4	4.4	26.9	26.4	7.3	30.1	23.7	0.1	19.0	10.4
k=7	1.4	5.0	8.1	8.7	23.3	18.9	36.2	25.0	0.5	24.3	12.2
k=8	0.9	2.5	8.1	6.7	21.5	25.9	8.0	5.1	0.1	11.0	7.9
k=9	4.1	16.2	19.8	24.8	30.9	39.4	12.0	6.7	0.3	12.5	15.1

k=10	25.7	11.9	7.0	11.0	11.5	17.9	2.0	12.2	4.6	10.6	13.1
k=11	16.6	14.7	4.6	10.8	4.9	11.5	3.2	10.0	3.5	6.5	5.7
k=12	0.5	5.9	22.0	19.7	5.1	22.7	5.9	9.4	3.3	2.7	1.3
k=13	1.2	19.0	31.4	16.4	29.1	16.7	6.2	5.2	13.4	16.5	1.8
k=14	1.1	17.8	13.2	2.5	15.7	N/A	4.2	14.4	1.7	10.7	0.3
k=15	6.5	20.8	N/A	31.6	54.0	N/A	10.3	1.4	6.1	N/A	3.1
k=16	37.4	16.2	N/A	29.9	0.9	N/A	9.2	8.4	1.6	N/A	16.6
k=17	5.2	18.8	N/A	N/A	14.7	N/A	32.1	4.1	0.9	N/A	15.3
k=18	13.2	19.4	N/A	N/A	15.2	N/A	38.0	4.4	3.0	N/A	13.7
k=19	13.0	18.1	N/A	N/A	N/A	N/A	N/A	17.3	0.9	N/A	4.8
k=20	22.4	24.0	N/A	N/A	N/A	N/A	N/A	3.3	7.5	N/A	6.8
k=21	18.3	25.9	N/A	N/A	N/A	N/A	N/A	3.7	10.6	N/A	1.7
k=22	20.8	29.0	N/A	N/A	N/A	N/A	N/A	7.4	7.1	N/A	12.2
k=23	15.6	13.7	N/A	N/A	N/A	N/A	N/A	N/A	N/A	N/A	13.1
k=24	3.4	11.4	N/A	N/A	N/A	N/A	N/A	N/A	N/A	N/A	3.3
k=25	N/A	1.0	N/A	N/A	N/A	N/A	N/A	N/A	N/A	N/A	3.8

k=26	N/A	N/A	N/A	N/A	N/A	N/A	N/A	N/A	N/A	N/A	8.4

values of $m_{r,4}^{1,1}$ which is

the count of regions covered by Fibrin

in Adventitia layer from donor r

after 12 weeks treatment with storage medium

r	1	2	3	4	5	6	7	8	9	10	11
count	24	25	14	16	18	13	18	22	22	14	26

Artery wall Layer: Adventitia (L=1)

Thrombogenic Factor: Fibrin (T=1)

Storage Medium Treatment: 24 weeks (j=5)

Count of Total Measurements: 176 (n_5=176)

measured values of $y_{k,r,5}^{1,1}$ which is

the percentage of area covered by Fibrin

in the region k of Adventitia layer from donor r

after 24 weeks treatment with storage medium

	r=1	r=2	r=3	r=4	r=5	r=6	r=7	r=8	r=9	r=10	r=11
k=1	24.4	10.2	15.8	14.1	57.4	9.4	28.3	34.5	0.7	19.3	3.6
k=2	19.6	4.5	9.3	11.4	71.1	8.0	24.6	21.4	0.1	26.0	0.6
k=3	11.6	3.5	23.2	15.6	21.0	23.7	35.1	22.4	0.5	13.8	3.6
k=4	1.0	1.1	17.1	6.4	31.5	10.0	33.0	33.0	0.1	8.0	2.7
k=5	21.3	6.3	3.7	4.7	12.2	14.6	34.8	27.2	1.3	4.5	5.4
k=6	17.6	14.1	18.3	3.9	16.7	10.2	23.9	18.8	9.9	17.6	3.4
k=7	34.8	22.1	15.8	16.6	14.4	10.5	27.1	24.5	11.1	24.3	4.5
k=8	19.8	23.3	9.9	17.3	32.4	19.4	17.1	7.7	1.7	9.2	35.6
k=9	13.5	23.0	19.5	13.7	41.4	14.4	11.4	6.8	3.1	2.0	17.6

k=10	19.3	4.9	6.8	5.9	42.0	29.7	17.1	34.7	4.4	2.8	23.0
k=11	N/A	N/A	N/A	22.2	0.3	11.0	13.6	21.8	0.7	1.2	6.4
k=12	N/A	N/A	N/A	N/A	22.3	20.4	11.5	23.3	23.1	4.6	1.6
k=13	N/A	N/A	N/A	N/A	20.8	42.0	25.2	23.9	16.7	0.8	4.1
k=14	N/A	N/A	N/A	N/A	0.9	12.7	11.4	31.4	7.0	0.5	17.3
k=15	N/A	N/A	N/A	N/A	6.0	N/A	14.5	34.6	20.7	10.2	10.6
k=16	N/A	N/A	N/A	N/A	79.7	N/A	33.7	66.0	8.3	31.5	6.7
k=17	N/A	N/A	N/A	N/A	19.1	N/A	19.0	N/A	9.2	9.4	7.0
k=18	N/A	N/A	N/A	N/A	11.3	N/A	28.0	N/A	6.0	6.5	5.6
k=19	N/A	N/A	N/A	N/A	N/A	N/A	19.0	N/A	3.1	4.8	7.3
k=20	N/A	N/A	N/A	N/A	N/A	N/A	14.5	N/A	N/A	3.4	15.7
k=21	N/A	N/A	N/A	N/A	N/A	N/A	23.9	N/A	N/A	N/A	22.6
k=22	N/A	N/A	N/A	N/A	N/A	N/A	22.1	N/A	N/A	N/A	3.4
k=23	N/A	N/A	N/A	N/A	N/A	N/A	19.2	N/A	N/A	N/A	N/A
k=24	N/A	N/A	N/A	N/A	N/A	N/A	27.2	N/A	N/A	N/A	N/A
k=25	N/A	N/A	N/A	N/A	N/A	N/A	28.7	N/A	N/A	N/A	N/A

k=26	N/A	N/A	N/A	N/A	N/A	N/A	29.4	N/A	N/A	N/A	N/A

values of $m_{r,5}^{1,1}$ which is

the count of regions covered by Fibrin

in Adventitia layer from donor r

after 24 weeks treatment with storage medium

r	1	2	3	4	5	6	7	8	9	10	11
count	10	10	10	11	18	14	26	16	19	20	22
