# Supplementary material for: Cryopreservation moderates the thrombogenicity of arterial allografts during storage
Source: PLoS One. 2021 Jul 22;16(7):e0255114. doi: 10.1371/journal.pone.0255114 (PMC8297765; doi:10.1371/journal.pone.0255114)
Supplement: S1 Data — (ZIP) [file pone.0255114.s002.zip › Open_data_R1/Data_L1_T2.pdf]

Artery wall Layer: Adventitia (L=1)

Thrombogenic Factor: Platelet (T=2)

Storage Medium Treatment: No treatment (j=1)

Count of Total Measurements: 158 (n_1=158)

measured values of $y_{k,r,1}^{1,2}$ which is

the percentage of area covered by Platelet

in the region k of Adventitia layer from donor r

with No treatment by storage medium

	r=1	r=2	r=3	r=4	r=5	r=6	r=7	r=8	r=9	r=10	r=11
k=1	17.8	25.8	11.3	41.8	6.5	9.4	29.6	7.4	17.9	17.9	9.1
k=2	25.3	36.2	25.7	25.8	14.2	9.5	22.8	14.5	12.2	23.0	6.4
k=3	15.9	29.1	19.6	39.5	16.4	3.1	22.7	25.5	14.4	18.8	8.0
k=4	12.0	32.5	14.2	27.5	23.6	8.5	14.4	31.1	13.9	13.8	4.0
k=5	20.3	22.7	11.5	27.1	25.4	11.6	16.2	28.1	16.2	10.5	5.8
k=6	29.0	12.2	20.6	18.5	23.3	9.5	28.1	19.5	16.2	19.4	6.7
k=7	N/A	24.7	N/A	15.1	31.8	3.1	18.4	12.1	15.9	21.0	3.7
k=8	N/A	20.9	N/A	11.5	25.8	8.5	24.9	13.6	14.2	32.8	16.1
k=9	N/A	35.4	N/A	34.8	32.6	11.6	19.8	17.3	16.6	28.6	12.7

k=10	N/A	15.8	N/A	21.1	30.2	7.2	35.5	15.1	18.5	19.3	10.3
k=11	N/A	20.7	N/A	23.6	N/A	4.5	42.7	24.6	16.5	35.8	8.0
k=12	N/A	N/A	N/A	32.6	N/A	9.3	32.3	36.1	23.2	14.4	12.8
k=13	N/A	N/A	N/A	2.4	N/A	7.2	25.3	17.8	4.0	11.7	15.8
k=14	N/A	N/A	N/A	12.6	N/A	7.3	21.8	26.3	10.9	12.7	16.9
k=15	N/A	N/A	N/A	19.2	N/A	10.9	14.1	21.7	N/A	31.4	15.6
k=16	N/A	N/A	N/A	10.4	N/A	9.1	15.0	37.6	N/A	23.0	11.1
k=17	N/A	N/A	N/A	16.0	N/A	4.3	21.1	N/A	N/A	13.9	N/A
k=18	N/A	N/A	N/A	18.1	N/A	16.1	N/A	N/A	N/A	19.6	N/A
k=19	N/A	N/A	N/A	15.3	N/A	8.9	N/A	N/A	N/A	19.6	N/A
k=20	N/A	N/A	N/A	N/A	N/A	7.6	N/A	N/A	N/A	N/A	N/A
k=21	N/A	N/A	N/A	N/A	N/A	9.0	N/A	N/A	N/A	N/A	N/A
k=22	N/A	N/A	N/A	N/A	N/A	8.2	N/A	N/A	N/A	N/A	N/A
k=23	N/A	N/A	N/A	N/A	N/A	14.6	N/A	N/A	N/A	N/A	N/A
k=24	N/A	N/A	N/A	N/A	N/A	11.7	N/A	N/A	N/A	N/A	N/A

values of $m_{r,1}^{1,2}$ which is

the count of regions covered by Platelet

in Adventitia layer from donor r

with No treatment by storage medium

r	1	2	3	4	5	6	7	8	9	10	11
count	6	11	6	19	10	24	17	16	14	19	16

Artery wall Layer: Adventitia (L=1)

Thrombogenic Factor: Platelet (T=2)

Storage Medium Treatment: 0 weeks (j=2)

Count of Total Measurements: 162 (n_2=162)

measured values of $y_{k,r,2}^{1,2}$ which is

the percentage of area covered by Platelet

in the region k of Adventitia layer from donor r

after 0 weeks treatment with storage medium

	r=1	r=2	r=3	r=4	r=5	r=6	r=7	r=8	r=9	r=10	r=11
k=1	11.2	17.1	39.2	27.9	58.6	6.6	25.8	13.1	15.5	9.6	15.7
k=2	45.7	16.7	29.0	23.3	26.9	4.2	37.1	11.6	17.9	11.4	19.0
k=3	7.2	14.8	19.9	22.7	29.7	7.2	28.9	23.0	9.6	10.2	16.1
k=4	4.6	15.8	25.7	17.5	57.9	9.5	17.8	34.7	12.8	16.3	11.4
k=5	7.9	18.9	21.0	18.2	47.0	7.8	15.5	33.8	7.5	6.5	11.6
k=6	16.0	16.8	36.2	9.6	41.2	8.5	28.7	30.7	26.9	8.7	40.9
k=7	26.4	N/A	50.5	6.2	36.5	6.2	12.5	33.1	11.5	10.4	30.9
k=8	24.9	N/A	17.5	29.6	36.7	8.4	32.2	32.3	16.4	10.8	36.6
k=9	13.1	N/A	28.6	31.5	N/A	8.6	24.6	30.3	14.4	14.3	28.4

k=10	19.4	N/A	21.3	27.3	N/A	9.2	38.9	14.7	25.8	3.8	12.0
k=11	N/A	N/A	23.8	15.0	N/A	10.0	17.1	9.3	17.2	19.9	9.7
k=12	N/A	N/A	N/A	19.3	N/A	10.5	15.2	25.8	19.9	16.3	18.5
k=13	N/A	N/A	N/A	15.6	N/A	9.3	17.6	27.3	20.5	18.4	38.6
k=14	N/A	N/A	N/A	15.4	N/A	10.9	17.6	16.6	18.7	13.6	18.6
k=15	N/A	N/A	N/A	19.4	N/A	11.1	14.8	N/A	16.3	17.7	18.3
k=16	N/A	N/A	N/A	13.0	N/A	10.5	18.9	N/A	16.0	13.7	10.1
k=17	N/A	N/A	N/A	17.9	N/A	5.4	21.1	N/A	11.2	13.6	19.7
k=18	N/A	N/A	N/A	17.0	N/A	5.5	N/A	N/A	N/A	13.8	39.8
k=19	N/A	N/A	N/A	N/A	N/A	4.2	N/A	N/A	N/A	18.3	N/A
k=20	N/A	N/A	N/A	N/A	N/A	4.5	N/A	N/A	N/A	N/A	N/A
k=21	N/A	N/A	N/A	N/A	N/A	6.5	N/A	N/A	N/A	N/A	N/A
k=22	N/A	N/A	N/A	N/A	N/A	10.0	N/A	N/A	N/A	N/A	N/A
k=23	N/A	N/A	N/A	N/A	N/A	5.0	N/A	N/A	N/A	N/A	N/A
k=24	N/A	N/A	N/A	N/A	N/A	7.8	N/A	N/A	N/A	N/A	N/A

values of $m_{r,2}^{1,2}$ which is

the count of regions covered by Platelet

in Adventitia layer from donor r

after 0 weeks treatment with storage medium

r	1	2	3	4	5	6	7	8	9	10	11
count	10	6	11	18	8	24	17	14	17	19	18

Artery wall Layer: Adventitia (L=1)

Thrombogenic Factor: Platelet (T=2)

Storage Medium Treatment: 1 week (j=3)

Count of Total Measurements: 155 (n_3=155)

measured values of $y_{k,r,3}^{1,2}$ which is

the percentage of area covered by Platelet

in the region k of Adventitia layer from donor r

after 1 week treatment with storage medium

	r=1	r=2	r=3	r=4	r=5	r=6	r=7	r=8	r=9	r=10	r=11
k=1	3.7	34.8	32.7	16.0	43.8	27.5	23.3	33.4	8.3	13.5	6.0
k=2	9.5	54.9	31.7	18.1	45.3	19.1	18.8	28.7	8.6	12.8	14.6
k=3	9.2	13.1	27.3	13.3	45.8	49.8	15.1	26.2	11.2	14.7	15.7
k=4	6.5	24.3	30.7	19.6	37.2	25.3	6.7	46.2	12.6	13.8	10.4
k=5	7.9	33.5	27.3	25.0	39.2	18.2	20.6	26.7	11.6	18.2	12.1
k=6	8.9	24.8	39.0	17.6	33.8	14.8	4.8	19.5	11.4	7.5	4.9
k=7	10.9	11.3	25.6	30.7	27.9	22.8	26.4	27.8	5.6	10.1	7.1
k=8	14.6	44.1	26.7	15.1	32.5	27.9	20.6	8.1	5.1	16.1	6.9
k=9	15.4	29.4	21.2	21.6	40.1	25.0	19.3	10.1	9.4	10.6	10.7

k=10	17.5	19.7	17.8	25.3	19.9	27.0	16.1	12.5	8.1	8.1	16.1
k=11	10.8	14.0	9.5	14.8	14.5	25.3	20.7	22.7	N/A	5.9	14.0
k=12	15.4	19.6	9.9	20.6	30.9	20.9	N/A	27.7	N/A	5.3	16.1
k=13	17.2	12.3	N/A	33.4	37.1	27.1	N/A	28.6	N/A	N/A	9.7
k=14	9.0	27.4	N/A	14.1	18.9	31.9	N/A	11.6	N/A	N/A	5.4
k=15	N/A	N/A	N/A	4.1	N/A	39.2	N/A	24.3	N/A	N/A	N/A
k=16	N/A	N/A	N/A	21.0	N/A	31.6	N/A	16.0	N/A	N/A	N/A
k=17	N/A	N/A	N/A	13.8	N/A	N/A	N/A	24.5	N/A	N/A	N/A
k=18	N/A	N/A	N/A	20.9	N/A	N/A	N/A	28.7	N/A	N/A	N/A
k=19	N/A	N/A	N/A	19.5	N/A	N/A	N/A	N/A	N/A	N/A	N/A
k=20	N/A	N/A	N/A	16.4	N/A	N/A	N/A	N/A	N/A	N/A	N/A

values of $m_{r,3}^{1,2}$ which is

the count of regions covered by Platelet

in Adventitia layer from donor r

after 1 week treatment with storage medium

r	1	2	3	4	5	6	7	8	9	10	11
count	14	14	12	20	14	16	11	18	10	12	14

|________|___|___|___|___|___|___|___|___|___|___|___|

Artery wall Layer: Adventitia (L=1)

Thrombogenic Factor: Platelet (T=2)

Storage Medium Treatment: 12 weeks (j=4)

Count of Total Measurements: 211 (n_4=211)

measured values of $y_{k,r,4}^{1,2}$ which is

the percentage of area covered by Platelet

in the region k of Adventitia layer from donor r

after 12 weeks treatment with storage medium

	r=1	r=2	r=3	r=4	r=5	r=6	r=7	r=8	r=9	r=10	r=11
k=1	7.9	21.6	27.1	18.4	26.4	14.2	18.1	20.4	9.8	8.1	5.5
k=2	5.2	20.7	23.0	21.0	17.1	15.7	21.9	27.1	9.8	9.1	13.9
k=3	3.9	22.5	24.3	15.1	30.8	17.9	30.8	30.2	5.5	7.3	11.5
k=4	3.5	35.3	23.3	9.2	37.2	16.0	41.3	20.6	2.7	8.3	2.5
k=5	7.3	40.9	24.6	24.2	24.6	13.4	34.1	27.3	4.8	15.0	6.3
k=6	17.8	31.0	16.3	21.6	19.7	5.4	36.5	26.3	3.5	10.9	10.1
k=7	9.9	14.7	19.0	7.3	14.8	13.7	31.6	16.9	7.9	15.8	9.9
k=8	7.1	14.9	13.7	10.6	19.6	11.0	39.1	20.3	4.7	14.6	12.5
k=9	5.6	36.4	20.3	10.6	18.5	16.9	30.3	26.7	2.5	16.9	16.5

k=10	8.1	19.1	9.0	28.9	27.2	25.4	37.1	16.1	3.8	12.8	12.5
k=11	7.6	21.6	26.9	21.4	8.0	24.5	35.9	26.5	6.6	8.0	15.9
k=12	12.9	16.7	41.9	14.9	9.1	21.4	33.2	17.6	10.3	9.9	10.2
k=13	10.8	37.4	37.6	18.9	20.4	26.3	33.3	23.5	9.2	16.3	8.7
k=14	7.6	41.6	32.9	13.3	10.3	N/A	25.9	19.3	3.6	11.0	10.9
k=15	13.7	40.3	N/A	19.0	14.0	N/A	26.0	26.3	2.7	N/A	10.1
k=16	8.5	49.5	N/A	16.3	12.7	N/A	21.9	23.4	3.6	N/A	10.3
k=17	9.7	23.1	N/A	N/A	15.6	N/A	36.0	26.8	12.3	N/A	12.2
k=18	11.0	27.2	N/A	N/A	12.4	N/A	39.7	27.6	6.9	N/A	15.4
k=19	9.0	19.4	N/A	N/A	N/A	N/A	N/A	39.1	11.3	N/A	10.9
k=20	14.5	30.2	N/A	N/A	N/A	N/A	N/A	17.2	10.4	N/A	8.4
k=21	14.7	20.5	N/A	N/A	N/A	N/A	N/A	19.3	9.3	N/A	15.1
k=22	10.5	44.7	N/A	N/A	N/A	N/A	N/A	N/A	8.4	N/A	19.9
k=23	26.5	39.4	N/A	N/A	N/A	N/A	N/A	N/A	N/A	N/A	11.0
k=24	8.2	35.4	N/A	N/A	N/A	N/A	N/A	N/A	N/A	N/A	12.5
k=25	N/A	21.0	N/A	N/A	N/A	N/A	N/A	N/A	N/A	N/A	8.6

k=26	N/A	N/A	N/A	N/A	N/A	N/A	N/A	N/A	N/A	N/A	7.3

values of $m_{r,4}^{1,2}$ which is

the count of regions covered by Platelet

in Adventitia layer from donor r

after 12 weeks treatment with storage medium

r	1	2	3	4	5	6	7	8	9	10	11
count	24	25	14	16	18	13	18	21	22	14	26

Artery wall Layer: Adventitia (*L*=1)

Thrombogenic Factor: Platelet (T=2)

Storage Medium Treatment: 24 weeks (*j*=5)

Count of Total Measurements: 176 (n_5=176)

measured values of $y_{k,r,5}^{1,2}$ which is

the percentage of area covered by Platelet

in the region *k* of Adventitia layer from donor *r*

after 24 weeks treatment with storage medium

	r=1	*r*=2	*r*=3	*r*=4	*r*=5	*r*=6	*r*=7	*r*=8	*r*=9	*r*=10	*r*=11
k=1	23.6	12.6	8.7	11.3	10.1	14.5	28.3	16.1	6.4	14.4	3.2
k=2	21.5	11.8	12.2	13.1	16.6	17.4	24.6	21.4	4.7	16.9	10.1
k=3	7.8	10.4	15.1	12.6	21.4	19.0	35.1	19.2	5.1	11.8	12.1
k=4	9.3	31.8	5.9	13.1	14.1	11.4	33.0	27.4	5.0	7.9	6.4
k=5	17.6	17.2	7.7	20.3	28.3	13.6	34.8	21.8	11.1	15.7	13.8
k=6	10.4	18.7	12.9	15.7	30.2	8.6	23.9	19.9	4.1	15.7	11.9
k=7	17.9	19.7	11.7	18.5	20.7	13.8	27.1	26.2	8.0	8.5	4.5
k=8	16.7	20.1	7.7	13.2	14.7	11.0	17.1	11.1	10.5	7.3	8.2
k=9	21.1	17.1	8.8	13.1	14.5	9.3	11.4	9.5	17.0	5.5	4.2

k=10	34.3	10.3	9.6	22.4	16.1	13.0	17.1	29.5	7.5	7.5	9.1
k=11	N/A	N/A	N/A	19.5	11.3	9.7	13.6	17.7	6.2	8.3	5.5
k=12	N/A	N/A	N/A	N/A	17.5	8.4	11.5	21.0	5.8	4.4	3.3
k=13	N/A	N/A	N/A	N/A	24.0	15.5	25.2	16.4	4.7	7.5	3.3
k=14	N/A	N/A	N/A	N/A	20.3	7.8	11.4	18.4	4.8	12.6	10.3
k=15	N/A	N/A	N/A	N/A	17.1	N/A	14.5	23.0	8.5	8.6	4.1
k=16	N/A	N/A	N/A	N/A	27.7	N/A	33.7	27.2	5.1	8.7	3.7
k=17	N/A	N/A	N/A	N/A	37.4	N/A	19.0	N/A	4.7	9.8	7.3
k=18	N/A	N/A	N/A	N/A	35.9	N/A	28.0	N/A	5.2	13.8	5.8
k=19	N/A	N/A	N/A	N/A	N/A	N/A	19.0	N/A	5.2	13.2	1.2
k=20	N/A	N/A	N/A	N/A	N/A	N/A	14.5	N/A	N/A	12.8	3.7
k=21	N/A	N/A	N/A	N/A	N/A	N/A	23.9	N/A	N/A	N/A	3.7
k=22	N/A	N/A	N/A	N/A	N/A	N/A	22.1	N/A	N/A	N/A	14.6
k=23	N/A	N/A	N/A	N/A	N/A	N/A	19.2	N/A	N/A	N/A	N/A
k=24	N/A	N/A	N/A	N/A	N/A	N/A	27.2	N/A	N/A	N/A	N/A
k=25	N/A	N/A	N/A	N/A	N/A	N/A	28.7	N/A	N/A	N/A	N/A

k=26	N/A	N/A	N/A	N/A	N/A	N/A	29.4	N/A	N/A	N/A	N/A

values of $m_{r,5}^{1,2}$ which is

the count of regions covered by Platelet

in Adventitia layer from donor r

after 24 weeks treatment with storage medium

r	1	2	3	4	5	6	7	8	9	10	11
count	10	10	10	11	18	14	26	16	19	20	22
