# Supplementary material for: Cryopreservation moderates the thrombogenicity of arterial allografts during storage
Source: PLoS One. 2021 Jul 22;16(7):e0255114. doi: 10.1371/journal.pone.0255114 (PMC8297765; doi:10.1371/journal.pone.0255114)
Supplement: S1 Data — (ZIP) [file pone.0255114.s002.zip › Open_data_R1/Data_L2_T1.pdf]

Artery wall Layer: Media (L=2)

Thrombogenic Factor: Fibrin (T=1)

Storage Medium Treatment: No treatment (j=1)

Count of Total Measurements: 153 (n_1=153)

measured values of $y_{k,r,1}^{2,1}$ which is

the percentage of area covered by Fibrin

in the region k of Media layer from donor r

with No treatment by storage medium

	r=1	r=2	r=3	r=4	r=5	r=6	r=7	r=8	r=9	r=10	r=11
k=1	17.9	15.5	9.4	17.2	6.1	1.2	4.9	1.1	9.1	2.4	0.7
k=2	45.8	15.2	14.8	7.0	6.7	5.6	5.3	0.8	1.9	3.3	1.9
k=3	52.2	14.0	25.9	5.7	10.2	0.9	1.9	0.6	2.8	25.0	0.4
k=4	25.6	17.3	31.7	5.9	1.2	5.1	6.2	0.5	3.9	6.0	0.6
k=5	33.0	15.6	25.3	4.4	1.1	0.9	3.6	0.2	3.0	1.8	0.5
k=6	30.6	18.5	15.1	2.8	8.1	5.1	5.6	0.2	2.8	1.1	0.4
k=7	25.6	9.3	18.5	1.3	5.6	1.8	4.3	1.1	0.5	0.0	1.2
k=8	N/A	9.4	20.3	3.3	4.5	1.8	4.5	1.4	2.9	2.5	0.1
k=9	N/A	16.5	18.3	15.3	N/A	1.9	1.3	1.1	2.2	2.8	2.1

k=10	N/A	12.5	N/A	12.1	N/A	0.8	7.4	3.1	1.3	6.7	11.8
k=11	N/A	20.1	N/A	30.8	N/A	1.5	3.0	0.3	1.2	2.8	3.4
k=12	N/A	22.6	N/A	4.9	N/A	1.5	8.8	1.1	0.1	3.2	2.1
k=13	N/A	N/A	N/A	5.2	N/A	10.1	23.1	1.1	0.5	5.8	0.4
k=14	N/A	N/A	N/A	28.0	N/A	7.0	11.4	4.4	0.2	N/A	0.2
k=15	N/A	N/A	N/A	N/A	N/A	3.6	11.3	2.6	0.6	N/A	0.3
k=16	N/A	N/A	N/A	N/A	N/A	6.4	N/A	5.2	3.9	N/A	1.6
k=17	N/A	N/A	N/A	N/A	N/A	3.6	N/A	6.3	N/A	N/A	N/A
k=18	N/A	N/A	N/A	N/A	N/A	8.8	N/A	N/A	N/A	N/A	N/A
k=19	N/A	N/A	N/A	N/A	N/A	5.5	N/A	N/A	N/A	N/A	N/A
k=20	N/A	N/A	N/A	N/A	N/A	14.9	N/A	N/A	N/A	N/A	N/A
k=21	N/A	N/A	N/A	N/A	N/A	21.3	N/A	N/A	N/A	N/A	N/A
k=22	N/A	N/A	N/A	N/A	N/A	2.1	N/A	N/A	N/A	N/A	N/A
k=23	N/A	N/A	N/A	N/A	N/A	3.2	N/A	N/A	N/A	N/A	N/A
k=24	N/A	N/A	N/A	N/A	N/A	1.5	N/A	N/A	N/A	N/A	N/A
k=25	N/A	N/A	N/A	N/A	N/A	2.7	N/A	N/A	N/A	N/A	N/A

k=26	N/A	N/A	N/A	N/A	N/A	2.0	N/A	N/A	N/A	N/A	N/A

values of $m_{r,1}^{2,1}$ which is
the count of regions covered by Fibrin
in Media layer from donor r
with No treatment by storage medium

r	1	2	3	4	5	6	7	8	9	10	11
count	7	12	9	14	8	26	15	17	16	13	16

Artery wall Layer: Media (L=2)

Thrombogenic Factor: Fibrin (T=1)

Storage Medium Treatment: 0 weeks (j=2)

Count of Total Measurements: 160 (n_2=160)

measured values of $y_{k,r,2}^{2,1}$ which is

the percentage of area covered by Fibrin

in the region k of Media layer from donor r

after 0 weeks treatment with storage medium

	r=1	r=2	r=3	r=4	r=5	r=6	r=7	r=8	r=9	r=10	r=11
k=1	28.1	14.8	7.0	2.9	26.5	0.8	3.1	0.2	0.1	0.1	0.0
k=2	32.7	14.1	6.9	0.5	16.2	0.7	1.4	0.7	0.1	0.2	0.1
k=3	45.7	15.1	13.8	29.8	12.7	4.7	1.3	0.5	0.2	2.9	0.4
k=4	45.2	12.3	14.6	66.2	12.1	1.1	1.7	0.5	0.3	3.6	1.0
k=5	19.2	11.9	8.0	17.9	2.8	4.7	3.6	0.8	0.4	4.3	1.4
k=6	8.5	21.6	10.1	28.4	22.9	9.0	3.3	0.9	0.1	3.8	0.5
k=7	34.0	22.8	7.8	13.7	13.7	5.4	2.6	2.0	0.3	3.6	0.1
k=8	22.5	46.7	8.9	3.7	27.9	0.2	7.0	1.6	0.2	6.9	0.7
k=9	35.7	23.3	12.0	2.5	N/A	0.2	13.1	4.4	0.1	2.6	0.3

k=10	23.2	43.1	9.6	1.4	N/A	0.2	7.5	2.8	0.0	7.1	0.3
k=11	41.7	N/A	10.2	1.4	N/A	0.2	11.6	5.2	0.0	0.1	N/A
k=12	36.6	N/A	9.5	4.8	N/A	0.2	1.2	0.0	0.2	1.0	N/A
k=13	22.0	N/A	9.2	10.6	N/A	0.1	2.3	1.5	0.1	1.7	N/A
k=14	N/A	N/A	10.2	0.9	N/A	0.1	2.2	4.6	0.0	0.0	N/A
k=15	N/A	N/A	N/A	13.3	N/A	0.6	21.1	N/A	0.4	1.7	N/A
k=16	N/A	N/A	N/A	5.3	N/A	0.9	27.8	N/A	0.4	2.2	N/A
k=17	N/A	N/A	N/A	N/A	N/A	0.9	3.6	N/A	0.5	N/A	N/A
k=18	N/A	N/A	N/A	N/A	N/A	0.1	N/A	N/A	1.2	N/A	N/A
k=19	N/A	N/A	N/A	N/A	N/A	0.6	N/A	N/A	0.6	N/A	N/A
k=20	N/A	N/A	N/A	N/A	N/A	0.4	N/A	N/A	0.2	N/A	N/A
k=21	N/A	N/A	N/A	N/A	N/A	0.2	N/A	N/A	N/A	N/A	N/A
k=22	N/A	N/A	N/A	N/A	N/A	0.5	N/A	N/A	N/A	N/A	N/A

values of $m_{r,2}^{2,1}$ which is

the count of regions covered by Fibrin

in Media layer from donor r

after 0 weeks treatment with storage medium

r	1	2	3	4	5	6	7	8	9	10	11
count	13	10	14	16	8	22	17	14	20	16	10

Artery wall Layer: Media (L=2)

Thrombogenic Factor: Fibrin (T=1)

Storage Medium Treatment: 1 week (j=3)

Count of Total Measurements: 166 (n_3=166)

measured values of $y_{k,r,3}^{2,1}$ which is

the percentage of area covered by Fibrin

in the region k of Media layer from donor r

after 1 week treatment with storage medium

	r=1	r=2	r=3	r=4	r=5	r=6	r=7	r=8	r=9	r=10	r=11
k=1	18.1	3.2	8.7	4.4	32.9	2.2	1.4	2.4	0.6	1.8	0.5
k=2	17.2	25.5	6.5	55.4	10.9	6.0	2.6	1.4	0.8	1.7	1.1
k=3	24.3	15.1	4.8	11.0	5.7	6.5	3.3	3.8	0.5	0.7	0.2
k=4	20.8	11.0	8.3	3.4	10.7	4.7	0.4	29.8	0.3	1.2	5.3
k=5	24.5	22.2	9.6	11.6	15.3	10.1	0.7	3.0	0.3	3.1	0.9
k=6	17.4	20.7	11.8	4.9	14.3	15.4	0.8	4.8	0.7	2.1	0.8
k=7	34.2	17.4	9.6	11.9	3.8	17.3	1.1	3.5	0.4	12.8	0.2
k=8	25.6	N/A	9.1	0.2	20.3	11.1	1.4	4.8	0.6	7.7	0.3
k=9	17.1	N/A	7.0	3.0	16.6	10.4	1.4	0.3	0.4	1.3	9.7

k=10	14.6	N/A	10.3	16.8	2.3	2.7	2.2	8.3	0.4	2.4	35.2
k=11	9.6	N/A	8.3	4.7	14.4	5.4	1.1	3.8	0.7	1.5	1.6
k=12	9.4	N/A	14.7	12.5	16.3	2.8	N/A	8.0	1.2	7.7	0.4
k=13	N/A	N/A	11.8	17.6	6.3	2.2	N/A	0.2	6.9	1.9	0.3
k=14	N/A	N/A	11.9	14.6	N/A	2.0	N/A	1.1	0.2	0.9	N/A
k=15	N/A	N/A	N/A	N/A	N/A	3.2	N/A	2.1	1.0	0.7	N/A
k=16	N/A	N/A	N/A	N/A	N/A	1.2	N/A	1.9	1.3	3.4	N/A
k=17	N/A	N/A	N/A	N/A	N/A	3.9	N/A	6.2	0.4	1.6	N/A
k=18	N/A	N/A	N/A	N/A	N/A	1.6	N/A	12.0	0.2	N/A	N/A
k=19	N/A	N/A	N/A	N/A	N/A	4.0	N/A	3.2	0.1	N/A	N/A
k=20	N/A	N/A	N/A	N/A	N/A	4.2	N/A	2.4	0.1	N/A	N/A
k=21	N/A	N/A	N/A	N/A	N/A	6.4	N/A	5.2	N/A	N/A	N/A
k=22	N/A	N/A	N/A	N/A	N/A	N/A	N/A	7.5	N/A	N/A	N/A
k=23	N/A	N/A	N/A	N/A	N/A	N/A	N/A	2.6	N/A	N/A	N/A
k=24	N/A	N/A	N/A	N/A	N/A	N/A	N/A	0.3	N/A	N/A	N/A

values of $m_{r,3}^{2,1}$ which is

the count of regions covered by Fibrin

in Media layer from donor *r*

after 1 week treatment with storage medium

r	1	2	3	4	5	6	7	8	9	10	11
count	12	7	14	14	13	21	11	24	20	17	13

Artery wall Layer: Media (L=2)

Thrombogenic Factor: Fibrin (T=1)

Storage Medium Treatment: 12 weeks (j=4)

Count of Total Measurements: 211 (n_4=211)

measured values of $y^{2,1}_{k,r,4}$ which is

the percentage of area covered by Fibrin

in the region k of Media layer from donor r

after 12 weeks treatment with storage medium

	r=1	r=2	r=3	r=4	r=5	r=6	r=7	r=8	r=9	r=10	r=11
k=1	1.8	2.5	4.5	6.7	4.4	1.8	1.7	2.4	0.2	0.1	0.7
k=2	0.6	1.8	5.0	4.1	1.9	3.2	4.9	4.7	0.2	0.0	0.9
k=3	0.5	1.2	5.6	1.6	2.4	5.6	1.9	8.3	0.1	0.1	3.7
k=4	0.4	2.1	5.4	4.8	1.8	2.5	6.4	7.8	0.1	0.2	0.8
k=5	1.5	1.3	5.6	3.2	2.7	2.9	8.6	7.2	0.1	0.1	0.3
k=6	1.3	4.0	6.3	3.6	2.5	4.1	8.1	4.5	0.0	0.2	1.1
k=7	0.3	4.5	3.8	3.7	3.3	3.6	6.6	12.0	0.3	0.2	0.2
k=8	0.2	2.7	3.6	1.5	2.9	1.7	4.7	25.8	0.3	0.2	0.1
k=9	0.5	18.2	2.1	1.7	2.1	3.8	2.2	12.2	0.2	0.7	0.2

k=10	0.3	19.4	1.7	1.3	1.4	2.3	1.0	25.5	0.7	0.3	0.1
k=11	6.6	0.1	10.0	2.8	6.8	3.1	2.0	4.0	0.2	0.7	0.1
k=12	0.5	1.6	12.2	3.1	6.5	9.8	0.5	9.0	5.8	3.0	0.0
k=13	0.5	2.6	11.6	1.4	10.7	7.0	5.2	6.3	0.1	1.3	0.1
k=14	0.4	2.6	10.5	7.2	10.9	3.6	6.4	6.5	0.1	0.3	0.1
k=15	0.4	1.3	N/A	3.4	11.0	2.6	6.5	1.6	0.3	0.2	0.1
k=16	0.2	5.0	N/A	0.1	0.6	3.0	0.7	1.7	0.4	N/A	0.4
k=17	0.4	2.2	N/A	N/A	2.7	4.0	0.5	1.3	0.6	N/A	0.1
k=18	0.9	2.2	N/A	N/A	4.8	3.4	N/A	0.3	N/A	N/A	0.0
k=19	1.2	9.6	N/A	N/A	8.1	3.5	N/A	2.0	N/A	N/A	0.0
k=20	1.2	5.0	N/A	N/A	N/A	N/A	N/A	9.0	N/A	N/A	0.1
k=21	1.9	4.5	N/A	N/A	N/A	N/A	N/A	17.4	N/A	N/A	0.1
k=22	N/A	17.3	N/A	N/A	N/A	N/A	N/A	0.2	N/A	N/A	0.1
k=23	N/A	10.7	N/A	N/A	N/A	N/A	N/A	0.3	N/A	N/A	0.1
k=24	N/A	7.0	N/A	N/A	N/A	N/A	N/A	0.7	N/A	N/A	0.0
k=25	N/A	0.2	N/A	N/A	N/A	N/A	N/A	N/A	N/A	N/A	N/A

values of $m_{r,4}^{2,1}$ which is

the count of regions covered by Fibrin

in Media layer from donor r

after 12 weeks treatment with storage medium

r	1	2	3	4	5	6	7	8	9	10	11
count	21	25	14	16	19	19	17	24	17	15	24

Artery wall Layer: Media (L=2)

Thrombogenic Factor: Fibrin (T=1)

Storage Medium Treatment: 24 weeks (j=5)

Count of Total Measurements: 219 (n_5=219)

measured values of $y_{k,r,5}^{2,1}$ which is

the percentage of area covered by Fibrin

in the region k of Media layer from donor r

after 24 weeks treatment with storage medium

	r=1	r=2	r=3	r=4	r=5	r=6	r=7	r=8	r=9	r=10	r=11
k=1	0.8	0.8	5.7	0.7	1.4	9.4	12.7	4.8	0.1	0.1	0.0
k=2	0.2	0.8	2.8	21.4	1.1	8.0	10.3	4.4	1.9	0.2	0.0
k=3	0.1	0.5	2.5	8.7	0.8	23.7	16.6	5.5	0.2	0.2	0.1
k=4	0.3	0.5	3.8	7.5	0.7	10.0	10.7	7.8	2.5	0.1	0.5
k=5	0.3	2.4	3.6	3.5	0.6	14.6	9.7	10.3	0.2	4.1	0.3
k=6	1.1	1.1	3.9	5.9	0.4	10.2	9.1	7.6	0.2	0.4	0.3
k=7	1.5	0.1	2.9	6.1	0.5	10.5	6.9	7.0	0.1	0.0	2.4
k=8	1.0	0.1	1.6	7.1	1.6	19.4	3.3	7.0	0.1	0.0	0.8
k=9	5.3	0.3	1.9	5.8	2.2	14.4	8.5	8.0	0.4	0.5	0.2

k=10	3.2	6.0	5.2	25.0	3.1	29.7	3.7	12.8	0.8	2.7	0.5
k=11	4.8	7.3	3.7	6.0	2.1	11.0	4.1	7.4	0.3	7.4	0.7
k=12	2.4	9.4	2.7	1.5	2.3	20.4	6.2	8.9	0.4	2.2	0.9
k=13	2.1	3.0	2.3	15.2	2.5	42.0	9.6	5.3	0.7	1.8	0.3
k=14	1.9	1.8	3.8	5.7	2.8	12.7	5.6	5.4	0.7	0.5	0.5
k=15	N/A	1.7	1.8	1.7	3.5	N/A	6.7	6.0	0.7	4.3	4.9
k=16	N/A	0.8	1.3	N/A	2.9	N/A	9.1	28.0	0.5	0.3	0.0
k=17	N/A	1.8	0.9	N/A	3.5	N/A	6.1	1.7	0.9	1.0	1.1
k=18	N/A	1.0	1.8	N/A	N/A	N/A	10.9	13.3	0.8	3.9	1.2
k=19	N/A	0.2	1.0	N/A	N/A	N/A	10.5	17.3	0.8	0.2	1.1
k=20	N/A	0.6	3.4	N/A	N/A	N/A	N/A	N/A	0.8	0.0	0.5
k=21	N/A	N/A	N/A	N/A	N/A	N/A	N/A	N/A	1.7	0.0	0.1
k=22	N/A	N/A	N/A	N/A	N/A	N/A	N/A	N/A	0.4	0.5	0.4
k=23	N/A	N/A	N/A	N/A	N/A	N/A	N/A	N/A	N/A	0.4	0.5
k=24	N/A	N/A	N/A	N/A	N/A	N/A	N/A	N/A	N/A	0.1	0.3
k=25	N/A	N/A	N/A	N/A	N/A	N/A	N/A	N/A	N/A	0.1	0.2

k=26	N/A	N/A	N/A	N/A	N/A	N/A	N/A	N/A	N/A	0.4	0.2
k=27	N/A	N/A	N/A	N/A	N/A	N/A	N/A	N/A	N/A	0.7	0.1
k=28	N/A	N/A	N/A	N/A	N/A	N/A	N/A	N/A	N/A	0.3	0.0
k=29	N/A	N/A	N/A	N/A	N/A	N/A	N/A	N/A	N/A	0.3	0.1
k=30	N/A	N/A	N/A	N/A	N/A	N/A	N/A	N/A	N/A	N/A	0.1

values of $m_{r,5}^{2,1}$ which is

the count of regions covered by Fibrin

in Media layer from donor r

after 24 weeks treatment with storage medium

r	1	2	3	4	5	6	7	8	9	10	11
count	14	20	20	15	17	14	19	19	22	29	30
