# Supplementary material for: Cryopreservation moderates the thrombogenicity of arterial allografts during storage
Source: PLoS One. 2021 Jul 22;16(7):e0255114. doi: 10.1371/journal.pone.0255114 (PMC8297765; doi:10.1371/journal.pone.0255114)
Supplement: S1 Data — (ZIP) [file pone.0255114.s002.zip › Open_data_R1/Data_L2_T2.pdf]

Artery wall Layer: Media (L=2)

Thrombogenic Factor: Platelet (T=2)

Storage Medium Treatment: No treatment (j=1)

Count of Total Measurements: 160 (n_1=160)

measured values of $y_{k,r,1}^{2,2}$ which is

the percentage of area covered by Platelet

in the region k of Media layer from donor r

with No treatment by storage medium

	r=1	r=2	r=3	r=4	r=5	r=6	r=7	r=8	r=9	r=10	r=11
k=1	2.5	13.3	2.6	15.0	3.2	2.8	1.9	3.8	1.0	3.1	4.5
k=2	3.3	10.9	3.0	11.6	4.1	3.3	1.2	5.3	1.2	3.3	4.4
k=3	3.4	13.7	3.5	9.2	5.2	2.6	2.2	4.2	0.8	6.6	2.6
k=4	6.8	14.5	3.9	5.6	2.4	6.0	17.7	6.8	1.4	3.6	3.2
k=5	6.6	17.0	4.9	2.1	2.2	2.6	9.8	1.7	2.5	4.9	3.4
k=6	9.7	14.7	4.3	6.0	8.7	6.0	5.5	0.6	3.2	3.5	3.9
k=7	4.2	11.4	3.9	4.7	5.2	3.1	14.9	3.7	2.3	5.8	3.8
k=8	N/A	12.8	7.7	9.2	9.6	2.1	7.4	3.7	3.6	6.2	3.2
k=9	N/A	7.6	3.6	4.7	N/A	2.7	10.9	0.1	3.1	7.3	1.5

k=10	N/A	4.6	N/A	5.7	N/A	1.9	5.7	0.4	3.1	3.6	2.2
k=11	N/A	15.1	N/A	6.9	N/A	2.1	15.5	1.3	3.4	3.5	1.4
k=12	N/A	17.8	N/A	7.7	N/A	2.3	10.7	1.1	0.9	6.1	1.4
k=13	N/A	N/A	N/A	6.6	N/A	4.9	5.7	0.3	1.2	12.8	1.0
k=14	N/A	N/A	N/A	8.5	N/A	2.5	1.8	0.6	1.4	N/A	2.1
k=15	N/A	N/A	N/A	1.6	N/A	4.9	3.4	0.5	1.2	N/A	4.6
k=16	N/A	N/A	N/A	3.4	N/A	1.3	N/A	4.0	0.2	N/A	1.8
k=17	N/A	N/A	N/A	1.8	N/A	9.0	N/A	3.1	N/A	N/A	N/A
k=18	N/A	N/A	N/A	24.5	N/A	1.2	N/A	N/A	N/A	N/A	N/A
k=19	N/A	N/A	N/A	6.3	N/A	1.3	N/A	N/A	N/A	N/A	N/A
k=20	N/A	N/A	N/A	9.9	N/A	3.1	N/A	N/A	N/A	N/A	N/A
k=21	N/A	N/A	N/A	3.0	N/A	2.4	N/A	N/A	N/A	N/A	N/A
k=22	N/A	N/A	N/A	4.7	N/A	2.6	N/A	N/A	N/A	N/A	N/A
k=23	N/A	N/A	N/A	N/A	N/A	2.2	N/A	N/A	N/A	N/A	N/A
k=24	N/A	N/A	N/A	N/A	N/A	3.5	N/A	N/A	N/A	N/A	N/A
k=25	N/A	N/A	N/A	N/A	N/A	1.5	N/A	N/A	N/A	N/A	N/A

values of $m_{r,1}^{2,2}$ which is

the count of regions covered by Platelet

in Media layer from donor *r*

with No treatment by storage medium

r	1	2	3	4	5	6	7	8	9	10	11
count	7	12	9	22	8	25	15	17	16	13	16

Artery wall Layer: Media (L=2)

Thrombogenic Factor: Platelet (T=2)

Storage Medium Treatment: 0 weeks (j=2)

Count of Total Measurements: 162 (n_2=162)

measured values of $y^{2,2}_{k,r,2}$ which is

the percentage of area covered by Platelet

in the region k of Media layer from donor r

after 0 weeks treatment with storage medium

	r=1	r=2	r=3	r=4	r=5	r=6	r=7	r=8	r=9	r=10	r=11
k=1	3.4	6.4	18.2	8.3	40.5	4.1	6.2	3.3	3.6	2.0	7.0
k=2	5.3	6.3	13.8	9.9	12.2	3.7	4.4	4.0	5.4	2.1	7.9
k=3	2.7	6.3	20.4	12.1	24.6	2.6	3.9	2.6	1.8	6.2	21.4
k=4	1.8	8.6	23.6	7.5	20.8	2.2	2.0	2.8	3.3	3.5	14.3
k=5	2.9	7.6	16.8	10.5	20.3	2.8	9.9	5.0	3.0	1.5	9.8
k=6	4.3	10.9	8.0	6.4	22.9	3.4	4.9	6.5	1.0	2.0	13.0
k=7	4.7	14.7	10.4	4.6	22.7	2.4	4.1	7.6	1.8	1.2	14.3
k=8	5.0	10.5	12.8	4.5	32.4	3.6	7.3	6.1	1.6	7.6	7.8
k=9	9.2	9.0	20.7	5.0	N/A	1.7	5.9	9.9	2.0	1.1	15.6

k=10	2.6	8.4	15.3	6.7	N/A	2.6	4.2	10.1	1.4	5.2	8.5
k=11	4.1	N/A	16.0	8.8	N/A	4.3	8.1	10.7	1.3	0.1	10.5
k=12	5.8	N/A	12.4	3.7	N/A	4.2	2.1	1.2	3.4	0.5	7.6
k=13	3.2	N/A	16.6	5.2	N/A	5.3	7.6	14.9	4.1	0.9	N/A
k=14	N/A	N/A	17.9	7.0	N/A	1.7	9.7	13.8	4.9	0.2	N/A
k=15	N/A	N/A	N/A	8.1	N/A	7.1	7.8	N/A	7.2	1.9	N/A
k=16	N/A	N/A	N/A	6.3	N/A	6.1	11.0	N/A	6.8	2.1	N/A
k=17	N/A	N/A	N/A	N/A	N/A	7.6	2.5	N/A	7.2	N/A	N/A
k=18	N/A	N/A	N/A	N/A	N/A	3.6	N/A	N/A	5.6	N/A	N/A
k=19	N/A	N/A	N/A	N/A	N/A	4.7	N/A	N/A	4.4	N/A	N/A
k=20	N/A	N/A	N/A	N/A	N/A	2.4	N/A	N/A	4.1	N/A	N/A
k=21	N/A	N/A	N/A	N/A	N/A	1.5	N/A	N/A	N/A	N/A	N/A
k=22	N/A	N/A	N/A	N/A	N/A	4.4	N/A	N/A	N/A	N/A	N/A

values of $m_{r,2}^{2,2}$ which is

the count of regions covered by Platelet

in Media layer from donor r

after 0 weeks treatment with storage medium

r	1	2	3	4	5	6	7	8	9	10	11
count	13	10	14	16	8	22	17	14	20	16	12

Artery wall Layer: Media (L=2)

Thrombogenic Factor: Platelet (T=2)

Storage Medium Treatment: 1 week (j=3)

Count of Total Measurements: 168 (n_3=168)

measured values of $y_{k,r,3}^{2,2}$ which is

the percentage of area covered by Platelet

in the region k of Media layer from donor r

after 1 week treatment with storage medium

	r=1	r=2	r=3	r=4	r=5	r=6	r=7	r=8	r=9	r=10	r=11
k=1	3.0	7.0	15.1	16.8	32.8	6.0	3.0	8.5	5.7	6.1	6.7
k=2	5.1	7.8	12.9	16.7	19.6	8.9	2.6	8.7	3.1	6.4	4.9
k=3	5.1	7.3	8.1	4.6	18.3	9.6	7.0	13.8	5.3	3.3	5.6
k=4	5.4	7.3	14.2	19.8	15.6	6.1	6.3	33.2	3.9	4.0	4.7
k=5	6.7	8.7	14.5	13.0	16.1	8.6	1.6	10.0	6.7	5.6	5.5
k=6	7.1	7.1	17.8	7.7	15.3	14.2	4.1	9.3	10.5	5.3	6.7
k=7	6.8	6.4	14.4	11.7	15.1	4.6	2.7	6.5	6.3	4.9	2.6
k=8	7.8	N/A	16.3	8.4	12.6	7.0	4.1	7.9	5.3	5.5	6.7
k=9	5.2	N/A	9.8	12.1	13.2	9.8	5.4	12.1	4.0	6.1	5.3

k=10	7.5	N/A	17.8	7.9	13.0	4.6	5.9	18.1	4.2	6.4	4.7
k=11	4.3	N/A	15.7	7.0	13.2	9.0	7.1	15.0	5.1	5.8	2.5
k=12	3.9	N/A	20.7	12.8	12.0	3.5	6.4	10.0	4.5	3.9	6.1
k=13	N/A	N/A	16.1	9.8	11.2	10.7	N/A	3.7	4.8	1.6	6.0
k=14	N/A	N/A	15.6	9.7	N/A	8.1	N/A	8.2	2.4	2.4	N/A
k=15	N/A	N/A	N/A	8.2	N/A	11.3	N/A	7.5	6.0	2.3	N/A
k=16	N/A	N/A	N/A	N/A	N/A	6.0	N/A	4.9	7.0	5.3	N/A
k=17	N/A	N/A	N/A	N/A	N/A	12.5	N/A	6.5	5.3	3.9	N/A
k=18	N/A	N/A	N/A	N/A	N/A	11.0	N/A	6.7	5.2	N/A	N/A
k=19	N/A	N/A	N/A	N/A	N/A	8.7	N/A	8.8	2.5	N/A	N/A
k=20	N/A	N/A	N/A	N/A	N/A	16.9	N/A	8.2	2.9	N/A	N/A
k=21	N/A	N/A	N/A	N/A	N/A	17.6	N/A	5.4	N/A	N/A	N/A
k=22	N/A	N/A	N/A	N/A	N/A	N/A	N/A	1.2	N/A	N/A	N/A
k=23	N/A	N/A	N/A	N/A	N/A	N/A	N/A	2.7	N/A	N/A	N/A
k=24	N/A	N/A	N/A	N/A	N/A	N/A	N/A	7.3	N/A	N/A	N/A

values of $m_{r,3}^{2,2}$ which is

the count of regions covered by Platelet

in Media layer from donor r

after 1 week treatment with storage medium

r	1	2	3	4	5	6	7	8	9	10	11
count	12	7	14	15	13	21	12	24	20	17	13

Artery wall Layer: Media (*L*=2)

Thrombogenic Factor: Platelet (*T*=2)

Storage Medium Treatment: 12 weeks (j=4)

Count of Total Measurements: 200 (n_4=200)

measured values of $y_{k,r,4}^{2,2}$ which is

the percentage of area covered by Platelet

in the region *k* of Media layer from donor *r*

after 12 weeks treatment with storage medium

	r=1	*r*=2	*r*=3	*r*=4	*r*=5	*r*=6	*r*=7	*r*=8	*r*=9	*r*=10	*r*=11
k=1	10.9	13.3	10.1	4.4	16.4	9.5	8.3	10.0	8.5	1.3	5.7
k=2	8.6	10.9	7.8	4.5	4.9	6.4	11.2	9.2	4.1	1.2	4.7
k=3	10.1	13.7	9.6	2.9	1.9	13.9	11.2	13.9	4.5	1.1	3.7
k=4	0.3	14.5	9.7	4.2	0.5	2.7	6.2	11.2	2.8	1.0	5.0
k=5	4.4	17.0	9.1	4.4	7.9	3.9	12.1	13.5	1.1	2.0	6.3
k=6	5.6	14.7	9.2	5.5	3.6	8.5	15.3	18.1	0.8	2.6	3.5
k=7	1.1	11.4	8.0	4.9	8.0	13.9	19.1	19.6	3.3	3.4	6.6
k=8	1.1	12.8	7.4	2.7	8.3	15.6	14.8	13.0	2.4	2.5	8.0
k=9	3.0	7.6	13.5	2.5	9.2	6.0	16.4	9.7	2.6	1.9	4.0

k=10	3.6	4.6	18.4	0.7	8.0	3.8	18.3	8.4	3.5	2.1	2.6
k=11	15.2	15.1	15.0	1.3	7.9	6.9	16.5	6.7	0.8	3.2	2.4
k=12	4.7	17.8	30.5	5.5	8.5	11.4	8.8	18.8	1.7	4.6	2.5
k=13	1.3	N/A	29.2	2.7	5.8	8.8	7.0	19.7	0.4	2.6	2.6
k=14	3.5	N/A	23.0	4.3	9.0	12.5	5.3	15.7	1.5	1.1	3.4
k=15	4.8	N/A	20.4	7.1	7.1	6.2	11.1	6.4	2.5	2.9	2.6
k=16	1.0	N/A	N/A	7.4	4.4	9.1	8.4	9.5	2.7	N/A	2.9
k=17	3.1	N/A	N/A	2.7	5.9	10.6	5.9	7.7	3.0	N/A	2.7
k=18	9.6	N/A	N/A	N/A	7.8	9.6	N/A	9.2	N/A	N/A	2.2
k=19	6.8	N/A	N/A	N/A	9.5	11.2	N/A	8.2	N/A	N/A	2.3
k=20	6.8	N/A	N/A	N/A	N/A	N/A	N/A	17.2	N/A	N/A	8.2
k=21	8.6	N/A	N/A	N/A	N/A	N/A	N/A	17.2	N/A	N/A	1.5
k=22	N/A	N/A	N/A	N/A	N/A	N/A	N/A	13.8	N/A	N/A	1.2
k=23	N/A	N/A	N/A	N/A	N/A	N/A	N/A	7.6	N/A	N/A	3.4
k=24	N/A	N/A	N/A	N/A	N/A	N/A	N/A	7.9	N/A	N/A	3.7

values of $m_{r,4}^{2,2}$ which is

the count of regions covered by Platelet

in Media layer from donor r

after 12 weeks treatment with storage medium

r	1	2	3	4	5	6	7	8	9	10	11
count	21	12	15	17	19	19	17	24	17	15	24

Artery wall Layer: Media (*L*=2)

Thrombogenic Factor: Platelet (*T*=2)

Storage Medium Treatment: 24 weeks (j=5)

Count of Total Measurements: 225 (n_5=225)

measured values of $y_{k,r,5}^{2,2}$ which is

the percentage of area covered by Platelet

in the region *k* of Media layer from donor *r*

after 24 weeks treatment with storage medium

	r=1	*r*=2	*r*=3	*r*=4	*r*=5	*r*=6	*r*=7	*r*=8	*r*=9	*r*=10	*r*=11
k=1	1.5	0.6	11.0	6.3	3.3	3.6	11.3	4.6	2.9	4.9	4.6
k=2	0.8	0.6	7.9	4.2	2.8	2.7	9.9	3.7	1.2	7.1	3.8
k=3	0.8	1.6	7.0	5.5	1.7	3.6	12.0	4.2	1.4	3.0	3.4
k=4	1.4	1.2	5.9	3.2	1.4	9.2	9.0	11.5	1.8	4.4	3.6
k=5	0.7	1.9	4.5	3.6	2.8	8.9	9.5	13.7	1.5	3.1	6.9
k=6	3.7	1.7	11.2	2.6	2.4	2.3	7.7	12.1	2.4	5.1	5.9
k=7	2.7	3.4	10.4	2.9	2.2	1.9	8.0	11.5	2.8	6.5	6.7
k=8	5.5	2.1	8.3	3.3	4.6	2.1	3.8	10.4	2.4	5.3	5.2
k=9	7.8	7.6	4.6	2.6	5.7	3.9	11.7	10.3	0.7	4.5	5.0

k=10	8.4	8.9	9.0	4.8	15.4	2.4	4.1	2.6	1.7	2.8	4.5
k=11	10.8	4.7	11.4	1.5	13.0	2.2	5.1	10.9	2.1	2.6	5.7
k=12	6.1	7.0	9.5	1.4	8.2	2.5	6.8	12.8	0.8	5.2	5.1
k=13	4.0	4.5	7.1	3.4	15.9	2.5	10.8	5.1	0.7	6.1	1.5
k=14	5.0	4.7	17.0	2.6	10.4	1.3	12.1	1.1	3.2	2.8	2.4
k=15	N/A	4.4	6.7	2.5	18.4	2.8	8.2	1.3	1.3	6.9	11.5
k=16	N/A	1.9	6.4	N/A	17.8	2.3	10.3	7.3	1.3	2.9	3.1
k=17	N/A	4.2	6.6	N/A	16.1	7.6	5.4	2.0	1.4	3.9	4.1
k=18	N/A	2.1	5.5	N/A	N/A	9.1	9.6	5.1	2.2	7.1	2.7
k=19	N/A	1.5	7.9	N/A	N/A	8.0	12.1	5.9	2.5	5.7	5.4
k=20	N/A	1.4	7.2	N/A	N/A	7.4	10.8	N/A	1.5	3.2	5.6
k=21	N/A	N/A	N/A	N/A	N/A	9.2	13.9	N/A	2.3	1.6	4.1
k=22	N/A	N/A	N/A	N/A	N/A	N/A	N/A	N/A	2.9	5.7	2.7
k=23	N/A	N/A	N/A	N/A	N/A	N/A	N/A	N/A	1.7	8.1	2.3
k=24	N/A	N/A	N/A	N/A	N/A	N/A	N/A	N/A	N/A	4.6	1.1
k=25	N/A	N/A	N/A	N/A	N/A	N/A	N/A	N/A	N/A	5.2	1.6

k=26	N/A	N/A	N/A	N/A	N/A	N/A	N/A	N/A	N/A	2.9	7.0
k=27	N/A	N/A	N/A	N/A	N/A	N/A	N/A	N/A	N/A	4.6	N/A
k=28	N/A	N/A	N/A	N/A	N/A	N/A	N/A	N/A	N/A	3.1	N/A
k=29	N/A	N/A	N/A	N/A	N/A	N/A	N/A	N/A	N/A	2.9	N/A

values of $m_{r,5}^{2,2}$ which is

the count of regions covered by Platelet

in Media layer from donor r

after 24 weeks treatment with storage medium

r	1	2	3	4	5	6	7	8	9	10	11
count	14	20	20	15	17	21	21	19	23	29	26
