# Supplementary material for: Cryopreservation moderates the thrombogenicity of arterial allografts during storage
Source: PLoS One. 2021 Jul 22;16(7):e0255114. doi: 10.1371/journal.pone.0255114 (PMC8297765; doi:10.1371/journal.pone.0255114)
Supplement: S1 Data — (ZIP) [file pone.0255114.s002.zip › Open_data_R1/Data_L3_T1.pdf]

Artery wall Layer: Intima (L=3)

Thrombogenic Factor: Fibrin (T=1)

Storage Medium Treatment: No treatment (j=1)

Count of Total Measurements: 113 (n_1=113)

measured values of $y_{k,r,1}^{3,1}$ which is

the percentage of area covered by Fibrin

in the region k of Intima layer from donor r

with No treatment by storage medium

	r=1	r=2	r=3	r=4	r=5	r=6	r=7	r=8	r=9	r=10	r=11
k=1	37.0	26.0	45.0	37.4	27.3	14.1	13.9	2.8	4.8	2.5	8.3
k=2	32.2	20.6	18.5	46.6	16.5	30.8	16.7	5.2	9.8	4.5	8.1
k=3	25.6	29.1	19.3	16.7	21.3	2.5	19.2	4.8	6.3	2.1	4.8
k=4	35.3	20.2	18.0	25.5	41.9	3.9	33.4	7.5	9.6	2.2	11.9
k=5	30.1	N/A	22.7	35.8	41.0	6.1	43.4	12.0	6.0	2.3	8.6
k=6	45.3	N/A	19.2	9.7	24.5	3.4	22.8	39.5	2.6	2.1	2.4
k=7	53.3	N/A	36.4	10.2	N/A	9.0	13.7	22.5	0.6	2.3	33.8
k=8	N/A	N/A	39.5	2.6	N/A	3.4	18.3	27.3	0.5	2.8	26.7
k=9	N/A	N/A	N/A	N/A	N/A	7.6	8.3	14.8	1.0	1.3	22.6

k=10	N/A	N/A	N/A	N/A	N/A	35.8	12.0	19.5	1.5	0.6	22.1
k=11	N/A	N/A	N/A	N/A	N/A	32.8	N/A	36.0	0.4	2.1	5.3
k=12	N/A	N/A	N/A	N/A	N/A	0.9	N/A	36.0	N/A	2.0	N/A
k=13	N/A	N/A	N/A	N/A	N/A	32.3	N/A	N/A	N/A	0.7	N/A
k=14	N/A	N/A	N/A	N/A	N/A	3.8	N/A	N/A	N/A	N/A	N/A
k=15	N/A	N/A	N/A	N/A	N/A	25.0	N/A	N/A	N/A	N/A	N/A
k=16	N/A	N/A	N/A	N/A	N/A	12.8	N/A	N/A	N/A	N/A	N/A
k=17	N/A	N/A	N/A	N/A	N/A	7.4	N/A	N/A	N/A	N/A	N/A
k=18	N/A	N/A	N/A	N/A	N/A	19.4	N/A	N/A	N/A	N/A	N/A
k=19	N/A	N/A	N/A	N/A	N/A	21.8	N/A	N/A	N/A	N/A	N/A
k=20	N/A	N/A	N/A	N/A	N/A	5.4	N/A	N/A	N/A	N/A	N/A
k=21	N/A	N/A	N/A	N/A	N/A	25.7	N/A	N/A	N/A	N/A	N/A
k=22	N/A	N/A	N/A	N/A	N/A	23.6	N/A	N/A	N/A	N/A	N/A
k=23	N/A	N/A	N/A	N/A	N/A	33.9	N/A	N/A	N/A	N/A	N/A

values of $m_{r,1}^{3,1}$ which is

the count of regions covered by Fibrin

in Intima layer from donor *r*
with No treatment by storage medium

r	1	2	3	4	5	6	7	8	9	10	11
count	7	4	8	8	6	23	10	12	11	13	11

Artery wall Layer: Intima (L=3)

Thrombogenic Factor: Fibrin (T=1)

Storage Medium Treatment: 0 weeks (j=2)

Count of Total Measurements: 101 (n_2=101)

measured values of $y_{k,r,2}^{3,1}$ which is

the percentage of area covered by Fibrin

in the region k of Intima layer from donor r

after 0 weeks treatment with storage medium

	r=1	r=2	r=3	r=4	r=5	r=6	r=7	r=8	r=9	r=10	r=11
k=1	22.3	50.7	15.3	29.4	36.8	22.5	18.1	10.6	1.5	29.1	1.9
k=2	42.2	46.2	54.3	0.9	4.7	36.1	64.7	7.0	2.0	0.8	5.8
k=3	49.1	63.6	33.7	2.1	13.6	39.2	12.0	6.5	8.9	2.3	2.0
k=4	24.6	44.1	55.0	54.9	12.2	32.6	6.9	3.8	5.9	1.7	3.2
k=5	23.0	54.8	35.7	32.7	20.8	41.4	24.1	19.8	6.6	0.7	7.2
k=6	37.4	67.3	15.7	33.4	22.5	33.5	31.2	11.9	5.8	12.8	29.6
k=7	27.6	67.1	13.6	15.5	N/A	41.3	47.3	11.4	2.3	N/A	28.1
k=8	32.6	76.0	13.4	0.3	N/A	24.9	57.3	16.7	1.9	N/A	14.6
k=9	N/A	N/A	N/A	N/A	N/A	34.4	51.7	23.2	2.4	N/A	1.7

k=10	N/A	N/A	N/A	N/A	N/A	16.2	N/A	N/A	5.1	N/A	N/A
k=11	N/A	N/A	N/A	N/A	N/A	14.8	N/A	N/A	5.2	N/A	N/A
k=12	N/A	N/A	N/A	N/A	N/A	21.7	N/A	N/A	5.7	N/A	N/A
k=13	N/A	N/A	N/A	N/A	N/A	12.0	N/A	N/A	3.2	N/A	N/A
k=14	N/A	N/A	N/A	N/A	N/A	12.7	N/A	N/A	N/A	N/A	N/A
k=15	N/A	N/A	N/A	N/A	N/A	15.8	N/A	N/A	N/A	N/A	N/A
k=16	N/A	N/A	N/A	N/A	N/A	9.0	N/A	N/A	N/A	N/A	N/A
k=17	N/A	N/A	N/A	N/A	N/A	11.4	N/A	N/A	N/A	N/A	N/A

values of $m_{r,2}^{3,1}$ which is

the count of regions covered by Fibrin

in Intima layer from donor r

after 0 weeks treatment with storage medium

r	1	2	3	4	5	6	7	8	9	10	11
count	8	8	8	8	6	17	9	9	13	6	9

Artery wall Layer: Intima (*L*=3)

Thrombogenic Factor: Fibrin (*T*=1)

Storage Medium Treatment: 1 week (*j*=3)

Count of Total Measurements: 105 (n_3=105)

measured values of $y_{k,r,3}^{3,1}$ which is

the percentage of area covered by Fibrin

in the region *k* of Intima layer from donor *r*

after 1 week treatment with storage medium

	r=1	*r*=2	*r*=3	*r*=4	*r*=5	*r*=6	*r*=7	*r*=8	*r*=9	*r*=10	*r*=11
k=1	30.5	28.5	15.5	0.8	11.4	46.6	6.3	16.6	0.9	15.9	13.8
k=2	21.0	23.0	13.2	0.2	23.7	45.8	29.3	6.9	1.8	22.6	4.5
k=3	31.9	34.2	13.1	1.7	20.7	77.1	69.8	28.4	1.1	29.8	4.9
k=4	26.6	51.7	17.1	0.2	23.7	52.0	16.3	13.3	0.9	13.0	4.5
k=5	48.1	21.9	30.5	8.0	56.3	62.0	32.4	1.2	17.8	10.0	18.2
k=6	33.3	N/A	N/A	2.8	51.6	62.0	15.3	27.1	9.9	11.5	14.2
k=7	24.3	N/A	N/A	2.1	53.6	54.6	4.0	30.0	0.6	25.7	3.5
k=8	27.9	N/A	N/A	0.4	41.9	32.3	N/A	3.2	0.9	23.2	6.6
k=9	25.6	N/A	N/A	N/A	29.8	27.1	N/A	5.2	7.2	26.2	N/A

k=10	40.6	N/A	N/A	N/A	N/A	53.5	N/A	18.7	3.2	37.4	N/A
k=11	N/A	N/A	N/A	N/A	N/A	34.4	N/A	46.3	2.7	25.5	N/A
k=12	N/A	N/A	N/A	N/A	N/A	60.3	N/A	37.0	1.0	N/A	N/A
k=13	N/A	N/A	N/A	N/A	N/A	49.2	N/A	32.0	5.2	N/A	N/A
k=14	N/A	N/A	N/A	N/A	N/A	59.5	N/A	43.1	7.3	N/A	N/A

values of $m_{r,3}^{3,1}$ which is

the count of regions covered by Fibrin

in Intima layer from donor r

after 1 week treatment with storage medium

r	1	2	3	4	5	6	7	8	9	10	11
count	10	5	5	8	9	14	7	14	14	11	8

Artery wall Layer: Intima (L=3)

Thrombogenic Factor: Fibrin (T=1)

Storage Medium Treatment: 12 weeks (j=4)

Count of Total Measurements: 124 (n_4=124)

measured values of $y_{k,r,4}^{3,1}$ which is

the percentage of area covered by Fibrin

in the region k of Intima layer from donor r

after 12 weeks treatment with storage medium

	r=1	r=2	r=3	r=4	r=5	r=6	r=7	r=8	r=9	r=10	r=11
k=1	13.3	29.2	1.3	3.9	19.2	27.8	10.0	13.8	0.1	2.5	8.8
k=2	1.8	21.9	12.5	3.0	13.8	26.6	16.3	10.8	3.4	1.5	16.1
k=3	2.9	32.1	15.5	2.8	5.3	48.6	37.0	21.4	1.0	5.3	12.1
k=4	22.8	27.0	20.5	55.7	11.4	51.3	32.7	0.3	1.1	2.6	8.1
k=5	24.8	33.8	31.9	67.6	12.4	34.9	37.9	2.8	6.5	10.4	11.0
k=6	13.7	27.4	27.2	50.3	19.9	36.7	41.6	8.9	0.0	5.0	16.0
k=7	35.5	49.8	11.3	10.0	33.1	9.8	40.8	0.5	6.8	5.1	10.1
k=8	10.5	25.3	6.8	N/A	35.6	19.2	16.8	2.6	N/A	N/A	3.9
k=9	6.0	58.5	7.6	N/A	44.9	24.1	27.9	2.3	N/A	N/A	2.5

k=10	2.9	23.7	19.9	N/A	36.1	31.6	5.1	N/A	N/A	N/A	9.2
k=11	2.7	14.9	9.9	N/A	32.7	42.4	8.3	N/A	N/A	N/A	6.7
k=12	N/A	15.0	3.9	N/A	N/A	48.9	32.6	N/A	N/A	N/A	8.0
k=13	N/A	2.6	17.6	N/A	N/A	33.6	6.9	N/A	N/A	N/A	13.4
k=14	N/A	N/A	27.0	N/A	N/A	N/A	39.0	N/A	N/A	N/A	6.5
k=15	N/A	N/A	7.5	N/A	N/A	N/A	18.2	N/A	N/A	N/A	2.9
k=16	N/A	N/A	10.8	N/A	N/A	N/A	N/A	N/A	N/A	N/A	N/A

values of $m_{r,4}^{3,1}$ which is

the count of regions covered by Fibrin

in Intima layer from donor r

after 12 weeks treatment with storage medium

r	1	2	3	4	5	6	7	8	9	10	11
count	11	13	16	7	11	13	15	9	7	7	15

Artery wall Layer: Intima (L=3)

Thrombogenic Factor: Fibrin (T=1)

Storage Medium Treatment: 24 weeks (j=5)

Count of Total Measurements: 139 (n_5=139)

measured values of $y_{k,r,5}^{3,1}$ which is

the percentage of area covered by Fibrin

in the region *k* of Intima layer from donor *r*

after 24 weeks treatment with storage medium

	r=1	r=2	r=3	r=4	r=5	r=6	r=7	r=8	r=9	r=10	r=11
k=1	10.8	24.3	24.2	32.3	11.8	28.5	3.7	37.6	0.6	1.7	3.2
k=2	15.6	18.2	13.2	18.0	25.3	20.4	21.7	20.0	5.2	2.1	2.2
k=3	2.4	4.1	14.1	14.4	20.9	8.5	20.0	19.0	1.0	8.8	12.6
k=4	0.8	6.2	16.9	31.5	4.8	11.2	14.4	2.7	0.2	4.4	14.8
k=5	13.7	11.8	27.8	23.3	15.2	21.3	34.2	5.3	0.8	7.2	21.1
k=6	N/A	30.2	18.2	26.7	16.4	15.1	7.2	19.9	2.4	6.8	14.6
k=7	N/A	66.0	9.5	21.8	27.9	29.8	8.4	16.3	5.2	14.3	25.3
k=8	N/A	28.4	23.2	23.6	39.0	15.1	2.0	28.8	7.7	6.2	18.2
k=9	N/A	22.2	14.0	14.8	18.0	5.2	2.9	20.8	3.7	7.8	12.8

k=10	N/A	18.6	13.1	26.2	8.6	8.7	N/A	4.5	3.2	9.4	20.6
k=11	N/A	47.2	11.5	8.8	32.1	4.1	N/A	31.2	2.0	3.0	2.2
k=12	N/A	8.3	12.1	N/A	19.7	6.9	N/A	28.8	0.1	N/A	2.7
k=13	N/A	N/A	8.0	N/A	19.7	12.9	N/A	N/A	N/A	N/A	2.2
k=14	N/A	N/A	N/A	N/A	20.2	8.5	N/A	N/A	N/A	N/A	1.6
k=15	N/A	N/A	N/A	N/A	20.6	5.8	N/A	N/A	N/A	N/A	21.8
k=16	N/A	N/A	N/A	N/A	N/A	28.4	N/A	N/A	N/A	N/A	8.4
k=17	N/A	N/A	N/A	N/A	N/A	9.9	N/A	N/A	N/A	N/A	1.3
k=18	N/A	N/A	N/A	N/A	N/A	N/A	N/A	N/A	N/A	N/A	16.8
k=19	N/A	N/A	N/A	N/A	N/A	N/A	N/A	N/A	N/A	N/A	15.0
k=20	N/A	N/A	N/A	N/A	N/A	N/A	N/A	N/A	N/A	N/A	11.9
k=21	N/A	N/A	N/A	N/A	N/A	N/A	N/A	N/A	N/A	N/A	17.0
k=22	N/A	N/A	N/A	N/A	N/A	N/A	N/A	N/A	N/A	N/A	2.8

values of $m_{r,5}^{3,1}$ which is

the count of regions covered by Fibrin

in Intima layer from donor r

after 24 weeks treatment with storage medium

r	1	2	3	4	5	6	7	8	9	10	11
count	5	12	13	11	15	17	9	12	12	11	22
