# Supplementary material for: Cryopreservation moderates the thrombogenicity of arterial allografts during storage
Source: PLoS One. 2021 Jul 22;16(7):e0255114. doi: 10.1371/journal.pone.0255114 (PMC8297765; doi:10.1371/journal.pone.0255114)
Supplement: S1 Data — (ZIP) [file pone.0255114.s002.zip › Open_data_R1/Data_L3_T2.pdf]

Artery wall Layer: Intima (L=3)

Thrombogenic Factor: Platelet (T=2)

Storage Medium Treatment: No treatment (j=1)

Count of Total Measurements: 114 (n_1=114)

measured values of $y_{k,r,1}^{3,2}$ which is

the percentage of area covered by Platelet

in the region k of Intima layer from donor r

with No treatment by storage medium

	r=1	r=2	r=3	r=4	r=5	r=6	r=7	r=8	r=9	r=10	r=11
k=1	17.6	10.7	29.5	10.5	23.4	14.5	22.2	3.1	0.9	10.6	22.0
k=2	4.8	10.3	3.8	47.5	4.1	17.0	18.4	1.6	4.9	10.2	14.8
k=3	5.9	4.5	5.3	14.7	5.7	13.9	38.8	13.6	3.3	8.1	23.8
k=4	10.1	10.1	6.3	7.6	19.2	11.7	30.2	32.7	12.1	10.8	22.4
k=5	8.7	N/A	6.7	6.5	27.2	10.1	37.9	15.3	8.2	19.6	19.1
k=6	23.3	N/A	6.0	8.9	24.6	4.2	32.8	8.9	11.3	12.0	12.0
k=7	21.4	N/A	19.7	4.5	N/A	7.1	33.5	16.3	4.9	11.1	11.7
k=8	N/A	N/A	17.0	5.0	N/A	12.5	38.9	7.0	4.6	10.6	9.9
k=9	N/A	N/A	N/A	8.1	N/A	11.7	36.1	16.5	10.3	7.4	6.5

k=10	N/A	N/A	N/A	N/A	N/A	7.3	46.2	10.7	1.6	5.3	5.6
k=11	N/A	N/A	N/A	N/A	N/A	7.9	N/A	35.9	9.2	12.5	2.1
k=12	N/A	N/A	N/A	N/A	N/A	2.7	N/A	26.6	N/A	10.1	N/A
k=13	N/A	N/A	N/A	N/A	N/A	6.1	N/A	N/A	N/A	9.0	N/A
k=14	N/A	N/A	N/A	N/A	N/A	5.5	N/A	N/A	N/A	N/A	N/A
k=15	N/A	N/A	N/A	N/A	N/A	3.4	N/A	N/A	N/A	N/A	N/A
k=16	N/A	N/A	N/A	N/A	N/A	8.1	N/A	N/A	N/A	N/A	N/A
k=17	N/A	N/A	N/A	N/A	N/A	4.8	N/A	N/A	N/A	N/A	N/A
k=18	N/A	N/A	N/A	N/A	N/A	1.9	N/A	N/A	N/A	N/A	N/A
k=19	N/A	N/A	N/A	N/A	N/A	1.8	N/A	N/A	N/A	N/A	N/A
k=20	N/A	N/A	N/A	N/A	N/A	1.7	N/A	N/A	N/A	N/A	N/A
k=21	N/A	N/A	N/A	N/A	N/A	14.0	N/A	N/A	N/A	N/A	N/A
k=22	N/A	N/A	N/A	N/A	N/A	9.4	N/A	N/A	N/A	N/A	N/A
k=23	N/A	N/A	N/A	N/A	N/A	11.7	N/A	N/A	N/A	N/A	N/A

values of $m_{r,1}^{3,2}$ which is

the count of regions covered by Platelet

in Intima layer from donor *r*

with No treatment by storage medium

r	1	2	3	4	5	6	7	8	9	10	11
count	7	4	8	9	6	23	10	12	11	13	11

Artery wall Layer: Intima (L=3)

Thrombogenic Factor: Platelet (T=2)

Storage Medium Treatment: 0 weeks (j=2)

Count of Total Measurements: 101 (n_2=101)

measured values of $y_{k,r,2}^{3,2}$ which is

the percentage of area covered by Platelet

in the region k of Intima layer from donor r

after 0 weeks treatment with storage medium

	r=1	r=2	r=3	r=4	r=5	r=6	r=7	r=8	r=9	r=10	r=11
k=1	10.6	0.5	8.4	8.5	9.5	10.6	40.2	13.5	5.3	8.4	42.3
k=2	11.4	0.6	37.6	6.0	2.4	11.8	21.1	10.2	8.7	4.4	34.4
k=3	28.4	4.7	11.5	6.1	9.7	9.6	19.6	17.8	14.2	2.4	25.8
k=4	13.9	1.4	36.1	28.6	15.8	19.8	11.4	19.9	5.7	1.3	26.4
k=5	9.9	9.2	15.5	23.3	33.5	9.9	26.9	23.6	18.2	1.5	36.3
k=6	13.9	10.0	9.7	17.1	23.9	18.6	23.5	16.2	12.0	1.6	19.5
k=7	13.4	7.1	15.9	9.4	N/A	23.7	29.6	16.7	12.7	N/A	22.5
k=8	14.3	26.4	20.1	6.0	N/A	13.1	36.9	31.8	16.2	N/A	19.3
k=9	N/A	N/A	N/A	N/A	N/A	18.3	34.3	10.7	15.8	N/A	16.9

k=10	N/A	N/A	N/A	N/A	N/A	15.2	N/A	N/A	21.4	N/A	N/A
k=11	N/A	N/A	N/A	N/A	N/A	24.4	N/A	N/A	17.4	N/A	N/A
k=12	N/A	N/A	N/A	N/A	N/A	29.9	N/A	N/A	15.2	N/A	N/A
k=13	N/A	N/A	N/A	N/A	N/A	22.0	N/A	N/A	19.1	N/A	N/A
k=14	N/A	N/A	N/A	N/A	N/A	17.1	N/A	N/A	N/A	N/A	N/A
k=15	N/A	N/A	N/A	N/A	N/A	21.6	N/A	N/A	N/A	N/A	N/A
k=16	N/A	N/A	N/A	N/A	N/A	5.8	N/A	N/A	N/A	N/A	N/A
k=17	N/A	N/A	N/A	N/A	N/A	10.7	N/A	N/A	N/A	N/A	N/A

values of $m_{r,2}^{3,2}$ which is

the count of regions covered by Platelet

in Intima layer from donor r

after 0 weeks treatment with storage medium

r	1	2	3	4	5	6	7	8	9	10	11
count	8	8	8	8	6	17	9	9	13	6	9

Artery wall Layer: Intima (*L*=3)

Thrombogenic Factor: Platelet (T=2)

Storage Medium Treatment: 1 week (*j*=3)

Count of Total Measurements: 106 (n_3=106)

measured values of $y^{3,2}_{k,r,3}$ which is

the percentage of area covered by Platelet

in the region *k* of Intima layer from donor *r*

after 1 week treatment with storage medium

	r=1	*r*=2	*r*=3	*r*=4	*r*=5	*r*=6	*r*=7	*r*=8	*r*=9	*r*=10	*r*=11
k=1	15.0	12.2	11.7	12.2	34.4	27.3	8.4	39.5	7.4	16.0	3.3
k=2	5.2	8.4	12.9	4.5	23.5	39.0	18.2	16.5	9.2	13.8	4.7
k=3	4.3	4.9	9.0	4.1	26.4	40.0	12.0	7.0	10.0	19.8	7.5
k=4	5.0	12.3	17.3	3.2	18.8	33.4	15.9	11.1	7.0	12.7	4.1
k=5	5.5	5.7	38.8	6.8	54.9	49.4	22.2	24.2	24.1	12.2	10.6
k=6	5.7	N/A	N/A	16.3	51.1	49.4	18.3	44.5	19.1	12.4	7.1
k=7	4.2	N/A	N/A	5.8	65.3	24.9	15.1	33.3	4.8	19.4	4.3
k=8	11.6	N/A	N/A	4.3	67.2	16.4	N/A	12.2	6.9	13.7	7.6
k=9	7.4	N/A	N/A	8.7	61.8	12.9	N/A	21.9	17.8	13.6	N/A

k=10	20.8	N/A	N/A	N/A	N/A	30.6	N/A	26.1	16.2	13.0	N/A
k=11	N/A	N/A	N/A	N/A	N/A	26.7	N/A	24.2	15.1	13.6	N/A
k=12	N/A	N/A	N/A	N/A	N/A	53.4	N/A	26.1	4.3	N/A	N/A
k=13	N/A	N/A	N/A	N/A	N/A	64.7	N/A	13.4	16.2	N/A	N/A
k=14	N/A	N/A	N/A	N/A	N/A	68.4	N/A	20.3	17.7	N/A	N/A

values of $m_{r,3}^{3,2}$ which is

the count of regions covered by Platelet

in Intima layer from donor r

after 1 week treatment with storage medium

r	1	2	3	4	5	6	7	8	9	10	11
count	10	5	5	9	9	14	7	14	14	11	8

Artery wall Layer: Intima (L=3)

Thrombogenic Factor: Platelet (T=2)

Storage Medium Treatment: 12 weeks (j=4)

Count of Total Measurements: 125 (n_4=125)

measured values of $y_{k,r,4}^{3,2}$ which is

the percentage of area covered by Platelet

in the region k of Intima layer from donor r

after 12 weeks treatment with storage medium

	r=1	r=2	r=3	r=4	r=5	r=6	r=7	r=8	r=9	r=10	r=11
k=1	19.7	38.7	2.8	2.9	17.5	17.2	26.1	14.3	17.5	4.3	17.5
k=2	3.0	18.6	7.6	2.1	21.6	25.9	26.1	9.1	7.4	2.9	17.4
k=3	14.6	44.6	13.3	2.1	30.3	11.9	41.1	17.5	2.8	7.5	15.5
k=4	18.9	25.6	10.2	3.6	30.7	10.7	42.6	1.2	1.4	7.3	24.9
k=5	12.6	12.9	24.7	18.5	27.9	4.5	33.8	15.1	6.4	17.2	15.5
k=6	14.7	49.3	16.5	12.3	43.7	12.9	32.3	10.5	4.9	3.6	19.2
k=7	34.1	37.9	13.7	13.1	23.2	10.3	38.3	13.4	3.4	3.7	11.8
k=8	13.2	32.6	8.6	N/A	11.6	7.0	31.0	21.6	N/A	3.8	18.4
k=9	6.6	49.4	19.8	N/A	17.7	11.2	44.6	19.0	N/A	N/A	4.2

k=10	7.8	33.0	50.6	N/A	17.0	10.8	25.9	N/A	N/A	N/A	18.9
k=11	12.5	31.0	55.1	N/A	18.5	15.3	22.8	N/A	N/A	N/A	10.9
k=12	N/A	24.9	20.7	N/A	N/A	31.4	44.0	N/A	N/A	N/A	14.9
k=13	N/A	37.2	48.8	N/A	N/A	23.5	32.8	N/A	N/A	N/A	15.1
k=14	N/A	N/A	41.2	N/A	N/A	N/A	31.8	N/A	N/A	N/A	22.6
k=15	N/A	N/A	30.3	N/A	N/A	N/A	24.1	N/A	N/A	N/A	12.1
k=16	N/A	N/A	19.0	N/A	N/A	N/A	N/A	N/A	N/A	N/A	N/A

values of $m_{r,4}^{3,2}$ which is

the count of regions covered by Platelet

in Intima layer from donor *r*

after 12 weeks treatment with storage medium

r	1	2	3	4	5	6	7	8	9	10	11
count	11	13	16	7	11	13	15	9	7	8	15

Artery wall Layer: Intima (L=3)

Thrombogenic Factor: Platelet (T=2)

Storage Medium Treatment: 24 weeks (j=5)

Count of Total Measurements: 139 (n_5=139)

measured values of $y_{k,r,5}^{3,2}$ which is

the percentage of area covered by Platelet

in the region k of Intima layer from donor r

after 24 weeks treatment with storage medium

	r=1	r=2	r=3	r=4	r=5	r=6	r=7	r=8	r=9	r=10	r=11
k=1	9.7	18.0	25.7	13.1	19.4	16.4	2.9	2.9	9.2	14.5	15.6
k=2	14.8	23.3	27.7	4.1	32.6	1.4	7.7	0.3	11.9	10.1	12.6
k=3	13.1	21.7	24.2	2.4	25.8	0.2	11.3	0.3	13.5	17.0	14.9
k=4	7.8	18.9	19.5	19.3	13.6	0.6	2.2	2.4	15.9	14.1	14.6
k=5	19.7	9.1	20.2	16.0	29.9	4.3	6.9	3.2	8.5	7.4	9.4
k=6	N/A	34.3	28.4	13.9	24.1	9.6	6.6	6.1	14.0	9.2	15.7
k=7	N/A	48.7	8.1	10.2	36.7	5.1	4.9	5.3	6.0	11.6	19.8
k=8	N/A	19.6	15.6	7.5	7.1	18.5	2.6	0.5	13.8	9.8	14.8
k=9	N/A	7.1	10.7	6.9	6.9	14.1	1.5	0.4	9.3	2.3	11.4

k=10	N/A	6.6	13.5	14.7	27.4	30.1	N/A	0.6	10.0	7.5	19.9
k=11	N/A	28.8	11.5	6.3	44.2	30.8	N/A	1.5	12.2	9.6	4.7
k=12	N/A	4.3	21.4	N/A	23.0	21.1	N/A	4.3	6.6	N/A	12.9
k=13	N/A	N/A	35.9	N/A	19.2	15.6	N/A	N/A	N/A	N/A	10.4
k=14	N/A	N/A	N/A	N/A	15.7	2.0	N/A	N/A	N/A	N/A	11.2
k=15	N/A	N/A	N/A	N/A	12.9	1.3	N/A	N/A	N/A	N/A	20.4
k=16	N/A	N/A	N/A	N/A	N/A	2.0	N/A	N/A	N/A	N/A	4.8
k=17	N/A	N/A	N/A	N/A	N/A	2.9	N/A	N/A	N/A	N/A	9.1
k=18	N/A	N/A	N/A	N/A	N/A	N/A	N/A	N/A	N/A	N/A	14.9
k=19	N/A	N/A	N/A	N/A	N/A	N/A	N/A	N/A	N/A	N/A	9.6
k=20	N/A	N/A	N/A	N/A	N/A	N/A	N/A	N/A	N/A	N/A	10.6
k=21	N/A	N/A	N/A	N/A	N/A	N/A	N/A	N/A	N/A	N/A	10.3
k=22	N/A	N/A	N/A	N/A	N/A	N/A	N/A	N/A	N/A	N/A	22.0

values of $m_{r,5}^{3,2}$ which is

the count of regions covered by Platelet

in Intima layer from donor r

after 24 weeks treatment with storage medium

r	1	2	3	4	5	6	7	8	9	10	11
count	5	12	13	11	15	17	9	12	12	11	22
