# Supplementary material for: Cryopreservation moderates the thrombogenicity of arterial allografts during storage
Source: PLoS One. 2021 Jul 22;16(7):e0255114. doi: 10.1371/journal.pone.0255114 (PMC8297765; doi:10.1371/journal.pone.0255114)
Supplement: S1 Data — (ZIP) [file pone.0255114.s002.zip › Open_data_R1/Fuzzy_statistics_regression_R1.pdf]

Data for the regression analysis of the temporal trends in thrombogenicity of the grafts

For each combination of layer and substance (six in total), we construct three linear regression models: for the lower quantile (LQR), for the median (MED) and for the upper quartile (UQR).

The slope coefficient of each of the 18 regressions is considered significantly negative when at least two of the four fuzzy Bootstrap procedures, which form the cluster $Cl_{sign,k}$ (described in Supporting_information1.pdf), determine that the probability for non-negativity of the slope is less or equal t0 0.05. In that case, the adjusted R-square of the model is highlighted in yellow in the analytical datasets below. If the adjusted R-square is highlighted in red, it means that the cluster $Cl_{sign,k}$ cannot detect enough information to declare the slope of the regression significant. For the sake of comparison, we have calculated the analytical t-test p-value for the same regression. Again, if the analytical p-value is less or equal to 0.05, we highlight it in yellow to demonstrate that according to the analytical t-test the slope of the regression is significantly negative. In the same way, if the analytical p-value is greater than 0.05, we highlight it in red to demonstrate that according to the analytical t-test there is not enough information to declare the slope of the regression negative.

Out of the 18 models, both the cluster method and the analytical method indicate significantly negative slope in 3 models; the cluster method and the analytical method indicate insignificant slope in 4 models; in the remaining 11 models the cluster method identifies significance, while the analytical method fails to identify any significant slope. The results are well expected since the analytical regressions are constructed on 4 points of central tendency with a consequent very low statistical power of the method, whereas the fuzzy sample approach operates with raw data weighted by their degree of membership and its robustness is further enhanced by the parallel application of four different Bootstrap tests. The comparison of the regression analysis results illustrates the advantage of using the cluster method to identify significance of regression coefficients.

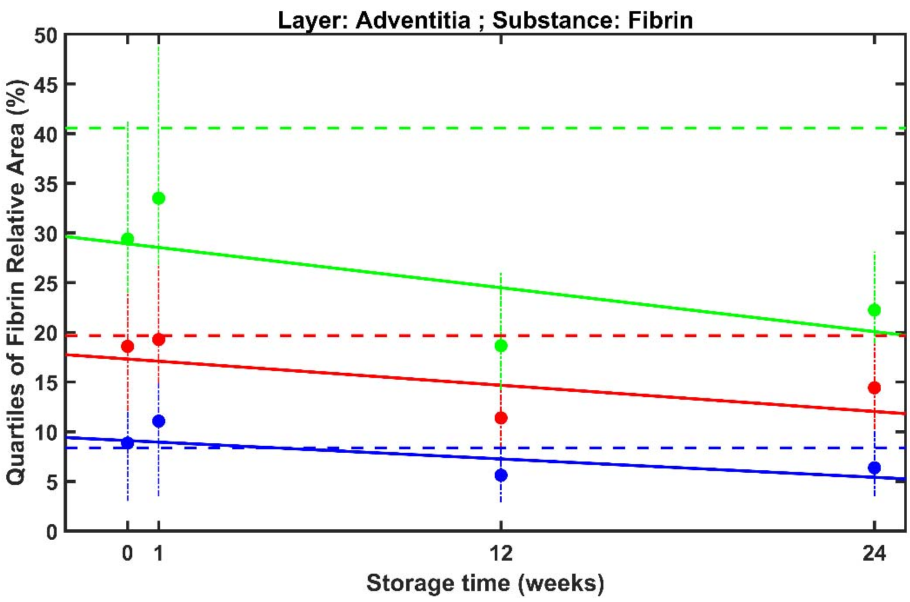

Layer: Adventitia
Substance: Fibrin
Regressand: Lower Quartile

Model
'25%-quantile'=b0+(b1)x'Treatment_time'+eps
b0: 8.572e+00%
analytical 95% confidence interval: 1.453e+00<b0<1.569e+01
b1: -1.335e-01%/week
analytical 95% confidence interval: -6.008e-01<b1<3.338e-01
standard deviation of eps (sigma): 1.911e+00%
analytical 95% confidence interval: 9.952e-01<sigma<1.201e+01
R-square: 5.779e-01
adjusted R-square: 3.669e-01

Hypothesis Testing
H0: 'b1 is equal to 0%/week'
H1: 'b1 is different from 0%/week'
analytical t-test p-value: 3.439e-01
fuzzy Bootstrap Probability for b1 to be non-negative calculated with 10000 pseudo-realities:
quasi-equal-information generation from ECDF: 1.490e-02
quasi-equal-information generation from FECDF: 3.410e-02
equal-size generation from ECDF: 1.640e-02
equal-size generation from FECDF: 3.190e-02

fuzzy Bootstrap 95% confidence intervals estimated with 10000 pseudo-realities:
quasi-equal-information generation from ECDF:
6.806e+00<b0<1.032e+01
-2.383e-01<b1<-1.328e-02
8.255e-01<sigma<3.354e+00
quasi-equal-information generation from FECDF:
6.858e+00<b0<1.150e+01
-2.702e-01<b1<9.487e-03
6.334e-01<sigma<3.535e+00

equal-size generation from ECDF:
6.778e+00<b0<1.035e+01
-2.353e-01<b1<-1.258e-02
8.365e-01<sigma<3.331e+00
equal-size generation from FECDF:
6.806e+00<b0<1.134e+01
-2.597e-01<b1<7.577e-03
6.719e-01<sigma<3.344e+00

Layer: Adventitia
Substance: Fibrin
Regressand: Median

Model
'50%-quantile'=b0+(b1)x'Treatment_time'+eps
b0: 1.627e+01%
analytical 95% confidence interval: 1.823e+00<b0<3.071e+01
b1: -1.930e-01%/week
analytical 95% confidence interval: -1.191e+00<b1<8.049e-01
standard deviation of eps (sigma): 3.784e+00%
analytical 95% confidence interval: 1.970e+00<sigma<2.378e+01
R-square: 4.674e-01
adjusted R-square: 2.010e-01

Hypothesis Testing
H0: 'b1 is equal to 0%/week'
H1: 'b1 is different from 0%/week'
analytical t-test p-value: 4.929e-01
fuzzy Bootstrap Probability for b1 to be non-negative calculated with 10000 pseudo-realities:
quasi-equal-information generation from ECDF: 1.830e-02
quasi-equal-information generation from FECDF: 1.300e-03
equal-size generation from ECDF: 2.040e-02
equal-size generation from FECDF: 6.000e-04

fuzzy Bootstrap 95% confidence intervals estimated with 10000 pseudo-realities:
quasi-equal-information generation from ECDF:
1.378e+01<b0<1.883e+01
-3.595e-01<b1<-1.207e-02
2.440e+00<sigma<5.536e+00
quasi-equal-information generation from FECDF:
1.608e+01<b0<2.409e+01
-6.336e-01<b1<-1.315e-01
3.640e+00<sigma<6.724e+00
equal-size generation from ECDF:
1.375e+01<b0<1.875e+01
-3.597e-01<b1<-8.602e-03
2.453e+00<sigma<5.548e+00
equal-size generation from FECDF:
1.630e+01<b0<2.393e+01
-6.176e-01<b1<-1.509e-01
3.766e+00<sigma<6.727e+00

Layer: Adventitia
Substance: Fibrin
Regressand: Upper Quartile

Model
'75%-quantile'=b0+(b1)x'Treatment_time'+eps
b0: 2.747e+01%
analytical 95% confidence interval: 6.244e+00<b0<4.870e+01
b1: -2.838e-01%/week
analytical 95% confidence interval: -1.469e+00<b1<9.015e-01
standard deviation of eps (sigma): 5.228e+00%
analytical 95% confidence interval: 2.722e+00<sigma<3.286e+01
R-square: 5.234e-01
adjusted R-square: 2.850e-01

Hypothesis Testing
H0: 'b1 is equal to 0%/week'
H1: 'b1 is different from 0%/week'
analytical t-test p-value: 4.112e-01
fuzzy Bootstrap Probability for b1 to be non-negative calculated with 10000 pseudo-realities:
quasi-equal-information generation from ECDF: 2.000e-04
quasi-equal-information generation from FECDF: 1.000e-04
equal-size generation from ECDF: 1.000e-04
equal-size generation from FECDF: 1.000e-04

fuzzy Bootstrap 95% confidence intervals estimated with 10000 pseudo-realities:
quasi-equal-information generation from ECDF:
 2.429e+01<b0<3.225e+01
 -5.181e-01<b1<-1.407e-01
 2.857e+00<sigma<7.937e+00
quasi-equal-information generation from FECDF:
 2.558e+01<b0<3.395e+01
 -6.057e-01<b1<-1.932e-01
 2.502e+00<sigma<7.683e+00
equal-size generation from ECDF:
 2.433e+01<b0<3.224e+01
 -5.196e-01<b1<-1.415e-01
 2.830e+00<sigma<7.979e+00
equal-size generation from FECDF:
 2.572e+01<b0<3.361e+01
 -5.898e-01<b1<-2.010e-01
 2.615e+00<sigma<7.584e+00

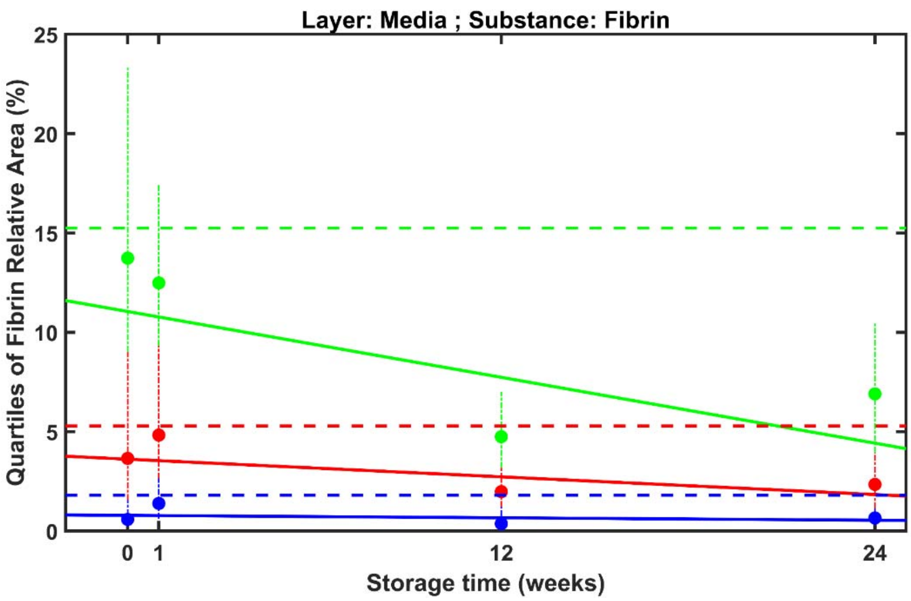

Layer: Media
Substance: Fibrin
Regressand: Lower Quartile

Model
'25%-quantile'=b0+(b1)x'Treatment_time'+eps
b0: 6.806e-01%
analytical 95% confidence interval: -7.027e-01<b0<2.064e+00
b1: -4.536e-03%/week
analytical 95% confidence interval: -8.557e-02<b1<7.650e-02
standard deviation of eps (sigma): 3.666e-01%
analytical 95% confidence interval: 1.909e-01<sigma<2.304e+00
R-square: 2.227e-01
adjusted R-square: -1.659e-01

Hypothesis Testing
H0: 'b1 is equal to 0%/week'
H1: 'b1 is different from 0%/week'
analytical t-test p-value: 8.321e-01
fuzzy Bootstrap Probability for b1 to be non-negative calculated with 10000 pseudo-realities:
quasi-equal-information generation from ECDF: 2.095e-01
quasi-equal-information generation from FECDF: 2.200e-01
equal-size generation from ECDF: 2.044e-01
equal-size generation from FECDF: 2.079e-01

fuzzy Bootstrap 95% confidence intervals estimated with 10000 pseudo-realities:
quasi-equal-information generation from ECDF:
 3.691e-01<b0<1.007e+00
 -2.248e-02<b1<7.881e-03
 1.969e-01<sigma<5.174e-01
quasi-equal-information generation from FECDF:
 3.315e-01<b0<1.177e+00
 -2.615e-02<b1<1.048e-02
 2.712e-01<sigma<7.061e-01
equal-size generation from ECDF:

3.675e-01<b0<1.001e+00
-2.197e-02<b1<7.810e-03
2.009e-01<sigma<5.228e-01
equal-size generation from FECDF:
3.349e-01<b0<1.163e+00
-2.552e-02<b1<1.031e-02
2.775e-01<sigma<6.923e-01

Layer: Media
Substance: Fibrin
Regressand: Median

Model
'50%-quantile'=b0+(b1)x'Treatment_time'+eps
b0: 2.897e+00%
analytical 95% confidence interval: -1.490e+00<b0<7.284e+00
b1: -4.062e-02%/week
analytical 95% confidence interval: -3.129e-01<b1<2.316e-01
standard deviation of eps (sigma): 8.870e-01%
analytical 95% confidence interval: 4.618e-01<sigma<5.575e+00
R-square: 6.911e-01
adjusted R-square: 5.366e-01

Hypothesis Testing
H0: 'b1 is equal to 0%/week'
H1: 'b1 is different from 0%/week'
analytical t-test p-value: 5.867e-01
fuzzy Bootstrap Probability for b1 to be non-negative calculated with 10000 pseudo-realities:
quasi-equal-information generation from ECDF: 2.020e-02
quasi-equal-information generation from FECDF: 3.180e-02
equal-size generation from ECDF: 1.940e-02
equal-size generation from FECDF: 2.600e-02

fuzzy Bootstrap 95% confidence intervals estimated with 10000 pseudo-realities:
quasi-equal-information generation from ECDF:
2.273e+00<b0<4.177e+00
-1.092e-01<b1<-1.970e-03
3.983e-01<sigma<1.672e+00
quasi-equal-information generation from FECDF:
2.391e+00<b0<5.308e+00
-1.667e-01<b1<4.078e-03
8.223e-01<sigma<2.606e+00
equal-size generation from ECDF:
2.263e+00<b0<4.173e+00
-1.084e-01<b1<-2.791e-03
3.983e-01<sigma<1.646e+00
equal-size generation from FECDF:
2.410e+00<b0<5.262e+00
-1.608e-01<b1<5.391e-04
8.442e-01<sigma<2.620e+00

Layer: Media
Substance: Fibrin
Regressand: Upper Quartile

Model
'75%-quantile'=b0+(b1)x'Treatment_time'+eps
b0: 9.436e+00%
analytical 95% confidence interval: -5.897e+00<b0<2.477e+01
b1: -2.231e-01%/week
analytical 95% confidence interval: -1.283e+00<b1<8.364e-01
standard deviation of eps (sigma): 3.580e+00%
analytical 95% confidence interval: 1.864e+00<sigma<2.250e+01
R-square: 6.232e-01
adjusted R-square: 4.347e-01

Hypothesis Testing
H0: 'b1 is equal to 0%/week'
H1: 'b1 is different from 0%/week'
analytical t-test p-value: 4.606e-01
fuzzy Bootstrap Probability for b1 to be non-negative calculated with 10000 pseudo-realities:
quasi-equal-information generation from ECDF: 2.100e-03
quasi-equal-information generation from FECDF: 1.300e-03
equal-size generation from ECDF: 2.200e-03
equal-size generation from FECDF: 4.000e-04

fuzzy Bootstrap 95% confidence intervals estimated with 10000 pseudo-realities:
quasi-equal-information generation from ECDF:
 6.981e+00<b0<1.210e+01
 -3.753e-01<b1<-6.237e-02
 2.487e+00<sigma<4.859e+00
quasi-equal-information generation from FECDF:
 7.799e+00<b0<1.381e+01
 -4.934e-01<b1<-9.491e-02
 3.407e+00<sigma<6.008e+00
equal-size generation from ECDF:
 7.012e+00<b0<1.214e+01
 -3.788e-01<b1<-6.903e-02
 2.484e+00<sigma<4.900e+00
equal-size generation from FECDF:
 7.849e+00<b0<1.368e+01
 -4.786e-01<b1<-9.841e-02
 3.457e+00<sigma<5.978e+00

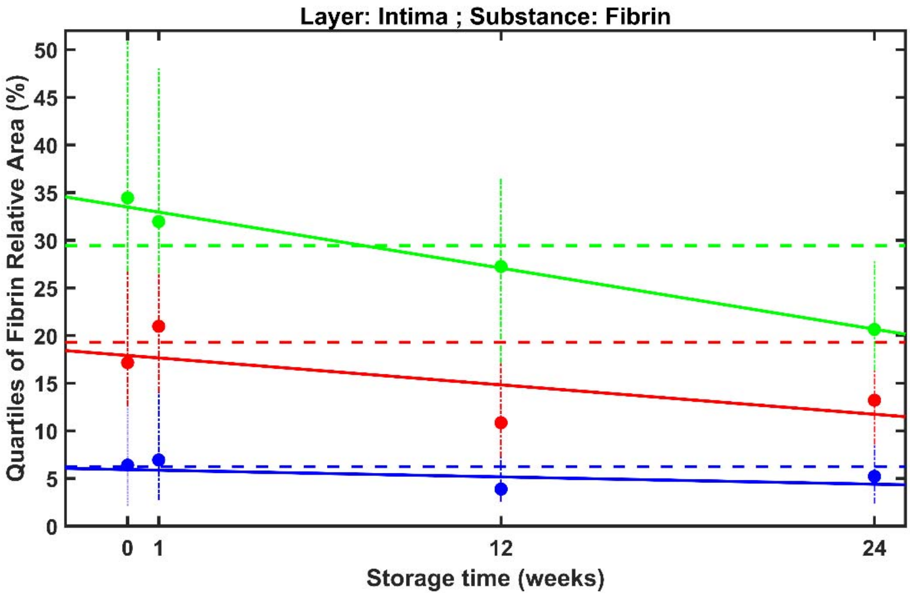

Layer: Intima
Substance: Fibrin
Regressand: Lower Quartile

Model
'25%-quantile'=b0+(b1)x'Treatment_time'+eps
b0: 5.335e+00%
analytical 95% confidence interval: -6.300e-01<b0<1.130e+01
b1: -3.926e-02%/week
analytical 95% confidence interval: -4.210e-01<b1<3.425e-01
standard deviation of eps (sigma): 1.445e+00%
analytical 95% confidence interval: 7.525e-01<sigma<9.083e+00
R-square: 4.164e-01
adjusted R-square: 1.245e-01

Hypothesis Testing
H0: 'b1 is equal to 0%/week'
H1: 'b1 is different from 0%/week'
analytical t-test p-value: 7.014e-01
fuzzy Bootstrap Probability for b1 to be non-negative calculated with 10000 pseudo-realities:
quasi-equal-information generation from ECDF: 2.803e-01
quasi-equal-information generation from FECDF: 1.535e-01
equal-size generation from ECDF: 2.837e-01
equal-size generation from FECDF: 1.373e-01

fuzzy Bootstrap 95% confidence intervals estimated with 10000 pseudo-realities:
quasi-equal-information generation from ECDF:
3.089e+00<b0<8.882e+00
-2.159e-01<b1<1.008e-01
3.020e-01<sigma<3.719e+00
quasi-equal-information generation from FECDF:
2.708e+00<b0<8.568e+00
-2.790e-01<b1<7.978e-02
6.941e-01<sigma<4.094e+00

equal-size generation from ECDF:
3.146e+00<b0<8.864e+00
-2.175e-01<b1<9.939e-02
3.247e-01<sigma<3.661e+00
equal-size generation from FECDF:
2.835e+00<b0<8.476e+00
-2.695e-01<b1<6.739e-02
7.428e-01<sigma<4.056e+00

Layer: Intima
Substance: Fibrin
Regressand: Median

Model
'50%-quantile'=b0+(b1)x'Treatment_time'+eps
b0: 1.755e+01%
analytical 95% confidence interval: 3.710e+00<b0<3.140e+01
b1: -2.291e-01%/week
analytical 95% confidence interval: -1.042e+00<b1<5.841e-01
standard deviation of eps (sigma): 3.745e+00%
analytical 95% confidence interval: 1.950e+00<sigma<2.354e+01
R-square: 4.817e-01
adjusted R-square: 2.225e-01

Hypothesis Testing
H0: 'b1 is equal to 0%/week'
H1: 'b1 is different from 0%/week'
analytical t-test p-value: 3.492e-01
fuzzy Bootstrap Probability for b1 to be non-negative calculated with 10000 pseudo-realities:
quasi-equal-information generation from ECDF: 7.200e-03
quasi-equal-information generation from FECDF: 9.800e-03
equal-size generation from ECDF: 7.700e-03
equal-size generation from FECDF: 6.800e-03

fuzzy Bootstrap 95% confidence intervals estimated with 10000 pseudo-realities:
quasi-equal-information generation from ECDF:
1.379e+01<b0<2.193e+01
-4.667e-01<b1<-4.169e-02
9.964e-01<sigma<6.050e+00
quasi-equal-information generation from FECDF:
1.255e+01<b0<2.117e+01
-4.916e-01<b1<-3.072e-02
1.842e+00<sigma<7.367e+00
equal-size generation from ECDF:
1.380e+01<b0<2.197e+01
-4.666e-01<b1<-3.669e-02
1.020e+00<sigma<6.063e+00
equal-size generation from FECDF:
1.265e+01<b0<2.100e+01
-4.797e-01<b1<-4.333e-02
1.904e+00<sigma<7.275e+00

Layer: Intima
Substance: Fibrin
Regressand: Upper Quartile

Model
'75%-quantile'=b0+(b1)x'Treatment_time'+eps
b0: 3.345e+01%
analytical 95% confidence interval: 3.060e+01<b0<3.629e+01
b1: -5.326e-01%/week
analytical 95% confidence interval: -6.878e-01<b1<-3.775e-01
standard deviation of eps (sigma): 7.303e-01%
analytical 95% confidence interval: 3.802e-01<sigma<4.590e+00
R-square: 9.934e-01
adjusted R-square: 9.900e-01

Hypothesis Testing
H0: 'b1 is equal to 0%/week'
H1: 'b1 is different from 0%/week'
analytical t-test p-value: 4.552e-03
fuzzy Bootstrap Probability for b1 to be non-negative calculated with 10000 pseudo-realities:
quasi-equal-information generation from ECDF: 0.000e+00
quasi-equal-information generation from FECDF: 0.000e+00
equal-size generation from ECDF: 0.000e+00
equal-size generation from FECDF: 0.000e+00

fuzzy Bootstrap 95% confidence intervals estimated with 10000 pseudo-realities:
quasi-equal-information generation from ECDF:
2.968e+01<b0<3.879e+01
-7.785e-01<b1<-3.380e-01
3.190e-01<sigma<5.235e+00
quasi-equal-information generation from FECDF:
2.874e+01<b0<3.766e+01
-7.846e-01<b1<-3.412e-01
4.437e-01<sigma<6.205e+00
equal-size generation from ECDF:
2.962e+01<b0<3.896e+01
-7.813e-01<b1<-3.396e-01
3.145e-01<sigma<5.220e+00
equal-size generation from FECDF:
2.891e+01<b0<3.730e+01
-7.713e-01<b1<-3.552e-01
4.236e-01<sigma<6.040e+00

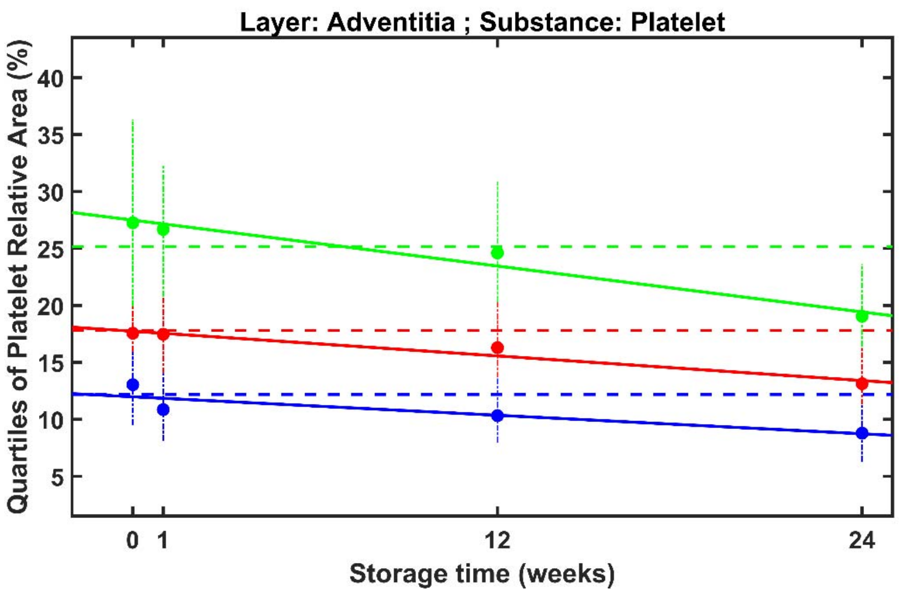

Layer: Adventitia
Substance: Platelet
Regressand: Lower Quartile

Model
'25%-quantile'=b0+(b1)x'Treatment_time'+eps
b0: 1.194e+01%
analytical 95% confidence interval: 8.924e+00<b0<1.495e+01
b1: -1.332e-01%/week
analytical 95% confidence interval: -3.354e-01<b1<6.906e-02
standard deviation of eps (sigma): 9.480e-01%
analytical 95% confidence interval: 4.936e-01<sigma<5.958e+00
R-square: 8.067e-01
adjusted R-square: 7.100e-01

Hypothesis Testing
H0: 'b1 is equal to 0%/week'
H1: 'b1 is different from 0%/week'
analytical t-test p-value: 1.053e-01
fuzzy Bootstrap Probability for b1 to be non-negative calculated with 10000 pseudo-realities:
quasi-equal-information generation from ECDF: 1.000e-03
quasi-equal-information generation from FECDF: 1.700e-03
equal-size generation from ECDF: 1.700e-03
equal-size generation from FECDF: 1.500e-03

fuzzy Bootstrap 95% confidence intervals estimated with 10000 pseudo-realities:
quasi-equal-information generation from ECDF:
1.055e+01<b0<1.326e+01
-2.026e-01<b1<-4.320e-02
1.512e-01<sigma<2.103e+00
quasi-equal-information generation from FECDF:
1.090e+01<b0<1.420e+01
-2.212e-01<b1<-4.435e-02
9.025e-01<sigma<3.075e+00

equal-size generation from ECDF:
  1.055e+01<b0<1.325e+01
  -2.021e-01<b1<-4.218e-02
  1.458e-01<sigma<2.074e+00
equal-size generation from FECDF:
  1.096e+01<b0<1.407e+01
  -2.144e-01<b1<-4.706e-02
  9.626e-01<sigma<3.032e+00

Layer: Adventitia
Substance: Platelet
Regressand: Median

Model
'50%-quantile'=b0+(b1)x'Treatment_time'+eps
b0: 1.768e+01%
analytical 95% confidence interval: 1.637e+01<b0<1.900e+01
b1: -1.805e-01%/week
analytical 95% confidence interval: -2.806e-01<b1<-8.044e-02
standard deviation of eps (sigma): 4.756e-01%
analytical 95% confidence interval: 2.476e-01<sigma<2.989e+00
R-square: 9.679e-01
adjusted R-square: 9.519e-01

Hypothesis Testing
H0: 'b1 is equal to 0%/week'
H1: 'b1 is different from 0%/week'
analytical t-test p-value: 1.620e-02
fuzzy Bootstrap Probability for b1 to be non-negative calculated with 10000 pseudo-realities:
quasi-equal-information generation from ECDF: 1.000e-04
quasi-equal-information generation from FECDF: 3.000e-04
equal-size generation from ECDF: 0.000e+00
equal-size generation from FECDF: 6.000e-04

fuzzy Bootstrap 95% confidence intervals estimated with 10000 pseudo-realities:
quasi-equal-information generation from ECDF:
  1.662e+01<b0<1.901e+01
  -2.513e-01<b1<-8.979e-02
  1.795e-01<sigma<1.846e+00
quasi-equal-information generation from FECDF:
  1.659e+01<b0<1.913e+01
  -2.504e-01<b1<-7.159e-02
  2.571e-01<sigma<2.282e+00
equal-size generation from ECDF:
  1.663e+01<b0<1.903e+01
  -2.501e-01<b1<-9.003e-02
  1.769e-01<sigma<1.859e+00
equal-size generation from FECDF:
  1.665e+01<b0<1.908e+01
  -2.485e-01<b1<-7.961e-02
  2.388e-01<sigma<2.252e+00

Layer: Adventitia
Substance: Platelet
Regressand: Upper Quartile

Model
'75%-quantile'=b0+(b1)x'Treatment_time'+eps
b0: 2.757e+01%
analytical 95% confidence interval: 2.412e+01<b0<3.102e+01
b1: -3.443e-01%/week
analytical 95% confidence interval: -5.393e-01<b1<-1.492e-01
standard deviation of eps (sigma): 9.070e-01%
analytical 95% confidence interval: 4.723e-01<sigma<5.700e+00
R-square: 9.737e-01
adjusted R-square: 9.606e-01

Hypothesis Testing
H0: 'b1 is equal to 0%/week'
H1: 'b1 is different from 0%/week'
analytical t-test p-value: 1.690e-02
fuzzy Bootstrap Probability for b1 to be non-negative calculated with 10000 pseudo-realities:
quasi-equal-information generation from ECDF: 0.000e+00
quasi-equal-information generation from FECDF: 0.000e+00
equal-size generation from ECDF: 0.000e+00
equal-size generation from FECDF: 0.000e+00

fuzzy Bootstrap 95% confidence intervals estimated with 10000 pseudo-realities:
quasi-equal-information generation from ECDF:
2.526e+01<b0<2.917e+01
-4.492e-01<b1<-2.158e-01
2.553e-01<sigma<2.722e+00
quasi-equal-information generation from FECDF:
2.497e+01<b0<2.988e+01
-4.796e-01<b1<-2.204e-01
3.344e-01<sigma<3.168e+00
equal-size generation from ECDF:
2.524e+01<b0<2.919e+01
-4.512e-01<b1<-2.187e-01
2.618e-01<sigma<2.708e+00
equal-size generation from FECDF:
2.522e+01<b0<2.974e+01
-4.741e-01<b1<-2.313e-01
3.331e-01<sigma<3.038e+00

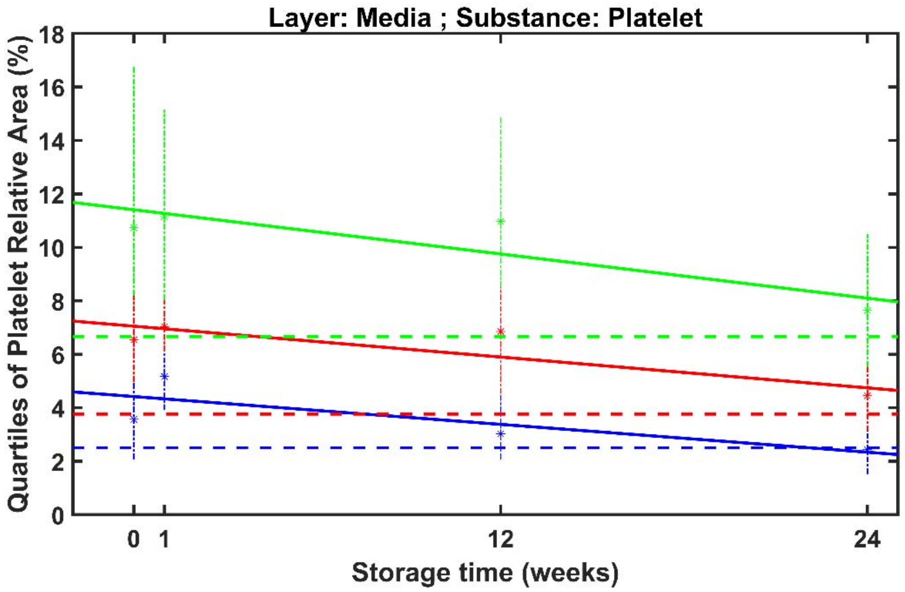

Layer: Media
Substance: Platelet
Regressand: Lower Quartile

Model
'25%-quantile'=b0+(b1)x'Treatment_time'+eps
b0: 4.519e+00%
analytical 95% confidence interval: 1.918e+00<b0<7.120e+00
b1: -9.010e-02%/week
analytical 95% confidence interval: -2.456e-01<b1<6.535e-02
standard deviation of eps (sigma): 7.588e-01%
analytical 95% confidence interval: 3.951e-01<sigma<4.769e+00
R-square: 7.649e-01
adjusted R-square: 6.473e-01

Hypothesis Testing
H0: 'b1 is equal to 0%/week'
H1: 'b1 is different from 0%/week'
analytical t-test p-value: 1.301e-01
fuzzy Bootstrap Probability for b1 to be non-negative calculated with 10000 pseudo-realities:
quasi-equal-information generation from ECDF: 0.000e+00
quasi-equal-information generation from FECDF: 0.000e+00
equal-size generation from ECDF: 0.000e+00
equal-size generation from FECDF: 0.000e+00

fuzzy Bootstrap 95% confidence intervals estimated with 10000 pseudo-realities:
quasi-equal-information generation from ECDF:
3.904e+00<b0<5.004e+00
-1.154e-01<b1<-5.957e-02
3.796e-01<sigma<1.140e+00
quasi-equal-information generation from FECDF:
4.334e+00<b0<5.508e+00
-1.367e-01<b1<-8.014e-02
2.399e-01<sigma<1.001e+00

equal-size generation from ECDF:
3.899e+00<b0<5.021e+00
-1.157e-01<b1<-5.904e-02
3.793e-01<sigma<1.163e+00
equal-size generation from FECDF:
4.353e+00<b0<5.440e+00
-1.347e-01<b1<-8.108e-02
2.541e-01<sigma<9.853e-01

Layer: Media
Substance: Platelet
Regressand: Median

Model
'50%-quantile'=b0+(b1)x'Treatment_time'+eps
b0: 7.061e+00%
analytical 95% confidence interval: 5.481e+00<b0<8.642e+00
b1: -1.009e-01%/week
analytical 95% confidence interval: -2.181e-01<b1<1.621e-02
standard deviation of eps (sigma): 5.643e-01%
analytical 95% confidence interval: 2.938e-01<sigma<3.546e+00
R-square: 8.731e-01
adjusted R-square: 8.096e-01

Hypothesis Testing
H0: 'b1 is equal to 0%/week'
H1: 'b1 is different from 0%/week'
analytical t-test p-value: 6.567e-02
fuzzy Bootstrap Probability for b1 to be non-negative calculated with 10000 pseudo-realities:
quasi-equal-information generation from ECDF: 0.000e+00
quasi-equal-information generation from FECDF: 0.000e+00
equal-size generation from ECDF: 0.000e+00
equal-size generation from FECDF: 0.000e+00

fuzzy Bootstrap 95% confidence intervals estimated with 10000 pseudo-realities:
quasi-equal-information generation from ECDF:
6.568e+00<b0<7.536e+00
-1.415e-01<b1<-6.704e-02
1.437e-01<sigma<1.216e+00
quasi-equal-information generation from FECDF:
6.871e+00<b0<7.690e+00
-1.516e-01<b1<-8.066e-02
1.622e-01<sigma<1.073e+00
equal-size generation from ECDF:
6.575e+00<b0<7.535e+00
-1.412e-01<b1<-6.792e-02
1.416e-01<sigma<1.211e+00
equal-size generation from FECDF:
6.888e+00<b0<7.637e+00
-1.501e-01<b1<-8.152e-02
1.543e-01<sigma<1.047e+00

Layer: Media
Substance: Platelet
Regressand: Upper Quartile

Model
'75%-quantile'=b0+(b1)x'Treatment_time'+eps
b0: 1.151e+01%
analytical 95% confidence interval: 7.976e+00<b0<1.505e+01
b1: -1.467e-01%/week
analytical 95% confidence interval: -3.603e-01<b1<6.696e-02
standard deviation of eps (sigma): 1.006e+00%
analytical 95% confidence interval: 5.237e-01<sigma<6.322e+00
R-square: 8.310e-01
adjusted R-square: 7.464e-01

Hypothesis Testing
H0: 'b1 is equal to 0%/week'
H1: 'b1 is different from 0%/week'
analytical t-test p-value: 9.803e-02
fuzzy Bootstrap Probability for b1 to be non-negative calculated with 10000 pseudo-realities:
quasi-equal-information generation from ECDF: 0.000e+00
quasi-equal-information generation from FECDF: 0.000e+00
equal-size generation from ECDF: 5.000e-04
equal-size generation from FECDF: 4.000e-04

fuzzy Bootstrap 95% confidence intervals estimated with 10000 pseudo-realities:
quasi-equal-information generation from ECDF:
9.897e+00<b0<1.321e+01
-2.373e-01<b1<-6.415e-02
2.578e-01<sigma<2.381e+00
quasi-equal-information generation from FECDF:
1.008e+01<b0<1.416e+01
-2.700e-01<b1<-6.470e-02
4.530e-01<sigma<3.318e+00
equal-size generation from ECDF:
9.853e+00<b0<1.319e+01
-2.380e-01<b1<-6.340e-02
2.513e-01<sigma<2.347e+00
equal-size generation from FECDF:
1.013e+01<b0<1.409e+01
-2.696e-01<b1<-6.754e-02
4.803e-01<sigma<3.244e+00

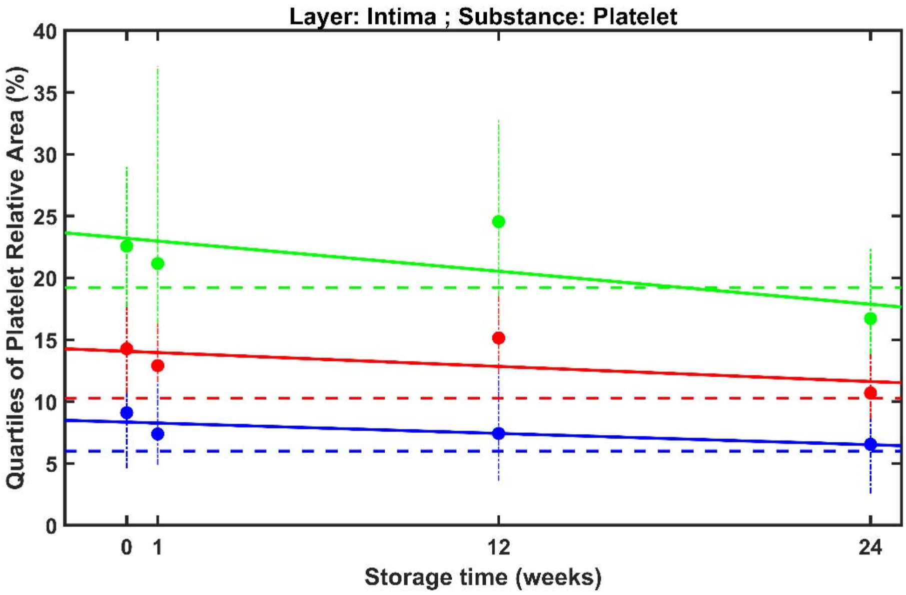

Layer: Intima
Substance: Platelet
Regressand: Lower Quartile

Model
'25%-quantile'=b0+(b1)x'Treatment_time'+eps
b0: 8.380e+00%
analytical 95% confidence interval: 5.929e+00<b0<1.083e+01
b1: -7.791e-02%/week
analytical 95% confidence interval: -2.520e-01<b1<9.616e-02
standard deviation of eps (sigma): 8.501e-01%
analytical 95% confidence interval: 4.426e-01<sigma<5.343e+00
R-square: 6.501e-01
adjusted R-square: 4.752e-01

Hypothesis Testing
H0: 'b1 is equal to 0%/week'
H1: 'b1 is different from 0%/week'
analytical t-test p-value: 1.940e-01
fuzzy Bootstrap Probability for b1 to be non-negative calculated with 10000 pseudo-realities:
quasi-equal-information generation from ECDF: 7.560e-02
quasi-equal-information generation from FECDF: 1.843e-01
equal-size generation from ECDF: 7.090e-02
equal-size generation from FECDF: 1.708e-01

fuzzy Bootstrap 95% confidence intervals estimated with 10000 pseudo-realities:
quasi-equal-information generation from ECDF:
6.253e+00<b0<9.718e+00
-1.864e-01<b1<2.780e-02
2.093e-01<sigma<2.551e+00
quasi-equal-information generation from FECDF:
5.281e+00<b0<8.874e+00
-1.619e-01<b1<5.159e-02
2.470e-01<sigma<2.755e+00

equal-size generation from ECDF:
 6.302e+00<b0<9.745e+00
 -1.844e-01<b1<2.598e-02
 2.151e-01<sigma<2.544e+00
equal-size generation from FECDF:
 5.392e+00<b0<8.793e+00
 -1.590e-01<b1<4.892e-02
 2.441e-01<sigma<2.724e+00

Layer: Intima
Substance: Platelet
Regressand: Median

Model
'50%-quantile'=b0+(b1)x'Treatment_time'+eps
b0: 1.391e+01%
analytical 95% confidence interval: 8.429e+00<b0<1.939e+01
b1: -9.952e-02%/week
analytical 95% confidence interval: -4.941e-01<b1<2.951e-01
standard deviation of eps (sigma): 1.843e+00%
analytical 95% confidence interval: 9.594e-01<sigma<1.158e+01
R-square: 3.914e-01
adjusted R-square: 8.707e-02

Hypothesis Testing
H0: 'b1 is equal to 0%/week'
H1: 'b1 is different from 0%/week'
analytical t-test p-value: 3.913e-01
fuzzy Bootstrap Probability for b1 to be non-negative calculated with 10000 pseudo-realities:
quasi-equal-information generation from ECDF: 5.890e-02
quasi-equal-information generation from FECDF: 1.143e-01
equal-size generation from ECDF: 5.470e-02
equal-size generation from FECDF: 1.002e-01

fuzzy Bootstrap 95% confidence intervals estimated with 10000 pseudo-realities:
quasi-equal-information generation from ECDF:
 1.255e+01<b0<1.629e+01
 -2.237e-01<b1<2.465e-02
 5.449e-01<sigma<3.619e+00
quasi-equal-information generation from FECDF:
 1.142e+01<b0<1.477e+01
 -1.885e-01<b1<4.944e-02
 3.372e-01<sigma<2.858e+00
equal-size generation from ECDF:
 1.254e+01<b0<1.629e+01
 -2.241e-01<b1<2.265e-02
 5.633e-01<sigma<3.694e+00
equal-size generation from FECDF:
 1.150e+01<b0<1.461e+01
 -1.820e-01<b1<4.328e-02
 3.575e-01<sigma<2.810e+00

Layer: Intima
Substance: Platelet
Regressand: Upper Quartile

Model
'75%-quantile'=b0+(b1)x'Treatment_time'+eps
b0: 2.332e+01%
analytical 95% confidence interval: 1.394e+01<b0<3.270e+01
b1: -2.454e-01%/week
analytical 95% confidence interval: -7.854e-01<b1<2.946e-01
standard deviation of eps (sigma): 2.693e+00%
analytical 95% confidence interval: 1.402e+00<sigma<1.693e+01
R-square: 7.026e-01
adjusted R-square: 5.539e-01

Hypothesis Testing
H0: 'b1 is equal to 0%/week'
H1: 'b1 is different from 0%/week'
analytical t-test p-value: 1.897e-01
fuzzy Bootstrap Probability for b1 to be non-negative calculated with 10000 pseudo-realities:
quasi-equal-information generation from ECDF: 2.480e-02
quasi-equal-information generation from FECDF: 3.770e-02
equal-size generation from ECDF: 2.180e-02
equal-size generation from FECDF: 2.820e-02

fuzzy Bootstrap 95% confidence intervals estimated with 10000 pseudo-realities:
quasi-equal-information generation from ECDF:
1.956e+01<b0<2.605e+01
-4.016e-01<b1<-1.745e-04
6.164e-01<sigma<5.315e+00
quasi-equal-information generation from FECDF:
1.796e+01<b0<2.461e+01
-3.866e-01<b1<1.830e-02
3.595e-01<sigma<4.051e+00
equal-size generation from ECDF:
1.972e+01<b0<2.620e+01
-4.093e-01<b1<-4.901e-03
6.242e-01<sigma<5.271e+00
equal-size generation from FECDF:
1.819e+01<b0<2.449e+01
-3.814e-01<b1<5.270e-03
3.741e-01<sigma<3.866e+00
