# Supplementary material for: Cryopreservation moderates the thrombogenicity of arterial allografts during storage
Source: PLoS One. 2021 Jul 22;16(7):e0255114. doi: 10.1371/journal.pone.0255114 (PMC8297765; doi:10.1371/journal.pone.0255114)
Supplement: S1 Data — (ZIP) [file pone.0255114.s002.zip › Open_data_R1/Qualitative_quartile_wise_comparisons_R1.pdf]

Data for the non-transitive pairwise comparisons of the thrombogenic indicators of the grafts

For each combination of layer and substance (six in total), we test the lower quartile (LQR), the median (MED) and the upper quartile (UQR) at the different storage times (ST, which are 0, 1, 12, and 24 weeks). We conduct four fuzzy Bootstrap tests, which form the cluster $Cl_{hyp,k}$ (described in Supporting_information1.pdf) with N=1000 pseudo-realities each. The p-values of the tests are presented in a matrix where each cell contains:

1) the tests in the first row are with quasi-equal information generation, while in the second - with equal information generation

2) the tests in the first column are with information generated from empirical cumulative distribution function (ECDF), while in the second – from fuzzy cumulative distribution function (FECDF).

The tables include only the tests that rejected H_0 at p_{value}<=0.05. For each comparison, if at least two out of the four tests reject H_0 with p_{value}<=0.05, then we assume that there is a statistically significant difference between the analyzed parameters. Even if the other two tests do not detect difference, they do not claim that the parameters are equal, but simply claim that there is not enough information to reject the equality.

In order to compare the power of the applied fuzzy analysis with a classic crisp sample bootstrap approach, for each combination of layer and substance we use the same datasets treating them as crisp samples. We conduct one crisp bootstrap test with N=1000 pseudo-realities each and present its p-value in a matrix. The results of the comparison of the two approaches are color-coded in the tables below. If a cell is shown in green, the crisp bootstrap test and the cluster $Cl_{hyp,k}$ produce identical results. If a cell is shown in red, the crisp Bootstrap test and the cluster $Cl_{hyp,k}$ produce different results. If the relative ranks of any two quantile from the fuzzy sample are different from the relative ranks of the same two quantiles from the crisp sample, then the respective cell is shown in purple.

Summarizing the comparisons indicated in the tables, the crisp Bootstrap test gives the same results as the fuzzy Bootstrap test in 73.3% of the comparisons (132 out of 180). This result demonstrates the robust nature of the cluster $Cl_{hyp,k}$. On the other hand, the crisp sample produce 16.1% discrepancies (29 out of 180) and 10.6% rank permutations (19 out of 180) in relation to the fuzzy samples. Those two ratios underline the need to use fuzzy bootstrap cluster ($Cl_{hyp,k}$) instead of a single crisp bootstrap test.

Layer: Adventitia ; Substance: Fibrin

	LQR0 (8.8%)	LQR24 (6.4%)	LQR12 (5.6%)	LQRn (8.4%)
LQR1 (11.1%)		1.15e-02<br>1.24e-02	3.90e-03<br>5.40e-03	
LQR0 (8.8%)			1.74e-02<br>1.70e-02	
LQR24 (6.4%)				
LQR12 (5.6%)				

Layer: Adventitia ; Substance: Fibrin

	MED0 (18.6%)	MED24 (14.4%)	MED12 (11.4%)	MEDn (19.7%)
MED1 (19.3%)		3.00e-02 3.230e-02<br>3.15e-02 2.540e-02	1.10e-03 0.00e+00<br>5.00e-04 3.00e-04	
MED0 (18.6%)			5.00e-04<br>6.00e-04	
MED24 (14.4%)			4.29e-02<br>2.72e-02	
MED12 (11.4%)				

Layer: Adventitia ; Substance: Fibrin

	UQR0 (29.4%)	UQR24 (22.2%)	UQR12 (18.6%)	UQRn (40.6%)
UQR1 (33.5%)		3.60e-03 3.10e-03<br>3.60e-03 3.00e-03	1.00e-04 1.00e-04<br>1.00e-04 0.00e+00	
UQR0 (29.4%)			9.00e-04 3.80e-02<br>1.30e-03 2.61e-02	1.52e-02 1.33e-02<br>1.59e-02 7.40e-03
UQR24 (22.2%)			3.28e-02<br>3.45e-02	0.00e+00 1.00e-04<br>0.00e+00 1.00e-04
UQR12 (18.6%)				0.00e+00 0.00e+00<br>0.00e+00 0.00e+00

Of all three quartiles the values at ST1 are always the highest, followed by slightly lower ST0, then the ST24, and last are the ST12 (slightly lower than for ST24).

The comparisons with the no treatment (NT) values show no statistically significant difference for the LQR and MED. Only UQR has statistically significantly higher values with NT for ST0, ST24 and ST12, confirmed by all 4 tests.

There is no statistically significant difference between the ST0 and ST1 values, but for all three quartiles, ST1 values are statistically higher than for ST24 and for ST12, while the ST0 values are statistically higher than ST12. Yet, there is no difference between the ST0 and ST24 values.

The ST12 and ST24 values have statistically equal LQRs, while for the MED and UQR, the ST24 values are statistically higher than those for ST12.

As a whole, there is no transitivity of the statistically significant relations. There is no statistical difference between the quartiles for ST0 and ST1, while the difference between the ST12 and ST24 values is negligible. The quartiles for ST0 and ST1 are high at the beginning with their values decreasing in ST12 and ST24.

The crisp sample results are:

Layer: Adventitia ; Substance: Fibrin ; Data: crisp samples

	LQR0 (7.4%)	LQR24 (6.3%)	LQR12 (4.9%)	LQRn (6.0%)
LQR1 (11.7%)	7.00e-3	1.00e-03	0.00e+00	2.00e-3
LQR0 (7.4%)		>0.05	>0.05	>0.05
LQR24 (6.3%)			>0.05	>0.05
LQR12 (4.9%)				>0.05

Layer: Adventitia ; Substance: Fibrin ; Data: crisp samples

	MED24 (14.4%)	MED0 (14.0%)	MED12 (10.7%)	MEDn (15.2%)
MED1 (20.3%)	2.00e2	2.90e-02	0.00e+00	>0.05
MED24 (14.4%)		>0.05	1.7e-02	>0.05
MED0 (14.0%)			>0.05	>0.05
MED12 (10.7%)				4.50e-02

Layer: Adventitia ; Substance: Fibrin ; Data: crisp samples

	UQR0 (24.9%)	UQR24 (23.0%)	UQR12 (18.0%)	UQRn (35.5%)
UQR1 (33.7%)	2.80e-2	1.10e-2	0.00e+00	>0.05
UQR0 (24.9%)		>0.05	5.00e-03	5.00e-03
UQR24 (23.0%)			5.00e-03	1.00e-03
UQR12 (18.0%)				0.00e+00

The crisp samples give the same results as the fuzzy samples in 66.7% of the comparisons. However, the former produce 23.3% discrepancies and 10.0% rank permutations in relation to the fuzzy samples.

Layer: Media ; Substance: Fibrin

	LQR24 (0.7%)	LQR0 (0.6%)	LQR12 (0.4%)	LQRn (1.8%)
LQR1 (1.4%)	2.49e-02<br>2.44e-02	2.25e-02<br>2.63e-02	3.10e-03 3.93e-02<br>2.30e-03 2.91e-02	
LQR24 (0.7%)				2.70e-03<br>2.90e-03
LQR0 (0.6%)				3.50e-03<br>2.90e-03
LQR12 (0.4%)				6.00e-04<br>1.00e-04

Layer: Media ; Substance: Fibrin

	MED0 (3.7%)	MED24 (2.3%)	MED12 (2.0%)	MEDn (5.3%)
MED1 (4.8%)		3.63e-02<br>3.61e-02	2.09e-02<br>2.21e-02	
MED0 (3.7%)				
MED24 (2.3%)				3.00e-03<br>1.00e-03
MED12 (2.0%)				1.60e-03<br>9.00e-04

Layer: Media ; Substance: Fibrin

	UQR1 (12.5%)	UQR24 (6.9%)	UQR12 (4.7%)	UQRn (15.2%)
UQR0 (13.7%)		2.88e-02<br>2.88e-02	3.10e-03 3.45e-02<br>3.10e-03 2.87e-02	
UQR1 (12.5%)		1.70e-03 1.17e-02<br>1.20e-03 8.30e-03	0.00e+00 1.60e-03<br>0.00e+00 9.00e-04	
UQR24 (6.9%)			1.97e-02<br>1.73e-02	1.60e-03<br>1.00e-03
UQR12 (4.7%)				0.00e+00 1.56e-02<br>0.00e+00 1.10e-02

For all quartiles, the values for NT are the highest, with the treatment parameters being lower (statistically significant or not). For all quartiles, the NT gives higher values than for ST12 and ST24, but it has no difference with ST1. NT gives statistically the same results as ST0 for MED and UQR, but is statistically higher for the LQR.

For the three quartiles, the ST1 values are higher than ST24, while the ST1 values are higher than for ST12. Only for the LQR, ST1 values are higher than for ST0, whereas for the MED and UQR they are statistically the same.

For the MED and LQR, the ST0 values are the same for ST12 and for ST24, but for the UQR, ST0 values are higher from those for ST12 and for ST24.

For the MED and the LQR, ST24 values are statistically the same as the ST12 values, but for the UQR, ST24 values are statistically higher than ST12.

As a whole, transitivity cannot be identified.

The crisp sample results are:

Layer: Media ; Substance: Fibrin ; Data: crisp samples

	LQR24 (0.5%)	LQR0 (0.5%)	LQR12 (0.4%)	LQRn (1.4%)
LQR1 (1.2%)	7.00e-03	1.00e-02	4.00e-03	>0.05
LQR24 (0.5%)		>0.05	>0.05	1.00e-03
LQR0 (0.5%)			>0.05	4.00e-03
LQR12 (0.4%)				0.00e+00

Layer: Media ; Substance: Fibrin ; Data: crisp samples

	MED0 (2.8%)	MED12 (1.9%)	MED24 (1.8%)	MEDn (3.9%)
MED1 (3.9%)	>0.05	1.90e-02	1.80e-02	>0.05
MED0 (2.8%)		>0.05	>0.05	>0.05
MED12 (1.9%)			>0.05	3.00e-3
MED24 (1.8%)				0.00e+00

Layer: Media ; Substance: Fibrin ; Data: crisp samples

	UQR1 (11.0%)	UQR24 (5.9%)	UQR12 (4.7%)	UQRn (10.1%)
UQR0 (11.9%)	>0.05	0.00e+00	1.00e-03	>0.05
UQR1 (11.0%)		0.00e+00	0.00e+00	>0.05
UQR24 (5.9%)			>0.05	>0.05
UQR12 (4.7%)				1.30e-02

The crisp samples give the same results as the fuzzy samples in 86.7% of the comparisons. However, the former produce 6.67% discrepancies and 6.67% rank permutations in relation to the fuzzy samples.

Layer: Intima ; Substance: Fibrin

	LQR0 (6.4%)	LQR24 (5.2%)	LQR12 (3.9%)	LQRn (6.2%)
LQR1 (6.9%)				
LQR0 (6.4%)				
LQR24 (5.2%)				
LQR12 (3.9%)				

Layer: Intima ; Substance: Fibrin

	MED0 (17.2%)	MED24 (13.2%)	MED12 (10.9%)	MEDn (19.3%)
MED1 (21.0%)		6.30e-03 5.00e-03<br>8.00e-03 3.20e-03	3.30e-03 1.60e-03<br>1.90e-03 6.00e-04	
MED0 (17.2%)				
MED24 (13.2%)				1.64e-02<br>1.67e-02
MED12 (10.9%)				5.00e-03 4.22e-02<br>4.70e-03 2.88e-02

Layer: Intima ; Substance: Fibrin

	UQR1 (32.0%)	UQR12 (27.2%)	UQR24 (20.6%)	UQRn (29.4%)
UQR0 (34.4%)			5.00e-03 1.17e-02<br>4.20e-03 8.50e-03	
UQR1 (32.0%)			6.20e-03 2.60e-03<br>4.90e-03 1.30e-03	
UQR12 (27.2%)				
UQR24 (20.6%)				2.30e-03 2.32e-02<br>1.60e-03 1.70e-02

For all quartiles, the NT values are lower than those for ST1 and ST0, but higher than for ST12 and ST24, with the only marginal and statistically insignificant exception for the MED, when the ST0 is lower than NT. For all quartiles, there is no statistical difference between the NT and the ST0 and ST1 values. For the LQR and UQR, there is no statistical difference between the NT and ST12, but NT is statistically higher than ST12 for MED. For MED and UQR, the NT is statistically higher than ST24, but at the LQR they are statistically equal.

For the three quartiles, ST1 is statistically equal to ST0. For the MED and the UQR, ST1 is statistically higher than ST24, but statistically equal for the LQR. For the LQR and the UQR, ST1 values are statistically equal to those for ST12, but the ST1 value is statistically higher than ST12 for the MED.

For the LQR and the MED, the ST0 values are statistically equal to ST24, but the ST0 value is higher than the ST24 for UQR. For all quartiles, the ST0 values are statistically equal to ST12.

For all quartiles, ST12 values are statistically equal to ST24 values.

As a whole, transitivity is present only for the LQR, where there is no statistical difference depending on storage time. The quartiles at ST0 and ST1 are higher than the quartiles for ST12 and ST24.

The crisp sample results are:

`Layer: Intima ; Substance: Fibrin ; Data: crisp samples`

	LQR0 (6.4%)	LQR12 (5.2%)	LQR24 (3.9%)	LQRn (6.2%)
LQR1 (6.9%)	>0.05	>0.05	>0.05	>0.05
LQR0 (6.4%)		>0.05	>0.05	>0.05
LQR12 (5.2%)			>0.05	>0.05
LQR24 (3.9%)				>0.05

`Layer: Intima ; Substance: Fibrin ; Data: crisp samples`

	MED0 (18.2%)	MED24 (13.7%)	MED12 (13.3%)	MEDn (14.8%)
MED1 (21.9%)	>0.05	7.00e-03	1.00e-02	>0.05
MED0 (16.2%)		>0.05	>0.05	>0.05
MED24 (13.7%)			>0.05	>0.05
MED12 (13.3%)				>0.05

`Layer: Intima ; Substance: Fibrin ; Data: crisp samples`

	UQR1 (32.7%)	UQR12 (27.8%)	UQR24 (20.8%)	UQRn (26.9%)
UQR0 (33.9%)	>0.05	>0.05	2.00e-03	>0.05
UQR1 (32.7%)		>0.05	1.30e-03	>0.05
UQR12 (27.8%)			1.60e-02	>0.05
UQR24 (20.8%)				>0.05

The crisp samples give the same results as the fuzzy samples in 76.7% of the comparisons. However, the former produce 13.3% discrepancies and 10.0% rank permutations in relation to the fuzzy samples.

Layer: Adventitia ; Substance: Platelet

	LQR1 (10.8%)	LQR12 (10.3%)	LQR24 (8.8%)	LQRn (12.2%)
LQR0 (13.0%)			2.00e-04 0.00e+00<br>2.00e-04 1.00e-04	
LQR1 (10.8%)			4.93e-02	
LQR12 (10.3%)				
LQR24 (8.8%)				6.00e-04 4.20e-03<br>4.00e-04 2.70e-03

Layer: Adventitia ; Substance: Platelet

	MED1 (17.5%)	MED12 (16.3%)	MED24 (13.1%)	MEDn (17.8%)
MED0 (17.6%)			3.00e-04 2.30e-03<br>0.00e+00 1.30e-03	
MED1 (17.5%)			2.50e-03 1.56e-02<br>1.90e-03 9.20e-03	
MED12 (16.3%)			7.20e-03 9.70e-03<br>8.20e-03 5.20e-03	
MED24 (13.1%)				6.00e-04 1.00e-04<br>5.00e-04 5.00e-04

Layer: Adventitia ; Substance: Platelet

	UQR1 (26.7%)	UQR12 (24.6%)	UQR24 (19.0%)	UQRn (25.2%)
UQR0 (27.2%)			6.00e-04 1.62e-02<br>6.00e-04 1.22e-02	
UQR1 (26.7%)			0.00e+00 4.00e-04<br>2.00e-04 0.00e+00	
UQR12 (24.6%)			1.00e-04 1.00e-04<br>5.00e-04 1.00e-04	
UQR24 (19.0%)				6.00e-04 4.00e-04<br>3.00e-04 1.00e-04

For all three quartiles, as ST increases their values decrease.

The NT results are statistically higher than those for ST24 on all quartiles and have no statistically significant difference compared to the ST0, ST1 and ST12 values (on 4 tests).

For the MED and the UQR, {ST0, ST1, ST12} form a group with statistically higher values (on 4 tests) than for ST24 (i.e there is transitivity). For the LQR, all ST values are statistically equal except that ST0 is higher than ST24 (on 4 tests).

As a whole, the transitivity is compromised slightly only for the LQR. The quartiles at ST0, ST1 and ST12 are statistically higher than at ST24. The differences between the ST0, ST1 and ST12 values are negligible.

The crisp sample results are:

```
Layer: Adventitia ; Substance: Platelet ; Data: crisp samples
```

	LQR0 (11.1%)	LQR12 (9.9%)	LQR24 (8.5%)	LQRn (11.6%)
LQR1 (11.5%)	>0.05	>0.05	9.00e-03	>0.05
LQR0 (11.1%)		>0.05	9.00e-03	>0.05
LQR12 (9.9%)			3.80e-2	>0.05
LQR24 (8.5%)				5.00e-03

```
Layer: Adventitia ; Substance: Platelet ; Data: crisp samples
```

	MED0 (16.9%)	MED12 (16.0%)	MED24 (13.0%)	MEDn (16.3%)
MED1 (18.2%)	>0.05	>0.05	0.00e+00	>0.05
MED0 (16.9%)		>0.05	0.00e+00	>0.05
MED12 (16.0%)			1.40e-02	>0.05
MED24 (13.2%)				1.00e-02

```
Layer: Adventitia ; Substance: Platelet ; Data: crisp samples
```

	UQR0 (25.8%)	UQR12 (24.6%)	UQR24 (19.2%)	UQRn (23.6%)
UQR1 (27.1%)	>0.05	>0.05	0.00e+00	>0.05
UQR0 (25.8%)		>0.05	1.10e-02	>0.05
UQR12 (24.6%)			1.00e-03	>0.05
UQR24 (19.2%)				2.00e-03

The crisp samples give the same results as the fuzzy samples in 63.3% of the comparisons. However, the former produce 13.3% discrepancies and 23.3% rank permutations in relation to the fuzzy samples.

Layer: Media ; Substance: Platelet

	LQR0 (3.6%)	LQR12 (3.0%)	LQR24 (2.4%)	LQRn (2.5%)
LQR1 (5.2%)	7.00e-04 2.22e-02<br>8.00e-04 1.59e-02	0.00e+00 0.00e+00<br>0.00e+00 0.00e+00	0.00e+00 0.00e+00<br>0.00e+00 0.00e+00	0.00e+00 0.00e+00<br>0.00e+00 0.00e+00
LQR0 (3.6%)			3.00e-03<br>3.50e-03	1.48e-02<br>1.43e-02
LQR12 (3.0%)				
LQR24 (2.4%)				

Layer: Media ; Substance: Platelet

	MED12 (6.8%)	MED0 (6.5%)	MED24 (4.5%)	MEDn (3.8%)
MED1 (7.0%)			0.00e+00 0.00e+00<br>0.00e+00 0.00e+00	0.00e+00 0.00e+00<br>0.00e+00 0.00e+00
MED12 (6.8%)			9.00e-04 9.00e-04<br>1.10e-03 5.00e-04	0.00e+00 1.00e-04<br>0.00e+00 1.00e-04
MED0 (6.5%)			1.00e-03<br>1.70e-03	0.00e+00 5.90e-03<br>0.00e+00 3.40e-03
MED24 (4.5%)				

Layer: Media ; Substance: Platelet

	UQR12 (11.0%)	UQR0 (10.7%)	UQR24 (7.6%)	UQRn (6.7%)
UQR1 (11.1%)			2.50e-03 1.14e-02<br>2.30e-03 1.08e-02	1.20e-03 7.70e-03<br>1.30e-03 3.80e-03
UQR12 (11.0%)			2.40e-03 7.60e-03<br>2.20e-03 6.50e-03	7.00e-04 5.00e-04<br>4.00e-04 4.00e-04
UQR0 (10.7%)			4.83e-02	1.49e-02<br>1.31e-02
UQR24 (7.6%)				

For all quartiles, the NT values are the lowest, with the treatment parameters being higher (whether statistically significant or not). The only exception is that for the LQR, the NT value is marginally and statistically insignificantly higher. For all quartiles, NT have lower values than ST1 and ST0, and is statistically the same as ST24. For MED and UQR, the NT values are lower than for ST12, but statistically the same for the LQR (on 4 tests).

For the three quartiles, ST1 always has the highest values, followed by ST 0 and ST24, with the only exception for marginally and statistically insignificant ST0 value higher than ST12 for the LQR.

For the three quartiles, ST1 is statistically higher than ST24 (on 4 tests). For the MED and UQR, ST1 is statistically equal to ST0 and ST12, but for the LQR, ST1 is statistically higher than ST0, while ST1 is statistically higher than ST12.

For the three quartiles, the ST12 values are statistically equal to ST0. For the MED and the UQR, ST12 values are statistically higher than for ST24, but for the LQR, the ST12 is equal to ST24.

For the LQR and the MED, ST0 values are statistically higher than ST24, but for the UQR, the ST0 value is statistically equal to ST24.

There is no transitivity in this combination as a whole, and the quartiles at ST1, ST12 and ST0 tend to be statistically higher than for ST24, while also being almost equal in between (with the exception at the LQR).

The crisp sample results are:

`Layer: Media ; Substance: Platelet ; Data: crisp samples`

	LQR0 (3.3%)	LQR12 (3.0%)	LQR24 (2.5%)	LQRn (2.2%)
LQR1 (5.1%)	0.00e+00	0.00e+00	0.00e+00	0.00e+00
LQR0 (3.3%)		>0.05	3.60e-02	6.00e-03
LQR12 (3.0%)			>0.05	2.80e-02
LQR24 (2.5%)				>0.05

`Layer: Media ; Substance: Platelet ; Data: crisp samples`

	MED12 (6.8%)	MED0 (5.8%)	MED24 (4.5%)	MEDn (3.6%)
MED1 (7.0%)	>0.05	>0.05	0.00e+00	0.00e+00
MED12 (6.8%)		>0.05	1.00e-03	0.00e+00
MED0 (5.8%)			2.30e-03	0.00e+00
MED24 (4.5%)				3.40e-02

`Layer: Media ; Substance: Platelet ; Data: crisp samples`

	UQR12 (11.0%)	UQR0 (10.7%)	UQR24 (7.6%)	UQRn (6.7%)
UQR1 (11.1%)	>0.05	>0.05	0.00e+00	0.00e+00
UQR12 (11.0%)		>0.05	8.00e-03	1.00e-03
UQR0 (10.7%)			1.60e-02	4.00e-03
UQR24 (7.6%)				>0.05

The crisp samples give the same results as the fuzzy samples in 83.3% of the comparisons. However, the former produce 13.3% discrepancies and 3.33% rank permutations in relation to the fuzzy samples.

Layer: Intima ; Substance: Platelet

	LQR12 (7.4%)	LQR1 (7.4%)	LQR24 (6.5%)	LQRn (6.0%)
LQR0 (9.1%)				
LQR12 (7.4%)				
LQR1 (7.4%)				
LQR24 (6.5%)				

Layer: Intima ; Substance: Platelet

	MED0 (14.3%)	MED1 (12.9%)	MED24 (10.7%)	MEDn (10.3%)
MED12 (15.1%)			8.00e-03<br>7.20e-03 4.83e-02	8.00e-04 1.12e-02<br>8.00e-04 7.50e-03
MED0 (14.3%)			4.82e-02<br>4.42e-02	1.31e-02 4.59e-02<br>1.22e-02 3.64e-02
MED1 (12.9%)				4.15e-02 1.95e-02<br>4.64e-02 1.27e-02
MED24 (10.7%)				

Layer: Intima ; Substance: Platelet

	UQR0 (22.6%)	UQR1 (21.2%)	UQR24 (16.7%)	UQRn (19.2%)
UQR12 (24.6%)			1.05e-02<br>9.40e-03	
UQR0 (22.6%)			1.75e-02 2.39e-02<br>1.78e-02 1.77e-02	
UQR1 (21.2%)				
UQR24 (16.7%)				

For all quartiles, the NT values are the lowest, with the only marginal and statistically insignificant exception for the UQR, where NT gives higher values than for ST24. For the LQR and the UQR, there is no difference between treatment and no treatment. For the MED, the NT values are statistically lower than for ST0, ST1 and ST12.

For all quartiles, ST12 values are higher than ST0, ST0 are higher ST1, and ST1 are higher than ST24. There is the only marginal and statistically insignificant exception for the LQR, where the value for ST0 is higher than for ST12.

For all quartiles, ST12, ST0 and ST1 values are statistically equal, exactly as ST1 and ST24 values.

For the MED and the UQR, the ST24 values are statistically lower than the ST12 values, while ST24 values are statistically lower than thr ST0 values. For the UQR, ST12, ST24 and ST0 values are statistically equal.

As a whole, ST12, ST0 and ST1 values are statistically equal and tend to be higher than the ST24 values. Transitivity is present at the LQR, and almost present for the MED and the UQR.

The crisp sample results are:

Layer: Intima ; Substance: Platelet ; Data: crisp samples

	LQR0 (9.7%)	LQR1 (7.5%)	LQR24 (6.6%)	LQRn (6.0%)
LQR12 (10.7%)	>0.05	>0.05	5.00e-03	5.00e-03
LQR0 (9.7%)		>0.05	1.67e-02	2.00e-02
LQR1 (7.5%)			>0.05	>0.05
LQR24 (6.5%)				>0.05

Layer: Intima ; Substance: Platelet ; Data: crisp samples

	MED0 (15.5%)	MED1 (13.7%)	MED24 (11.4%)	MEDn (10.3%)
MED12 (17.4%)	>0.05	>0.05	0.00e+00	0.00e+00
MED0 (15.5%)		>0.05	1.40e-02	2.00e-03
MED1 (13.7%)			>0.05	1.90e-022
MED24 (11.4%)				>0.05

Layer: Intima ; Substance: Platelet ; Data: crisp samples

	UQR1 (24.2%)	UQR0 (22.1%)	UQR24 (18.4%)	UQRn (17.0%)
UQR12 (26.1%)	>0.05	>0.05	3.10e-02	9.00e-03
UQR1 (24.2%)		>0.05	4.70e-02	4.30e-02
UQR0 (22.1%)			>0.05	4.50e-02
UQR24 (18.4%)				>0.05

The crisp samples give the same results as the fuzzy samples in 60.0% of the comparisons. However, the former produce 30.0% discrepancies and 10.0% rank permutations in relation to the fuzzy samples.
